# Dose of physical activity, physical functioning and disability risk in mobility-limited older adults: Results from the LIFE study randomized trial

**DOI:** 10.1371/journal.pone.0182155

**Published:** 2017-08-18

**Authors:** Roger A. Fielding, Jack M. Guralnik, Abby C. King, Marco Pahor, Mary M. McDermott, Catrine Tudor-Locke, Todd M. Manini, Nancy W. Glynn, Anthony P. Marsh, Robert S. Axtell, Fang-Chi Hsu, W. Jack Rejeski, for the LIFE study group

**Affiliations:** 1 Nutrition, Exercise Physiology, and Sarcopenia Laboratory, Jean Mayer USDA Human Nutrition Research Center on Aging at Tufts University, Boston, MA, United States of America; 2 Department of Epidemiology and Public Health, University of Maryland School of Medicine, 4655 W. Baltimore Street, Baltimore, MD, United States of America; 3 Department of Health Research & Policy, and Stanford Prevention Research Center, Department of Medicine, Stanford University Medical School, Stanford, CA, United States of America; 4 Department of Aging and Geriatric Research, University of Florida College of Medicine, Gainesville, FL, United States of America; 5 Department of Medicine, Northwestern University Feinberg School of Medicine, 750 N Lake Shore Drive, 10th Floor, Chicago, IL, United States of America; 6 Department of Kinesiology, University of Massachusetts Amherst, Amherst, MA, United States of America; 7 Pennington Biomedical Research Center, Louisiana State University System, Baton Rouge, LA, United States of America; 8 Center for Aging and Population Health, Department of Epidemiology, Graduate School of Public Health, University of Pittsburgh, Pittsburgh, PA, United States of America; 9 Department of Health and Exercise Science, Wake Forest University, Winston-Salem, NC, United States of America; 10 Department of Exercise Science, Southern Connecticut State University, New Haven, CT; 11 Department of Biostatistical Sciences, Division of Public Health Sciences, Wake Forest School of Medicine, Winston-Salem, NC, United States of America; Victoria University, AUSTRALIA

## Abstract

Understanding the minimal dose of physical activity required to achieve improvement in physical functioning and reductions in disability risk is necessary to inform public health recommendations. To examine the effect of physical activity dose on changes in physical functioning and the onset of major mobility disability in The Lifestyle Interventions and Independence for Elders (LIFE) Study. We conducted a multicenter single masked randomized controlled trial that enrolled participants in 2010 and 2011 and followed them for an average of 2.6 years. 1,635 sedentary men and women aged 70–89 years who had functional limitations were randomized to a structured moderate intensity walking, resistance, and flexibility physical activity program or a health education program. Physical activity dose was assessed by 7-day accelerometry and self-report at baseline and 24 months. Outcomes included the 400 m walk gait speed, the Short Physical Performance Battery (SPPB), assessed at baseline, 6, 12, and 24 months, and onset of major mobility disability (objectively defined by loss of ability to walk 400 m in 15 min). When the physical activity arm or the entire sample were stratified by change in physical activity from baseline to 24 months, there was a dose-dependent increase in the change in gait speed and SPPB from baseline at 6, 12, and 24 months. In addition, the magnitude of change in physical activity over 24 months was related to the reduction in the onset of major mobility disability (overall P < 0.001) (highest versus the lowest quartile of physical activity change HR 0.23 ((95% CI:0.10–0.52) P = 0.001) in the physical activity arm. We observed a dose-dependent effect of objectively monitored physical activity on physical functioning and onset of major mobility disability. Relatively small increases (> 48 minutes per week) in regular physical activity participation had significant and clinically meaningful effects on these outcomes.

**Trial registration:** ClinicalsTrials.gov NCT00116194

## Introduction

Physical inactivity is among the strongest predictors of physical disability in elders[1,2]. Furthermore, exercise prevents or improves conditions that underlie disability in older adults, including falls[3–6], hip fracture[7,8], cardiovascular disease[9,10], and diabetes[10–12]. Longitudinal studies suggest regular physical activity is associated with reduced mortality and risk of physical disability[13,14]. Several studies have demonstrated beneficial effects of physical activity programs on functional outcomes in older adults[15–19].

An important but understudied issue in physical activity trials in older adults is intervention adherence and the minimum dose necessary to achieve benefit. Previous physical activity intervention trials have reported that adherence among older adults is generally high (70–85%)[18,20,21]. However, many of these studies were of relatively short duration (6–18 months), and measured physical activity adherence by self-report or other indices of participation such as session attendance. To address gaps in knowledge regarding physical activity for disability prevention, the Lifestyle Interventions and Independence for Elders Study (the LIFE Study), a Phase 3 multicenter RCT compared a supervised moderate-intensity walking and resistance training physical activity program to a health education program on the incidence of major mobility disability was conducted[22]. The LIFE study demonstrated that a structured moderate intensity physical activity program reduced major mobility disability by 18% and persistent mobility disability by 28%, compared with a health education program[23].

In the present study, we examined whether differences in the dose of physical activity performed were associated with improvements in physical functioning and reductions in disability incidence. This analysis was an exploratory aim of the LIFE study. We also examined the stability/consistency of these results by examining the dose of physical activity using both objectively measured daily physical activity using accelerometry and self-reported minutes of walking plus weight training exercise.

## Materials and methods

**Fig 1:**
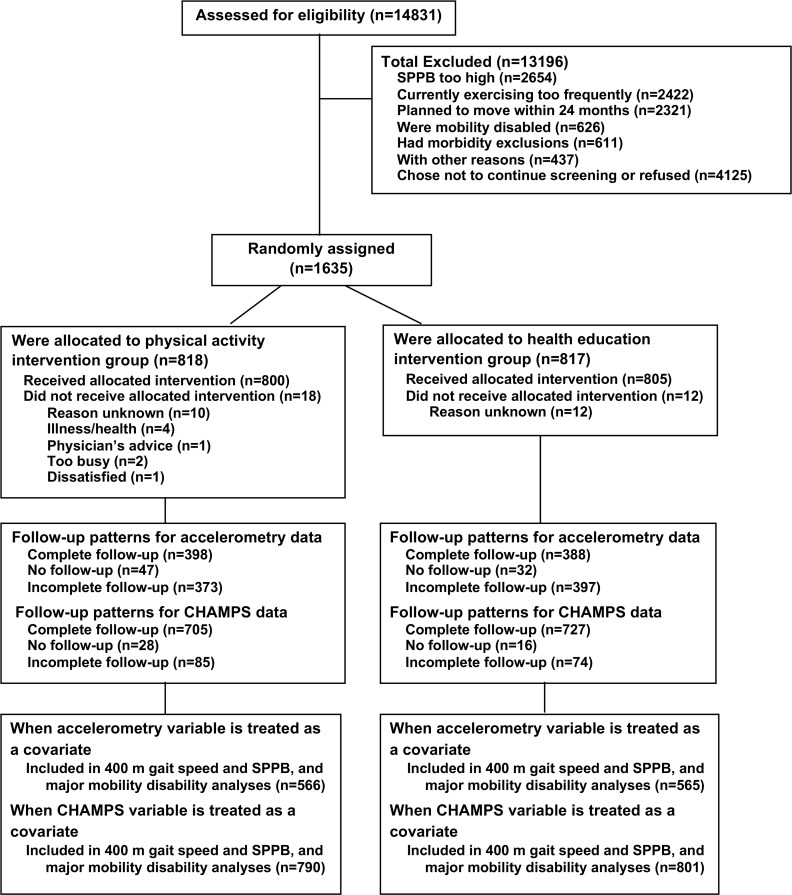
Flow of participants through the trial.

From February 2010 to December 2011, 14,831 participants were screened for the LIFE study at eight field centers; 1,635 of these participants were eligible and randomized (818 to physical activity and 817 to health education) (Fig 1).

### Eligibility

Comprehensive details of the LIFE study have been published previously[22,24]. Briefly, the LIFE study eligibility criteria were designed to target older persons (age 70–89 years) who were: a) inactive (reporting <20 min/week in the past month performing regular physical activity and <125 min/week of moderate physical activity); b) at high risk for mobility disability determined by a Short Physical Performance Battery (SPPB) score of 9 or lower[25,26]; c) able to walk 400 meters in <15 minutes; d) had no major cognitive impairment (Modified Mini-Mental State Examination [27](3MSE)) and e) able to safely participate in the intervention. This study was approved by the Institutional Review Board (IRB) at University of Florida, University of Pittsburgh, Pennington Biomedical Research Institute, Wake Forest University, Yale University, Stanford University, Northwestern University, and Tufts University and written informed consent was obtained from all participants.

### Study interventions

#### Physical activity intervention

The physical activity intervention included walking-based aerobic, resistance, flexibility, and balance training in a supervised class setting (2 x per week) with additional home-based physical activity goals[28]. The goal was to progressively move participants towards at least 150 minutes per week of moderate intensity physical activity, consistent with the 2008 Physical Activity Guidelines for Americans report[29].

Walking was the primary mode of physical activity, given its widespread popularity and ease of administration across a broad segment of the older adult population[30,31]. Participants also completed a 10-minute lower extremity resistance training program with ankle weights after walking exercise, a set of balance exercises, and a brief lower extremity stretching routine. Individualized home-based walking goals supplemented the supervised program.

#### Health education intervention

The health education intervention provided age-specific health information about “successful aging”. Health education consisted of workshops on topics relevant to older adults (e.g., negotiating the health care system, travel safety, nutrition, etc.). Each session included a brief program of seated upper extremity stretching/flexibility exercises. Health education classes met weekly for the first 26 and from week 27 on the program was offered two times per month and participants were prompted to attend at least once per month.

### Study assessments

Participants were evaluated at baseline, 6, 12, and 24 month clinic visits by staff masked to intervention arm allocation. Home, telephone, and proxy assessments were attempted if the participant could not come to the clinic. Since objective measures of physical activity were assessed through month 24 on all participants (i.e. minimum follow-up was 24 months), we have confined our analysis to this time frame.

#### Short Physical Performance Battery (SPPB)

The SPPB consists of a standing balance test, a usual pace 4 meter walk, and five timed repeated chair stands[32]. Each performance measure is assigned a categorical score ranging from 0 (inability to complete the test) to 4 (best performance possible) and a summary score ranging from 0 (worst performance) to 12 (best performance) was calculated.

#### 400 m walk test and assessment of major mobility disability

Participants were asked to walk 400 m at their usual pace, without overexertion, for 10 laps on a course defined by 2 cones placed 20 m apart. They could stop and rest for up to 1 minute for fatigue or related symptoms. Gait speed was calculated from the time to complete the walk and the distance covered.

Major mobility disability was defined as the inability to complete the 400 m walk test within 15 min without sitting and without the help of another person or walker[22]. Use of a cane was acceptable. When major mobility disability could not be objectively measured because the participant was not able to come to the clinic and lacked a suitable walking course at their home, institution or hospital, an alternative adjudication of the outcome was based on directly observed inability to walk 4 m in <10 sec, or self-, proxy-, or medical record-reported inability to walk across a room. If participants met these alternative criteria, it was considered that they would also not be able to complete the 400 m walk within 15 minutes.

#### Accelerometry

Participants were instructed to wear an accelerometer (Actigraph GT3X) on their hip for seven consecutive days except during sleep, showering/bathing, and swimming. Movement was captured along the vertical axis in 1-minute epochs, and non-wear time was defined as 90 minutes of consecutive zero counts[33]. An outlier minute was defined as having an activity count value that exceeded all of the participant’s non-outlier minutes and additionally the activity count value had to exceed the nearest value for the day by 1000 and exceed the median of the 2^nd^ highest daily value by 3500. Data were considered valid if apparent wearing evidence was ≥ 600 minutes per day for at least five days. Data reduction focused on technical and procedural errors as well as evident outliers. Ultimately, data from a total of 1,131 participants (566 from the physical activity intervention, 565 from the health education intervention) were used in this analysis. To be included in the analysis, they must have had a baseline measure and at least one of the follow-up measure.

Total physical activity was calculated as the average number of daily activity counts per minute. This measure correlates with energy expenditure assessed using oxygen consumption in older adults[34]. In the absence of well-accepted evidence-based accelerometry cut-points for physical activity intensity in older adults, a cut-point was set that demarcated time during activity (> 760 activity counts/minute)[35,36]. Data were expressed in min/week, and adjusted for wear time.

#### Self-reported physical activity

Participants completed a shortened version of the Community Health Activities Model Program for Seniors (CHAMPS-5) physical activity questionnaire[37]. The CHAMPS-5 assessed the average weekly minutes spent in walking and weight training from 5 specific items in this instrument related to these specific activities. These items queried frequency and duration of engagement in moderate to heavy strength training, light strength training, walking uphill or hiking up hill, walking fast or briskly for exercise, and walking leisurely for exercise or pleasure.

#### Hospitalizations

Because physical activity participation may be influenced by inter-current illness, we used reported hospitalizations as a measure of inter-current illness. At each contact, participants (or proxies if the participant was not available) were questioned about hospitalizations since the last visit. All records for hospitalizations were obtained and adjudicated independently by two experts who were blinded to randomization arm.

### Statistical analysis plan

The mean and standard deviation (SD) of change in physical activity from baseline by randomized arm at each visit were computed. The Wilcoxon rank sum test was used to compare the change in physical activity between randomization groups. Baseline characteristics, stratified by quartiles of change from baseline in accelerometer-determined physical activity at 24 months (interquartile ranges derived from physical activity arm) and by intervention arm, included means (± SD) for continuous variables or counts (%) for discrete variables. Analysis of variance was used to compare continuous characteristics across quartiles of change and a chi-square test was used to compare discrete characteristics across quartiles of change.

Physical activity measures, including minutes spent in activity associated with >760 activity counts/minute (by accelerometry) and CHAMPS-reported min/week in walking/weight training were the primary predictors of interest. They were categorized based on quartiles of changes at 24 months in the physical activity arm in the models. Baseline measures and changes to follow-up from baseline at 6, 12, and 24 months (treated as continuous measures or quartiles) were included in the analysis as an index of the dose of physical activity achieved. The outcomes included the SPPB, 400 m walk speed, and onset of major mobility disability. Analyses were initially restricted to the physical activity arm alone; however, we also examined these relationships using the same quartiles for both groups combined to examine changes in physical activity that occurred irrespective of intervention assignment. SPPB and 400 m walk speed were analyzed using mixed effects models with an unstructured parameterization for longitudinal covariance. For the analyses of the physical activity arm, models contained the following terms: field center and sex (both used to stratify randomization), baseline outcome measure, clinic visit, hospitalization, baseline physical activity measure, quartile of change in the physical activity measure, and quartile of change in the physical activity measure and clinic visit. For the entire sample analysis (both physical activity and health education), we additionally adjusted for randomization arm. Least squares means and their standard errors were presented from these models.

The effect of change in physical activity on time until major mobility disability was tested using a time-dependent Cox regression model, stratified by field center and sex. Failure time was measured from the time of randomization. Follow-up was censored at the first occurrence of loss-to-follow-up, death or the month 24 assessment. For participants who did not have any outcome assessments, a half hour of follow-up time was assigned, since we knew that they completed the 400 m walk at baseline. The models contained the following terms: baseline and change in physical activity measure, hospitalization, and randomization arm when the entire sample was used.

## Results and discussion

The change in accelerometer-determined activity and the change in self-reported walking plus weight training were significantly greater in the physical activity arm than health education at all time points (P < 0.001) (Table 1.). Baseline characteristics by quartile of accelerometer-determined physical activity are presented in Tables 2 and 3. Baseline differences in number of chronic conditions were observed in the PA arm and in gait speed in both arms across quartiles.

**Table 1:** Change in physical activity from baseline by randomized arm (differences between physical activity and health education P < 0.001 (calculated using the Wilcoxon rank sum test) between PA and HE at all time points.

	Change in minutes of physical activity (>760 activity counts/min)		
	6 months	12 months	24 months

Physical activity	31.3 ± 120.4^a^ N = 518	19.0 ± 122.7 N = 519	-8.9 ± 118.2 N = 466
Health education	-19.6 ± 139.3 N = 509	-25.7 ± 130.5 N = 511	-46.2 ± 132.2 N = 452
	Change in minutes of self-reported walking plus weight training minutes (from CHAMPS)		
Physical activity	151.6 ± 194.8 N = 774	141.9 ± 195.5 N = 761	133.7 ± 204.4 N = 724
Health education	29.1 ± 163.2 N = 796	36.8 ± 170.2 N = 777	29.4 ± 167.6 N = 737

^a^mean change ± standard deviation

**Table 2:** Baseline characteristics of the physical activity program participants by quartiles of change in accelerometer-determined physical activity (minutes per week above 760 counts per minute change between baseline and 24 months).

	Q1^a^ (n = 117)	Q2 (n = 116)	Q3 (n = 116)	Q4 (n = 117)	p-value
Age (years)	78.1 ± 5.1^b^	79.0 ± 5.1	79.6±5.7	77.9±5.3	0.06
Female No. (%)	80 (68.4%)^c^	76 (65.5%)	74 (63.8%)	75 (64.1%)	0.88
Race No. (%)					0.56
White	81 (69.2%)	92 (79.3%)	89 (76.7%)	92 (78.6%)	
African American	30 (25.6%)	18 (15.5%)	22 (19.0%)	21 (18.0%)	
Other	6 (5.1%)	6 (5.2%)	5 (4.3%)	4 (3.4%)	
No. of chronic conditions	1.8 ± 1.1	1.6 ± 1.1	2.0±1.3	1.6±1.1	0.05
Body mass index (kg/m^2^)	31.4 ± 6.4	29.7 ± 5.3	30.1±5.8	29.9±5.8	0.12
Short Physical Performance Battery (SPPB) score	7.6±1.5	7.5±1.5	7.3±1.7	7.5±1.7	0.49
400 m gait speed (m/sec)	0.84 ± 0.16	0.84 ± 0.16	0.80±0.15	0.87±0.18	0.01

^a^Q1: < -66; Q2: -66 ─ -8; Q3: -8–43; Q4: ≥ 43 (minutes per week above 760 counts per minute change between baseline and 24 months)

^b^Data are means ± standard deviations

^c^n (%).

**Table 3:** Baseline characteristics of the health education program participants by quartiles of change in physical activity by accelerometry (minutes per week above 760 counts/minute change between baseline and 24 months).

	Q1^a^ (n = 152)	Q2 (n = 134)	Q3 (n = 105)	Q4(n = 61)	
Age (years)	78.5 ± 5.3^b^	79.5 ± 5.3	79.5 ± 5.2	77.8 ± 5.3	0.07
Female	109 (71.7%)^c^	88 (65.7%)	71 (67.6%)	50 (82.0%)	0.12
Race					0.64
White	126 (82.9%)	102 (76.1%)	79 (75.2%)	51 (83.6%)	
African American	19 (12.5%)	24 (17.9%)	18 (17.1%)	8 (13.1%)	
Other	7 (4.6%)	8 (6.0%)	8 (7.6%)	2 (3.3%)	
No. of chronic conditions	1.7 ± 1.1	1.9 ± 1.1	1.8±1.1	1.7±1.1	0.18
Body mass index (kg/m^2^)	30.1 ± 5.8	30.7 ± 6.4	30.4 ± 6.0	30.2 ± 6.4	0.81
Short Physical Performance Battery score	7.6 ± 1.4	7.2 ± 1.6	7.4 ± 1.5	7.3 ± 1.8	0.26
400 m walking speed (m/sec)	0.86 ± 0.15	0.81 ± 0.16	0.82 ±0.18	0.83 ± 0.15	0.02

^a^Q1: < -66; Q2: -66 ─ -8; Q3: -8–43; Q4: ≥ 43 (minutes per week above 760 counts per minute change between baseline and 24 months)

^b^Data are means ± standard deviations

^c^n (%).

**Fig 2:**
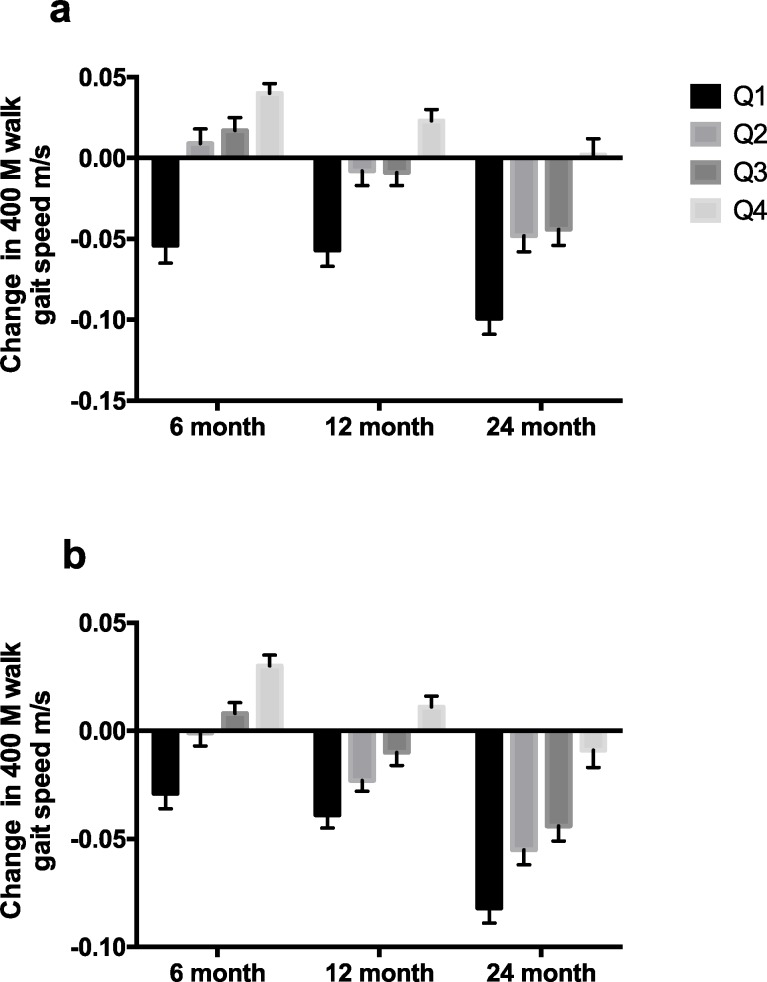
**Change in 400 m walk gait speed (m/s) compared to baseline gait speed at 6, 12, and 24 months according to quartiles of change physical activity by accelerometry from baseline to 24 months, (a.) physical activity arm alone and (b.) entire group combined (least square means ± SE).** Overall effect P < 0.0001. Effects within each time point P < 0.0001.

The mixed model analyses revealed statistically significant and clinically meaningful differences of the effect of quartile of change in accelerometry-based measures of change in activity from baseline to 24 month on the change in 400 m gait speed (Fig 2) for the physical activity arm over time (P < 0.001). The difference in gait speed change from lowest to the highest quartiles was between 0.05 and 0.10 m/s. This finding also held for the entire sample (Fig 2) (P < 0.001). These relationships remained when hospitalization was removed from the models (data not shown).

**Fig 3:**
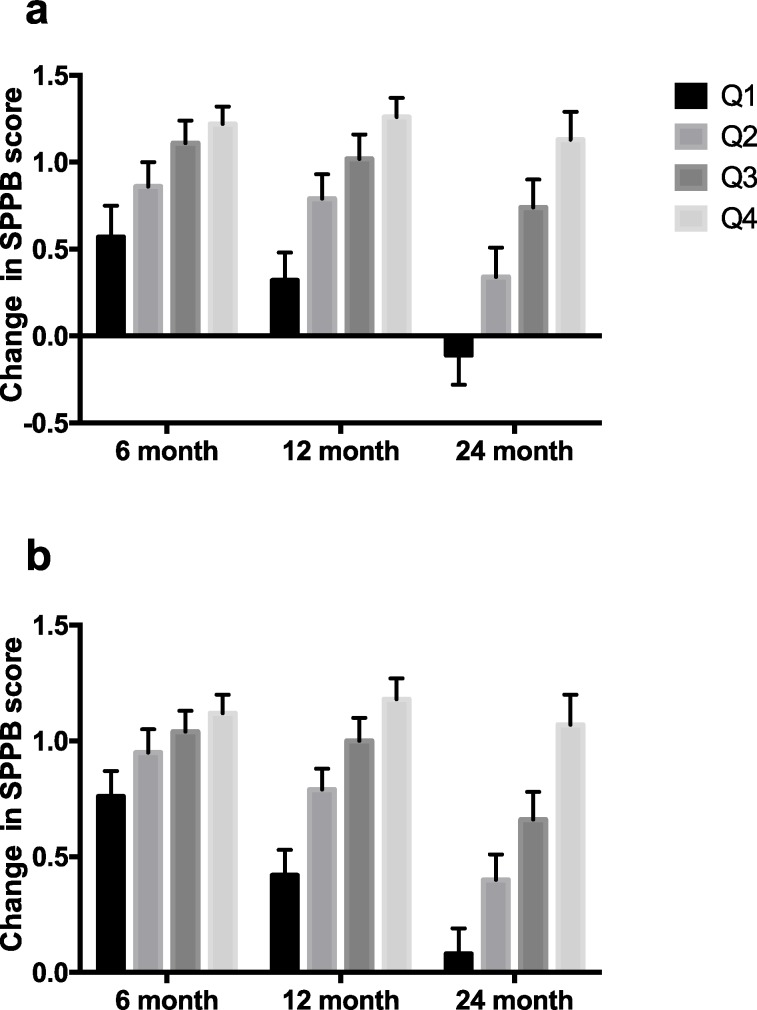
**Change in SPPB score compared to baseline SPPB score at 6, 12, and 24 months according to quartiles of change in physical activity by accelerometry from baseline to 24 months, (a.) physical activity arm alone and (b.) entire group combined (least square means ± SE).** Overall effect P < 0.0001. Effects within each time point all P < 0.01(physical activity arm); Effects within each time point all P < 0.06 (month 6, P = 0.06, months 12 and 24, P < 0.0001) (entire group).

The mixed model analyses also revealed a statistically significant and clinically meaningful difference of the effect of quartile of change in accelerometry-based measures of activity from baseline to 24 month in SPPB score for the physical activity arm over time (Fig 3A) (P < 0.001). The difference in SPPB change from lowest to the highest quartiles was between 0.5 and 1.5. Again, this held true when the entire sample was combined (Fig 3B) (P < 0.001). These relationships remained when hospitalization was not included in the models (data not shown).

The changes in physical activity by self-reported minutes of walking plus weight training from baseline to 24 months were also associated with significant changes in 400 m gait speed (P < 0.001) and SPPB scores (P < 0.001) (Table 4) in the physical activity arm and when the entire sample was combined (Table 5). Again, the results were stable when models were run excluding hospitalizations (data not shown).

**Table 4:** Association with change in gait speed and SPPB in physical activity arm participants, using quartile of change from baseline to 24 months of self-reported walking plus weight training.

Variable	visit	Q1^a^	Q2	Q3	Q4	p-value	Overall p-value
Change in 400 m gait speed	6	-.001±0.010	-.007±0.007	0.023±0.006	0.034±0.006	<0.001	<0.001
	12	-.045±0.010	-.024±0.007	0.003±0.006	0.012±0.006	<0.001	
	24	-.062±0.012	-.054±0.008	-.040±0.008	-.001±0.007	<0.001	
Change in SPPB	6	0.68±0.15	0.89±0.11	1.16±0.09	1.21±0.09	0.005	<0.001
	12	0.66±0.16	0.67±0.11	0.96±0.10	1.16±0.10	0.002	
	24	0.24±0.18	0.36±0.14	0.54±0.13	0.79±0.12	0.025	

^a^Q1: < 0; Q2: 0–105; Q3: 106–225; Q4: > 225 Self-reported time (minutes/week) walking and weight training; least square means ± standard errors.

**Table 5:** Association with change in 400 m gait speed and SPPB in the entire sample, using quartile of change of self-reported walking plus weight training from baseline to 24 months.

Variable	visit	Q1^a^	Q2	Q3	Q4	p-value	Overall p-value
Change in 400 m gait speed	6	-.011±0.005	-.004±0.004	0.011±0.004	0.016±0.004	<0.001	<0.001
	12	-.038±0.005	-.024±0.004	-.005±0.004	-.003±0.005	<0.001	
	24	-.069±0.007	-.055±0.005	-.044±0.006	-.024±0.007	<0.001	
Change in SPPB	6	0.64±0.09	0.90±0.07	0.99±0.08	1.11±0.08	0.002	<0.001
	12	0.61±0.10	0.71±0.07	0.92±0.08	0.98±0.09	0.004	
	24	0.12±0.11	0.49±0.08	0.54±0.10	0.74±0.10	<0.001	

^a^Q1: < 0; Q2: 0–105; Q3: 106–225; Q4: > 225 Self-reported time (minutes/week) walking and weight training; least square means ± standard errors.

We examined the relationship between quartiles of change in accelerometry-based activity and change in both self-reported walking plus weight training with the onset of major mobility disability for the physical activity arm and entire sample from baseline to 24 months. The relationships were statistically significant in the physical activity arm (P ≤ 0.003) and in the entire sample overall (P ≤ 0.001). For the physical activity arm alone, the onset of major mobility disability was reduced by 24% (P = 0.412), 22% (P = 0.489), and 77% (P = 0.001) in quartiles 2, 3, and 4 of change in accelerometry-based activity respectively and increased by 7% (P = 0.802), and reduced 57% (P = 0.007), and 75% (P < 0.001) in quartiles 2,3, and 4 of change in self-reported walking plus weight training respectively (Table 6). The hazard ratios were very similar for the entire sample (Table 7) and when hospitalization was removed as a covariate (data not shown).

**Table 6:** Hazard ratio for major mobility disability in the physical activity arm.

Variable	Quartile	HR (95% CI)^a^	p-value	Overall p-value (3df)
Quartile of change in physical activity by accelerometry	1	1.00		0.003
	2	0.76 (0.39, 1.48)	0.412	
	3	0.78 (0.40, 1.55)	0.484	
	4	0.23 (0.10, 0.52)	0.001	
Quartile of change in self-reported walking and weight training	1	1.00		<0.001
	2	1.07 (0.62, 1.84)	0.802	
	3	0.43 (0.23, 0.79)	0.007	
	4	0.25 (0.13, 0.49)	<0.001	

^a^Adjusted for baseline adherence measure, hospitalization, and quartile of adherence change.

**Table 7:** Hazard ratio for major mobility disability in the entire sample.

Variable	Quartile	HR (95% CI)^a^	p-value	Overall p-value (3df)
Quartile of change in physical activity by accelerometry	1	1.00		
	2	0.68 (0.43, 1.06)	0.091	<0.001
	3	0.65 (0.40, 1.06)	0.085	
	4	0.29 (0.16, 0.51)	<0.001	
Quartile of change of self-reported walking plus weight training	1	1.00		<0.001
	2	0.90 (0.64, 1.27)	0.558	
	3	0.57 (0.38, 0.85)	0.006	
	4	0.29 (0.18, 0.46)	<0.001	

^a^Adjusted for baseline adherence measure, randomization, hospitalization, and quartile of adherence change

The major results from this study were that changes in physical activity measured objectively by accelerometry as well as by self-report were associated with improvements in physical functioning over 24 months as well as a reduction in the onset of major mobility disability in a dose-dependent manner. These associations were stable when examined in the physical activity arm alone or when the physical activity and health education arms were combined. We observed similar results of physical activity participation on physical functioning and major mobility disability whether using accelerometry or self-report measures.

The magnitude of the increase in physical function in the physical activity arm was related to accelerometry-determined changes in physical activity and was progressively greater across all four quartiles of change. Observational studies have reported significant relationships between self-reported physical activity measures [38–41] and objective measures of physical functioning in older adults[42–44]. McDermott et al. reported that the decline in physical performance in patients with peripheral artery disease is slower in individuals who report walking 3 or more times per week, compared to those who walk for exercise less frequently or not at all[45]. In contrast to these observational studies, results reported here are from a randomized controlled trial and show greater improvements in physical functioning in individuals who participate in more physical activity. The difference in gait speed change from lowest to the highest quartiles was between 0.05 to 0.10 m/s, a magnitude which has been associated with reductions in mortality risk [46] and patient-reported outcomes[47]. The difference in SPPB score change from lowest to highest quartiles was between 0.5 and 1.0 and is within the range of a clinically meaningful difference[48].

Greater differences in both the change in self-reported physical activity and objectively measured activity were related to prevention of major mobility disability. The present analysis demonstrated a robust decline in major mobility disability in the highest quartile of change in accelerometer-determined physical activity. The change in physical activity observed in the highest quartile translated to approximately 43 minutes per week. This level of physical activity is approximately the dose of a single LIFE study physical activity session[28]. These data suggest that an increase in the dose of physical activity equivalent to a single LIFE physical activity session per week was sufficient to dramatically reduce disability risk and result in meaningful improvements in physical functioning.

The dose effect of physical activity on physical function and major mobility disability was also observed when we combined data from both study arms. This finding highlights that participants randomized to physical activity and whose adherence to the intervention was relatively low achieved smaller benefits, however, any participant randomized to health education who spontaneously increased their physical activity achieved significant and meaningful improvements in their function and disability risk. These effects were observed even when accounting for hospitalizations during the trial to adjust for the burden of inter-current illness.

The strengths of this investigation are the objective measurement of physical activity, the substantially greater length of the follow-up than in prior studies, and the study population, which consisted of individuals at high risk for mobility disability. Furthermore, the results of our analysis when both intervention arms were combined indicate that if participants increased or decreased their levels of activity over the course of the trial, irrespective of group assignment, their change in physical functioning or mobility disability was related to this change in activity. In addition to these strengths, there are several study limitations. Among them are the fact that the LIFE Study was not designed to specifically address varying doses of physical activity on physical functioning and disability risk. Differences in health status and inter-current illness may influence physical activity behavior and therefore may be potential confounders in these analyses. In addition, since both independent and dependent variables were assessed over the same time intervals it is not possible to establish temporal precedence. However, our models were stable even when inter-current hospitalizations were included in the analyses.

## Conclusion

In conclusion, greater changes in physical activity behavior measured objectively by accelerometry and self-report were associated with clinically meaningful improvements in physical functioning and reductions in the development of major mobility disability. The dose of change in physical activity associated with the greatest benefit was greater than 48 minutes per week of physical activity. These data have important public health implications for the benefits of physical activity in mobility-limited older adults. These data support that beneficial effects of physical activity can be realized with substantially less physical activity than is currently recommended for most inactive older adults.

## Supporting information

S1 Checklist CONSORT checklist. (DOC)

S1 Appendix LIFE study protocol. (PDF)
