# Supplementary material for: Dose of physical activity, physical functioning and disability risk in mobility-limited older adults: Results from the LIFE study randomized trial
Source: PLoS One. 2017 Aug 18;12(8):e0182155. doi: 10.1371/journal.pone.0182155 (PMC5562326; doi:10.1371/journal.pone.0182155)
Supplement: S1 Appendix — (PDF) [file pone.0182155.s002.pdf]

Lifestyle Interventions and Independence for Elders

The LIFE Study

NIH U01AG022376

Protocol

Table of Contents

Page

1. Summary **6**

2. Background and Rationale **7**
- 2.1. General Overview
- 2.2. Causes of Physical Disability in Older Persons
- 2.3. Health Benefits of Physical activity on Chronic Disease and Disability
- 2.4. Report of the Surgeon General on Physical Activity and Health – Gaps in Evidence
- 2.5. Need for a Definitive Trial
- 2.6. LIFE-Pilot study

3. Overview of Trial Design **11**
- 3.1. Design
  - 3.1.1. Primary Hypothesis and Primary Outcome
  - 3.1.2. Secondary and Tertiary Hypotheses
  - 3.1.3. Interventions
  - 3.1.4. Study Population
- 3.2. Intentionally Blank
- 3.3. Sample Size Justification

4. Study Population **18**
- 4.1. Eligibility Criteria
  - 4.1.1. Targeting Populations at High Risk for Disability
  - 4.1.2. Establishing Eligibility
- 4.2. Exclusion Criteria
  - 4.2.1. Exclusion Criteria for Behaviors that Preclude Successful Participation in the Trial
  - 4.2.2. Exclusion Criteria for Underlying Diseases Likely to Limit Lifespan and/or Effect the Safety of Participants
  - 4.2.3. Temporary Exclusion Criteria

5. Recruitment and Retention **22**
- 5.1. Recruitment
  - 5.1.1. Screening Process
- 5.2. Retention and Drop-out Recovery
  - 5.2.1. Identifying Secondary/Proxy Contacts
  - 5.2.2. Retention Promotion Efforts
  - 5.2.3. Drop-out Recovery Efforts
- 5.3. Monitoring Recruitment and Retention
  - 5.3.1. Retention and Efforts to Maintain Contact with Inactive Participants
  - 5.3.2. Monitoring and Quality Control of Recruitment and Retention

6. Measures and Procedures **26**
6.1. Informed Consent
6.2. Measures
6.2.1. 400 Meter Walk Test
6.2.2. Short Physical Performance Battery (SPPB or EPESE Battery)
6.2.3. The Mobility Assessment Tool (short form): MAT-sf
6.2.4. Hand Grip Strength
6.2.5. Self-Reported Physical Function, Activity and Disability – Accelerometry
6.2.6. Process Measures
6.2.7. Vital Signs
6.2.8. Medication Inventory
6.2.9. ECG
6.2.10. Social, Economic and Health Related Questionnaires
6.2.11. Cognition
6.2.12. Health-Related Quality of Life
6.2.13. Quality of Well Being
6.2.14. Health Care Utilization
6.2.15. Biological Specimen Sampling and Storage
6.2.16. Sleep-Wake Disturbances
6.2.17. Pulmonary Questionnaires
6.2.18. Ventilatory Capacity
6.2.19. Cardiovascular questionnaires
6.2.20. Ankle-Brachial Index (ABI)
6.2.21. Social Cohesion and Trust Scale and Environmental Walkability Scale (NEWS-A)
6.2.22. Cancer Follow-up Form
6.2.23. Cardiovascular Medical History Baseline Update Form
6.2.24. Extension Study Participant Interest Survey
6.3. Blank
6.4. Randomization
6.4.1. Final Eligibility Assessment
6.4.2. Randomization Scheme
6.4.3. Masking or Blinding

7. Interventions **37**
7.1. Intervention Theory and Goals
7.1.1. Intervention Theory
7.1.2. Goals of the Intervention Arms
7.2. Physical Activity Intervention
7.2.1. Contact Mode and Frequency
7.2.2. Participant Assessment at Baseline
7.2.3. Intensive Contact Phase
7.2.4. Adoption phase
7.2.5. Maintenance Phase
7.2.6. Educational Modules
7.3. Successful Aging Program Intervention
7.3.1. Contact Mode and Frequency
7.3.2 General Content and Structure of Intervention Modules
7.4. Strategies for Keeping Participants Involved in the Intervention

7.4.1. Adherence and Monitoring
7.4.2. Strategies For Promoting Adherence in the Physical activity Group
7.4.3. Strategies to Enhance Participation Rates in the Successful Aging Program
7.4.4. Protocol for Managing Illness/Injury and Other Health Problems

8. Participant Safety and Confidentiality **44**
8.1. Data and Safety Monitoring Board
8.2. Medical Problems Detected During the Study Assessments
8.3. Safety Considerations for Study Assessments
8.4. Safety Considerations for Physical Activity Intervention
8.4.1. Pre-Physical Activity Safety Screening
8.4.2. Safety Measures during Physical Activity
8.5. Adverse Events
8.6. Confidentiality

9. Feasibility Evaluation and Stopping Rules **53**
9.1. Feasibility Evaluation of Definitive Trial
9.2. Stopping Based on Safety Concerns
9.3 Monitoring the Primary Outcome

10. Assessment Schedule and Outcomes Ascertainment **54**
10.1. Summary of Baseline and Follow-Up Assessments
10.2. Primary Outcome Measure: Major Mobility Disability
10.2.1. Time Frame for Follow-Up Assessments of the Major Mobility Disability Outcome
10.3. Secondary and Tertiary Outcome Measures
10.3.1. Cognition
10.3.2. Serious Fall Injuries
10.3.3. Persistent Mobility Disability
10.3.4. Self-Reported Disability
10.3.5. Cost Effectiveness
10.3.6. Tertiary Outcomes
10.4. Outcome Adjudication Procedures
10.4.1. Adjudication of the Major Mobility Disability Outcome
10.4.2. Death
10.4.3. Fractures
10.4.4. Pulmonary and cardiovascular outcome adjudication
10.5. Ancillary Studies
10.6. Tracking Health Care Utilization and Vital Status and HIPAA Compliance

11. Data management and Quality Control **63**
11.1. Data Management
11.1.1. Field Centers-Screening, Randomization, and Follow-Up Visits
11.1.2. Data Entry, Verification and Quality Control
11.1.3. Intentionally Blank
11.1.4. Database Closure
11.2. Management of Administrative Data
11.3. Quality Control (QC)

12. Statistical Considerations **65**

12.1. Introduction and Aims
12.2. Analysis Plans

13. Trial Organization **69**

14. Study Timeline **73**

15. Participating Sites **74**

16. Bibliography **75**

Appendix A Diagnostic protocol for mild cognitive impairment (MCI) and dementia **92**

Appendix B LIFE Stopping Guidelines **96**

1. Summary

As life expectancy in the United States continues to rise, the maintenance of physical independence among older Americans has emerged as a major clinical and public health priority. Efficient and reliable locomotion, or the ability to move without assistance, is a fundamental feature of human functioning. Older people who lose mobility are less likely to remain in the community, have higher rates of morbidity, mortality, and hospitalizations and experience a poorer quality of life. Several studies have shown that regular physical activity improves physical performance, but definitive evidence showing that mobility disability can be prevented is lacking. A Phase 3 randomized controlled trial is needed to fill this evidence gap.

The LIFE Study is a Phase 3, multicenter randomized controlled trial (RCT) designed to compare a moderate-intensity physical activity program to a successful aging health education program in 1,600 sedentary older persons who are followed for an average of 2.7 years. The primary outcome is major mobility disability, defined as inability to walk 400 m. Secondary outcomes include cognitive function based on the Digit Symbol Substitution Test (DSST) and the Hopkins Verbal Learning Test (HVLT); serious fall injuries; persistent mobility disability; the combined outcome of major mobility disability or death; disability in activities of daily living; and cost-effectiveness. Tertiary outcomes include the combined outcome of mild cognitive impairment or dementia, a composite measure of the cognitive assessment battery, physical performance within pre-specified subgroups defined on the basis of race, gender and baseline physical performance, sleep-wake disturbances, dyspnea, ventilatory capacity, pulmonary events, and cardiovascular events.

The physical activity intervention consists primarily of walking at moderate intensity, lower extremity resistance exercises, balance exercises, stretching and behavioral counseling. The successful aging intervention consists of health education seminars regarding health-related matters and upper extremity stretching exercises.

This trial will provide definitive evidence regarding whether physical activity is effective and practical for preventing major mobility disability. These results will have crucial implications for public health prevention in a rapidly aging society, and will fill an important gap in knowledge for practicing evidence-based geriatric medicine. The study will also yield valuable information concerning the efficacy and effectiveness of physical activity across a broad spectrum of important health outcomes. The study will impact both clinical practice and public health policy, and will, therefore, benefit individuals and society.

The Coordinating Center is at the University of Florida and the Data Management Analysis and Quality Control Center (DMAQC) is at Wake Forest University School of Medicine. The 8 field sites participating in the LIFE Study are University of Florida, Gainesville, FL; Northwestern University, Chicago, IL; Pennington Biomedical Research Center, Baton Rouge, LA; University of Pittsburgh, Pittsburgh, PA; Stanford University, Palo Alto, CA; Tufts University, Boston, MA; Wake Forest University, Winston Salem, NC; and Yale University, New Haven, CT.

2. Background and Rationale

2.1. General overview

The life expectancy of older Americans continues to increase, with persons aged ≥70 yrs representing the fastest growing segment of the US population.[1] While prolongation of life remains an important public health goal, of even greater significance is that extended life should involve preservation of the capacity to live independently and to function well.[2] Therefore, identification of **proven interventions to prevent disability** is a major public health challenge.[3] Mobility and activities of daily living (ADL) represent tasks that are necessary for the maintenance of basic independent functioning.[4, 5] The inability to perform these activities marks a serious decline in functional health, conferring increased risk of institutionalization and death.[6, 7]

Most older adults are sedentary.[8, 9] Among this latter population, many are mobile and free of disability, but are at high risk for loss of mobility, which, in turn, is a key predictor of further decline and of increased risk of mortality. It is these individuals who would represent the target population for the LIFE intervention.[10-12]

2.2. Causes of Physical Disability in Older Persons

In most cases, physical disability is directly caused or aggravated by acute events (stroke and hip fracture) and chronic conditions (heart failure, coronary heart disease, diabetes and arthritis).[13, 14] In contrast, some individuals with no clear connections to a single disease experience progressive decline in physical function, with subsequent development of age-related physical disability. As diverse as the etiologies of physical disability are, sarcopenia (a progressive loss in skeletal muscle mass and strength) is hypothesized to represent a common pathway that is associated with the initial onset and progression of physical disability in many individuals.[15] Low levels of cardiorespiratory fitness also contribute to functional limitations.[16, 17]

2.3. Health Benefits of Physical activity on Chronic Disease and Disability

Exercise may benefit many morbid conditions that underlie disability, including risk of falls,[18-21] hip fracture,[22, 23]cardiovascular disease,[24, 25] respiratory diseases,[26] cancer,[27] diabetes,[25, 28, 29] osteoporosis,[30-32] low fitness and obesity,[33, 34] sleep-wake disturbances.[35] dyspnea and ventilatory capacity.[36-38] **Physical inactivity** is one of the strongest predictors of physical disability in elders.[39-41] In longitudinal studies, regular physical activity is not only associated with extended longevity, but also is associated with reduced risk of physical disability, including disability in activities of daily living.[8, 42-46] Of the 6,200 older persons free of baseline disability in the EPESE studies, those with a low level (lowest tertile) of regular physical activity were 1.8 times more likely to develop ADL[4] or mobility disability over 4 yrs than those with a high level (upper tertile) of physical activity.[8, 47] The benefit of exercise on physical function may be mediated by the prevention of frequently disabling conditions, such as serious fall injuries, or by a direct effect on impairments, such as reduced muscle strength,[48, 49] low cardiorespiratory fitness[17] and impaired balance.[50] In a cohort of older Finnish men and women, those involved in a high level of everyday physical activity (household chores, walking and gardening) showed significantly less decline in knee extension strength and grip strength after 5 years, as compared to those who were sedentary.[48]

Several studies have demonstrated the beneficial effects of physical activity programs on functional outcomes in older adults.[51-54] In FAST,[52] a RCT conducted at WFUHS and UT Memphis among 439 community dwelling older adults with knee osteoarthritis, self-reported physical function was significantly improved, as evidenced by reductions in disability in activities of daily living,[55] among those participating in an 18-

month aerobic exercise training or resistance exercise training program, as opposed to those participating in a health education program. The FAST exercise programs also significantly improved objective physical performance, walking speed and postural sway (balance).[56] In other studies, patients with chronic obstructive pulmonary disease[57] or heart failure[58, 59] improved physical function and distance walked in 6 min after a physical activity program. Older adults experienced beneficial physical health effects from structured exercise programs, and a strengthening exercise program among frail older persons significantly improved functional mobility, gait speed and muscle strength.[60, 61]

In healthy older adults, the beneficial physiological effects of a structured exercise program have been conclusively demonstrated. Regular exercise that emphasizes aerobic conditioning and/or strength training increases aerobic capacity,[62, 63] muscle strength[64-69] and endurance.[66] Despite these findings, it remains unclear whether the positive effects of exercise interventions can be sustained for a sufficient duration of time and maintained at adequate intensity to prevent a **clinically significant disability outcome**, thereby **prolonging independence**. Addressing this question requires new data from an intervention study with a sufficiently large sample size, a long follow-up time and appropriate disability outcome measures.

In addition to activities of daily living (as discussed above), exercise may benefit a number of other secondary outcomes addressed in LIFE. Of particular importance are cognitive decline and serious fall injuries. Recent evidence supports the likelihood that exercise can have a beneficial effect on the brain and on cognitive functioning. In animal models, exercise increases levels of brain-derived neurotrophic factor and other growth factors, stimulates neurogenesis, increases resistance to brain insult and promotes gene expression that may benefit brain plasticity processes.[70] A large observational study in physically capable older men showed that low levels of walking were predictive of higher rates of dementia[71] and an RCT demonstrated selective improvement in executive control processes[72] after 6 months of aerobic exercise. Further work is needed, however, to clarify the effect of longer-term exercise on early cognitive decline.

Several exercise-based interventions have demonstrated a reduction in falls,[18, 20, 73] nonetheless, none has had sufficient power to show a reduction in serious fall injuries such as fractures.[18, 20, 74, 75] Because the morbidity, costs, and mortality associated with serious fall injuries are immense,[76] the public health relevance of our physical activity intervention will be enhanced if we can demonstrate a reduction in serious fall injuries, in addition to a reduction in major mobility disability.

2.4. Report of the Surgeon General on Physical Activity and Health – Gaps in Evidence

The Surgeon General's report on physical activity and health emphasizes the importance of exercise at all ages and documents the **wide range of health benefits that result from exercise**.[77] The report stresses that moderate intensity exercise such as walking can be quite effective in improving health, and it recommends 30 min of this activity on most, if not all, days of the week. The report suggests that to attain these benefits, endurance exercises should be supplemented with strength-developing exercises at least twice/week. Similar recommendations emerged from the recent U.S. Department of Health and Human Services Physical Activity Guidelines for Americans (http://www.health.gov/paguidelines/guidelines/default.aspx). That report called for a program of aerobic and strengthening activities in older adults similar to younger adults but modified if necessary for certain chronic conditions, with addition of balance exercises when appropriate.

Consistent with the findings presented in the reviews noted above, the reports points to "promising evidence" that **exercise in older adults may preserve the ability to**

maintain independent living and reduce the risk of falling. While this statement relies on clinical trial evidence regarding the effects of exercise on impairments such as decreased strength and balance, evidence supporting the beneficial effects of exercise on **maintenance of independence** is entirely based on **observational studies**. These latter studies are especially prone to bias, since healthier older people are much more likely to be physically active, and statistical adjustment for level of comorbidity in observational studies is never fully adequate. The aforementioned studies do not comprehensively assess disease status and almost none can adjust for the severity of all diseases that may be present. Thus, residual confounding is highly likely in even the best-conducted observational studies, and the corresponding data **cannot provide definitive evidence regarding whether exercise can prevent the onset of disability in older people**. Furthermore, in persons who already have impairments and functional limitations (and who reflect the target population for the LIFE intervention), diseases causing these impairments could lead to eventual disability, even if these impairments improve with exercise. It is therefore critical that a RCT be conducted to evaluate the Surgeon General report's proposed benefit of exercise in preventing disability. In addition, findings that a large subset of RCT participants especially vulnerable to disability could be identified and successfully targeted for disability prevention would provide important information to supplement the current Surgeon General's recommendations. One year after release of the report, less than half of older Americans had awareness of the report, with substantially lower rates in individuals with lower educational levels.[78] **A RCT demonstrating that exercise prevents disability would be expected to substantially increase public awareness of the benefits of exercise in older persons.**

2.5. Need for a Definitive Trial

Since much is known about physical activity and functional outcomes, why is a new, large clinical trial needed? The benefits of physical activity have been demonstrated only in the context of change in intermediate measures such as disability scales, balance and performance scores, or muscle strength.[52] There are no proven interventions to **prevent the onset** of major mobility disability in older persons who are initially non-disabled. SHEP demonstrated the benefits of antihypertensive treatment of cardiovascular events and death,[79] but not on ADL disability.[80, 81] Results from growth hormone trials were also disappointing.[82] Physical activity represents an extremely promising intervention; yet **evidence for prevention of mobility and ADL disability remains inconclusive**,[83] and is derived only from secondary data analyses.[55]

Several examples from large RCTs demonstrate the problems of relying exclusively on **intermediate or surrogate outcomes** and on **observational** data. These studies include the pharmacological treatment of arrhythmias (CAST)[84] and hypertension (ALLHAT),[85] as well as others focusing on hormone replacement therapy (HERS, WHI).[86, 87] Findings from these studies suggest that results from trials using surrogate outcomes may not always apply to generalized prevention of events. This concept applies equally to disability studies in older persons. Improvement in intermediate measures does not guarantee that underlying disease processes and physiologic decrements associated with aging won't ultimately cause disability, even when intermediate measures respond to interventions such as exercise. Furthermore, concerns remain regarding whether physical activity in elders can be sustained for a sufficient **duration of time** and maintained at adequate **intensity** to actually improve clinically significant outcomes over the long term. In addition, physical activity interventions in functionally impaired older persons may have **adverse consequences**. Based on the above considerations, **conclusive evidence is lacking** concerning whether exercise interventions can reduce the risk of onset of major mobility disability over the long term in the general older population, and whether the

benefits outweigh the potential risks. By providing a definitive answer regarding whether or not physical activity is effective and practical for preventing major mobility disability, as operationalized by the inability to walk 400 m, the results of the LIFE Study will have crucial implications for **public health prevention** in a rapidly aging society, and will fill an important gap in knowledge for practicing **evidence-based geriatric medicine**.

2.6. LIFE-Pilot study

The LIFE-P pilot study was conducted to refine key trial design benchmarks, including the primary outcome of major mobility disability, sample size calculations, methods for recruitment, retention, adherence to and safety of the interventions, and organizational infrastructure.[88, 89] A total of 424 sedentary older persons were randomized to a physical activity intervention or a successful aging health education intervention and were followed for at least one year at four sites. All pilot study goals were successfully achieved. Compared to the successful aging health education group, the physical activity group had a lower incidence of major mobility disability defined as inability to walk 400 m (Figure). The pilot study provides promising preliminary evidence that physical activity may prevent major mobility disability. However, a larger and longer-term randomized controlled trial is needed on this important topic.

Pilot study: cumulative hazard of time until major mobility disability according to randomized groups

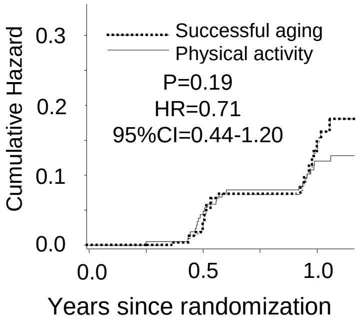

3. Overview of Trial Design

3.1. Design

The LIFE Study is a **multicenter single-blind RCT** involving physical activity vs. a successful aging program, with an average follow-up of 2.7 years (range 1.9-3.5 years or 23-42 months) in 1600 non-disabled, community-dwelling persons age 70-89 years across 8 Field Centers.

The **inclusion criteria** are (1) age 70 to 89 years; (2) summary score <10 on the EPESE short physical performance battery (SPPB);[90] (3) sedentary lifestyle; (4) ability to complete the 400 m walk test within 15 minutes without sitting or the help of another person, or the use of a walker; and (5) willingness to be randomized to either intervention group. The **exclusion criteria** reflect conditions that may interfere with the conduct of the physical activity program. LIFE plans to recruit 65% women and 22.5% minorities.

3.1.1. Primary Hypothesis and Primary Outcome

Primary hypothesis: Compared with a successful aging (SA) health education program, a **long-term** structured physical activity (PA) program reduces the risk of major mobility disability, defined as incapacity to walk 400 m.

After a thorough evaluation of possible alternative approaches, LIFE has selected as the primary outcome for the full-scale trial **time to the onset of major mobility disability**. This outcome is adjudicated as described below. The objective component of the **major mobility disability** outcome is defined as the inability to complete a 400 m walk test within 15 minutes without sitting or the help of another person. Individuals who complete the walk in more than 15 minutes have an extremely slow pace (<0.45 m/sec), which would make their walking capacity of little utility in daily life.[91] Selecting a higher cut point, such as 30 or 60 minutes, makes the objective assessment impractical and does not add to the clinical significance of the outcome. Major mobility disability is assessed every six months by staff who are **blinded** to the intervention assignment.

3.1.2. Secondary and Tertiary Hypotheses

Secondary hypotheses:

Compared with random assignment to a successful aging program, random assignment to a long-term structured physical activity program:

- Improves pre-specified measures of cognitive function based on the Digit Symbol Test (DSST) and the Hopkins Verbal Learning Test (HVLT);
- Reduces the risk of serious fall injuries;
- Reduces the risk of persistent major mobility disability and of the combined outcome of major mobility disability or death;
- Reduces the risk of disability in activities of daily living (ADLs); and
- Is cost-effective.

Tertiary hypotheses: Compared with SA, the PA program

- Reduces the risk of the combined outcome of all-cause mild cognitive impairment or dementia (MCI/D);
- Improves performance on a composite measure of cognitive function;
- Improves physical performance within subgroups defined on the basis of ethnicity/race, gender and baseline performance;
- Results in improvements in measures of physical performance and walking speed that vary with the amount of intervention received;
- Improves sleep-wake disturbances and leads to a lower incidence of sleep-wake disturbances;
- Reduces dyspnea and improves ventilatory capacity;
- Reduces the risk of hospital admissions for exacerbation of airway disease (asthma, bronchitis, emphysema, &/or COPD) or pneumonia; and

- Reduces the risk of combined cardiovascular (CVD) events including:
  - Myocardial infarction (MI),
  - Angina requiring hospitalization,
  - Any stroke (ischemic or hemorrhagic),
  - Transitory ischemic attack (TIA) requiring hospitalization,
  - Hospitalization for carotid artery disease,
  - Hospitalization for congestive heart failure (CHF),
  - Hospitalization for peripheral artery disease (PAD) or outpatient revascularization for PAD,
  - Ruptured abdominal aortic aneurism (AAA), and
  - CVD death.

3.1.3. Interventions

The physical activity program includes aerobic, strength, flexibility, and balance training. LIFE focuses on **walking** as the primary mode of physical activity for preventing/postponing the combined outcome of major mobility disability or death, given its widespread popularity and ease of administration across a broad segment of the older adult population.[77, 92] Other forms of endurance activity (e.g., stationary cycling) are, however, utilized when regular walking is contraindicated medically or behaviorally. Each session is preceded by a brief warm-up and followed by a brief cool-down period. In light of current clinical guidelines, participants are instructed to complete **flexibility** physical activities following each bout of walking. Moreover, participants are instructed to complete a 10-minute routine that focuses primarily on **strengthening exercises.** As has been done in other strengthening programs for older adults,[34, 93] supplementary instructional materials (e.g., videotapes, printed materials) are supplied to participants in this group, to reinforce the strength training occurring during setting-based instruction, so that it can be generalized to the home environment. **Balance training**[52] is introduced as a complement to the endurance and strength components. In addition, the intervention encourages participants to increase all forms of physical activity throughout the day, such as leisure sports, gardening, use of stairs as opposed to escalators, and leisurely walks with friends.

Intensity of training. Participants are introduced to the intervention exercises in a structured way such that they begin with **lighter intensity and gradually increase** over the first 2-3 weeks of the intervention. LIFE promotes walking for physical activity at a **moderate intensity**, and relies on **ratings of perceived exertion** as a method to regulate physical activity intensity.[94, 95] Using Borg's scale,[96] that ranges from 6 to 20, participants are asked to walk at an intensity of 13 (activity perception SOMEWHAT HARD). They are discouraged from exercising at levels that approach or exceed 15 (HARD) or drop to a rating of 11 (FAIRLY LIGHT) or below.

The intervention consists of a general weekly walking goal of 150 minutes. This is consistent with the public health message from the Surgeon General's report that moderate physical activity should be performed for 30 minutes on most if not all days of the week (150-210 total minutes). This goal is **approached in a progressive manner** over the course of the trial. There are multiple ways that the goal can be achieved, based on the physical abilities and constraints of each participant.[52, 97] In light of the heterogeneity of the target population (with respect to physical capabilities and health status), this study allows to more specifically define the variability in participants' ability to reach this weekly target, to estimate the dose-response relationship between incremental increases in weekly physical activity and to better specify the level of ongoing behavioral instruction needed to achieve such changes.

3.1.4. Study Population

LIFE plans to recruit sedentary and physically impaired, but ambulatory, community living older persons age 70 to 89 years. The specific inclusion and exclusion criteria are summarized below. These criteria are intended to select a population that is at higher risk of experiencing the major mobility disability outcome, would most likely benefit from the physical activity intervention, and would most likely comply with the intervention and assessment protocols. This age group is selected because it is at high risk of major mobility disability,[90] and it may have a sufficiently long life expectancy[1] to participate in a trial that lasts up to 3.5 years.

3.2. Intentionally Blank

3.3. Sample Size Considerations

The LIFE trial has targeted the recruitment of **1600 participants** across 8 Field Centers.

The **primary aim** of the LIFE Study is to assess the long-term (average 2.7 yrs) relative effect of randomization of sedentary persons aged 70 to 89 years to a physical activity intervention on the **primary outcome** of time until major mobility disability defined as inability to walk 400 m.

The primary study hypothesis of LIFE will be tested based on a two-tailed significance level of 0.05. In this analysis, the "intention to treat" approach will be used in which participants are grouped according to randomization assignment.

The main comparisons of intervention groups with respect to the distribution of time until the first post-randomization occurrence of a primary outcome will be based on survival analyses. To compare intervention arms, we will use a likelihood ratio test from a Cox regression model, stratified by field center and gender. Failure time is measured from the time of randomization.

Incidence of the Primary Outcome: In the LIFE Pilot study[88] (LIFE-P), after exclusion of LIFE-P participants reporting >125 min of moderate level exercise at baseline, we estimated the 1-year failure rate in the SA arm to be 18% (Kaplan-Meier estimate). LIFE-P was not designed to estimate whether this rate increases in future years of follow-up. However, from Health ABC data we project the annual incidence rate may increase by as much as 29% in years 2-4 in participants with characteristics similar to LIFE. Using this, we have conservatively incorporated a 15% increase in the average annual incidence rate in power calculations. Thus, we adopt assumptions of an initial annual incidence rate of **18%** and project that this rate will increase, on average, to **21%** after two years of follow-up.

Effect Size: We have calculated the power for effect sizes ranging from 20% to 25% relative effects. While too small to estimate a relative effective size with precision, LIFE-P observed a 29% (hazard ratio=0.71; 95% CI [0.44,1.20]) relative reduction in this primary outcome. A sensitivity analysis described below was performed to examine how varying levels in the rates of drop-ins, drop-outs, and non-adherence affect our relative effect sizes.

Average Follow-up: We assume that recruitment will take 21 months. Power is calculated under two scenarios: 1) an assumption of a uniform recruitment rate; and 2) that the recruitment rate will accelerate during these 21 months so that approximately **50%** of participants will be recruited in the first 12 months, and **50%** will be recruited in the final **9** months. Close-out visits will occur for 3 consecutive months. The first participant recruited will have a close-out visit at 42 months (3.5 years) of follow-up and the final randomized participant will be followed for 23 months (1.9 years). Using these projected recruitment rates and lengths of follow-up, the average follow-up time will be approximately 31 months.

Observational Follow-up: Following the completion of the study intervention period, all participants will be asked to complete an additional assessment visit approximately one year after their last assessment visit to gather additional safety data and confirm the occurrence of the persistent mobility disability outcome. This visit will be referred as the Post Intervention Visit (PIV).

Loss to Follow-up: In LIFE-P,[88] 400 m walk results or definitive adjudications were obtained in 94% (6 months) and 92% (12 months) of participants. We will assume that **loss to follow-up** accumulates at **8%/yr** throughout LIFE and factor this into all projections of power below. If loss to follow-up accrues at 7%/yr or 9%/yr rather than 8%/yr, the power for detecting the specified effect sizes would be increased or decreased by only 1%.

Inference: Our projections are based on a log-rank test with **2-sided significance level 0.05**. We have not adjusted this for interim analyses; however, the approach we recommend (Chapter 9) will not alter the final critical value materially.

Calculations: We have used SAS Proc Power, to allow incorporation of the increase in event rates during follow-up years 2-3.7 into our power calculations. In using this procedure, we assumed a uniform recruitment rate over 21 months. We have used the simulation option of NQuery Advisor to allow for calculation of power assuming recruitment of 50% of participants in the final 9 months of the 21 month recruitment period.

Results: Table 3.3.1. contains the power to detect relative effect sizes ranging from 20% to 25% for our total sample size of **1,600**, assuming uniform recruitment over 21 months. Using NQuery Advisor, and assuming the non-uniform recruitment schedule previously mentioned, we find that the power figures reported in Table 3.3.1 remain the same or decrease by less than 1% for any assumed effect size.

Table 3.3.1 Power for Relative Effect Sizes

**20%**	**21%**	**22%**	**23%**	**24%**	**25%**
76%	80%	84%	87%	90%	93%

Because of ethical concerns related to withholding the possibility of randomization to participants far along in the screening process that have been found to be eligible after the targeted sample size has been reached, LIFE will allow recruitment for up to a total of 1680 participants. This will allow each site to recruit an additional 10 participants that are in the screening "pipeline" at the time that the targeted 200 per site is reached. This recruitment would result in the power estimates in Table A should the full 1680 participants be recruited.

Table A. Power for Relative Effect Sizes (N=1680)

**20%**	**21%**	**22%**	**23%**	**24%**	**25%**
78%	82%	86%	89%	92%	94%

The **secondary aims** of the LIFE Study are to assess the relative effect of the intervention on the following **secondary outcomes**:

- Cognitive function;
- Serious fall injuries;
- Persistent mobility disability and the combined outcome of major mobility disability or death;
- Proportion of 400 m walk failures over time;
- Disability in activities of daily living; and
- Cost-effectiveness

We have also examined the power available for secondary outcomes.

The focus for assessing the relative effect of the PA intervention on cognition is based on two secondary cognitive outcomes: the Digit Symbol Substitution Test (DSST) and the Hopkins Verbal Learning Test (HVLT). Each will be tested at the 2-sided alpha=0.05 level. LIFE-P[88] results, and data from other cognitive trials we have conducted (e.g. WHISCA,[98] CoSTAR, GEMS[99]), indicate that longitudinal correlations of these measures are likely to range from r=0.50 to r=0.70 over 4 yrs. In GEMS, [99] at 4 yrs 92% of all expected face-to-face cognitive assessments (3MSE, CDR and ADAS-COG) were completed in a population of adults age $\geq$75 years (mean 79 years). In LIFE-P, retention was very strong at 12 months. Meta-analyses report that measures of executive function on average are relatively improved by 0.3 standard deviations (SD) by aerobic exercise interventions.[100-102] Since most trials contributing to these meta-analyses were conducted in more healthy cohorts and are of shorter duration than LIFE, we anticipate that the effect size in LIFE will be less: we target an average effect size of half of this (e.g. 0.15 SD). We project from LIFE-P that this translates to average effects of 1.8 (DSST; and 0.8 (HVLT) units.[103] Data

from the Women's Health Initiative Memory Study (WHIMS)[104] indicate that annual assessments are not optimum in many cognitive trials, thus we will collect data only at baseline and year 2 (and assume an 8%/yr accumulating lost follow-up). Standard power calculations (i.e. a covariate-adjusted z-test with Bonferroni critical value of 1.96) provide the powers listed in Table 3.3.2 associated with drop-in/drop-out rates of 0%, 10% and 20% for both arms and exam times (i.e., reducing the difference between arms by these amounts).

**Table 3.3.2**	**Projected Power: N=1,600 Enrolled**		
	**Cross-Over Rates (applied equally to both arms and exam times)**		
**Longitudinal Correlation**	0%	10%	20%
0.50	89%	82%	72%
0.60	93%	87%	79%
0.70	97%	94%	87%

Other Secondary Outcomes: We have also projected the power to detect differences in other secondary outcomes. We adopt 2-sided Type 1 error of 0.05 for these outcomes. For **serious fall injuries**, during LIFE-P, we encountered 7 adjudicated serious falls among the N=211 SA participants and 3 serious falls among the 213 PA participants (p=0.22). The cumulative hazards (based on Kaplan-Meier plots) of falls in the SA participants were 0.032 (12 months) and 0.060 (18 months). If we conservatively project forward a 3%/yr incidence rate of new injurious falls in the SA participants and use methods similar to those described above for the primary outcome, we project we will have 80% and 90% power to detect relative hazard ratios of **1.84** and **2.05**, respectively. We expect power for **disability in ADLs** to be similar to what we project from the FAST Disability Score: 80% (90%) power to detect reductions in the rates of adverse changes of **67%** (**78%**).[8] **Persistent mobility disability** is operationally defined as failure to complete a 400 m walk at two successive 6 month exams. We have estimated and projected forward transition probabilities for persistent disability.[105] A sample size of **n=1,600** participants is expected to allow the detection of effect sizes of **85%** and **98%** with 80% and 90% power. We expect the power for **combined major mobility disorder or death** to be similar to that for our primary outcome.

Additional **tertiary** aims of the LIFE trial are to explore the effects of the intervention on Mild Cognitive Impairment / Dementia (MCI/D).

**Table 3.3.3 Group**	**Effect Size**	**2%/yr SA Incidence Rate**		**2.5%/yr SA Incidence Rate**		**3%/yr SA Incidence Rate**	
		**Cumulative 2-yr New Cases**	**Power**	**Cumulative 2-yr New Cases**	**Power**	**Cumulative 2-yr New Cases**	**Power**
**SA**	**---**	27		34		40	
**PA**	**40%**	16	33%	20	43%	24	48%
	**50%**	13	55%	17	62%	20	70%
	**60%**	10	75%	13	84%	16	88%

We expect the **prevalence** of MCI/D in the LIFE cohort at baseline to be much less than in the community due to a "healthy volunteer" effect and the trial's eligibility criteria. For example, WHIMS found that only 11/7482 (0.15%) enrollees from among Women's Health Initiative trial participants met study criteria for MCI/D.[98] The rate will likely be higher among LIFE participants, who are selected to be more physically compromised than WHIMS participants, but is difficult to predict. We adopt a baseline prevalence of 10% in our calculations of power. We apply the 8%/yr **lost to follow-up rate** adopted for other LIFE outcomes. This translates to data collection rates of 84.6% at 2 years. The **incidence** of MCI/D in RCTs and cohort studies ranges from 1-4%/yr.[98, 104, 106-110] The LIFE cohort will be selected to be at increased risk for mobility disability, and is thus also expected to be at increased risk for cognitive decline.[14] We use a rate of 2.5%/yr in our sample size

projections, but also examine the power for rates of 2.0%/yr and 3.0%/yr. We will explore **effect sizes** of 40-60%. Factoring in the baseline prevalence and follow-up rates we obtain the results contained in Table 3.3.3 LIFE is projected to provide **70%** statistical power to detect an effect of 50% if SA incidence is 3%/yr.

Power for combined cardiovascular events is presented in Table 3.3.4. We have assumed the same 8%/year loss to follow-up assumed by the LIFE main trial. We expect that the annual event rate is approximately 4%/year but have also calculated power for 2% and 6%/year. Using a time-to-event analysis, we will have 51% power for a 30% effect (HR of 0.7) using a two-sided test at the 5% level. Power ranges between 29 and 99% in these scenarios, depending on the background event rate and the effect size. Power for hospitalized pulmonary disorders is presented in Table 3.3.5. Calculations were similar as for CVD event power. Because of the lower expected event rates (even with larger assumed effect sizes), the power is considerably lower. Power for ventilatory capacity is presented in Table 3.3.6. For simplicity and to be conservative, we have assumed an analysis of covariance model with adjustment for baseline but have not accounted for multiple follow-up measurements. A mixed model will ultimately be used to account for this correlation. Assumed standard deviations were 0.57 for FEV1 and 0.66 for FVC.[111] With effect sizes of 0.05 units, there is modest power, but effect sizes of 0.1 there is substantial power. Power for sleep wake disturbances are presented in Table 3.3.7. As above, we assume 8%/year loss to follow-up. With a 5%/year event rate and a 30% effect (hazard ratio of 0.7), we would have 59% power to detect an effect using a two-sided test at the 5% level.

Table 3.3.4. CVD Event Power

Effect Size	2%/yr SA Incidence Rate	4%/yr SA Incidence Rate	6%/yr SA Incidence Rate
**30%**	29%	51%	67%
**40%**	49%	78%	91%
**50%**	70%	94%	99%

Table 3.3.5. Hospitalized Pulm Event Power

Effect Size	0.1%/yr SA Incidence Rate	0.5%/yr SA Incidence Rate	1%/yr SA Incidence Rate
**40%**	7%	16%	28%
**50%**	9%	24%	42%
**60%**	11%	34%	59%

Table 3.3.6 Ventilatory Capacity Power

Corre-lation	Effect Size	FEV1	FVC
**0.3**	**0.05**	45%	36%
	**0.1**	96%	89%
**0.5**	**0.05**	53%	42%
	**0.1**	98%	94%
**0.7**	**0.05**	69%	56%
	**0.1**	>99%	99%

Table 3.3.7. Sleep-wake disturbance Power

Effect Size	3%/yr SA Incidence Rate	5%/yr SA Incidence Rate	7%/yr SA Incidence Rate
**20%**	20%	30%	39%
**30%**	40%	59%	73%
**40%**	66%	86%	95%

4. Study Population

The eligibility criteria for the LIFE Study identify participants who are not currently disabled but have moderate to high risk for occurrence of mobility disability and for whom the intervention is safe.

The Coordinating Center monitors the distribution of the recruited cohort with respect to age, gender, ethnicity, score on the Short Physical Performance Battery (SPPB) and other factors expected to influence the incidence rate of the trial's primary outcome. Based on this monitoring activity, targeted recruitment strategies are developed to ensure that the study cohort is racially and ethnically diverse and has a range of age and physical performance adequate to evaluate the results of this study.

4.1. Eligibility Criteria

4.1.1. Targeting Populations at High Risk of Disability

Targeting the non-disabled but high-risk segment of the older population for a physical activity program aimed at reducing disability has many advantages. These persons are in the middle of the functional spectrum and are neither so disabled that a physical activity program may not offer help nor so highly functional that their already very low risk of becoming disabled would not be appreciably affected by the intervention. They may be at a transitional stage in the pathway to disability, so that a well-focused intervention could be extremely effective in pulling them back from the brink of disability onset and lead to additional years of disability-free life.

Most of the older population is non-disabled and an important goal in this segment of the population is to prevent or postpone the onset of disability. However, there is a great deal of heterogeneity in the non-disabled portion of the older population and any strategy to prevent disability should take the very broad range of health status into account. Some older non-disabled persons are already very active and vigorous while others are sedentary and may actually have impairments and functional limitations that indicate an elevated risk of disability. The eligibility criteria in this study are aimed at identifying persons who are sedentary, have functional limitations, as assessed by a battery of physical performance tests, but who have not yet developed disability, as documented by their ability to walk 400 meters without sitting or the help of another person. Targeting this subset of the population makes it possible to recruit a non-disabled but at risk population for a clinical trial of disability prevention. In addition, evaluating the role of physical activity in preventing the onset of mild cognitive impairment and Alzheimer's disease is an important secondary aim of the LIFE Study so its cohort will also be selected to be free of significant cognitive impairment. This is a large segment of the older population in which successful prevention of disability onset, in this case through a physical activity program, would have a major public health impact.

4.1.2. Establishing Eligibility

Eligibility is established in a multi-step screening process. The first step is a telephone screen to assess specific inclusion and exclusion criteria. This is followed by an interviewer assessment, including the administration of the SPPB, the 400 meter walk test, the Modified Mini-Mental State (3MSE) exam and an interview. Finally, the potential participant receives an examination by the study physician, physician assistant or nurse practitioner, who determines if conditions are present that meet exclusion criteria. Eligibility criteria are as follows:

Gender Men and women are eligible. The LIFE Study endeavors to recruit men and women in rough proportion to their representation in the catchment area population.

Age Individuals aged 70-<90 years old are eligible. This age group is selected because it is at high risk of major mobility disability,[90] and it may have a sufficiently long life expectancy[1] to participate in a full-scale RCT, which would have a duration of 3 to 4 years.

Ethnicity All ethnic groups are eligible for the study. The LIFE Study goal is for a study cohort that is at least 22.5% from minority populations (primarily African Americans and Hispanic Americans).

Residency Participants must be planning to reside in the area for at least 2 years.

Functional Status Summary score <10 on the Short Physical Performance Battery (SPPB).[90] Ability to complete the 400 m walk test within 15 minutes without sitting or the help of another person, or the use of a walker. The LIFE Study goal is a target of 45% of randomized participants to have a score of < 8.

Cognitive functioning Persons are eligible if they do not report a diagnosis of dementia or score lower than our education-based cut-points on the Modified Mini-Mental State Exam (3MSE). Persons with 9 or more years of education who score <80 (<76 if African American) and those with less than 9 years of education who score <76 (<70 if African American or Spanish speaking) are excluded. [205-207] Persons excluded because of low 3MSE scores will be advised by the research staff to take the results to his/her PCP for additional review since our testing is for research purposes only.

Physical activity and exercise Sedentary lifestyle is operationally defined as spending less than 20 minutes per week in the past month getting regular physical activity. Physical activity includes activities like: brisk walking, jogging, weight lifting, cycling, aerobics, and dancing. In addition, reports less than 125 min/week of moderate physical activity based on the modified 18-item CHAMPS questionnaire.

Chronic disease status The LIFE Study recruits individuals both with and without chronic diseases, except for specific conditions described in the exclusion section that may be life-shortening or prevent the participation in a physical activity intervention.

Willingness to participate Participants must be willing to give informed consent, be willing to be randomized to either Physical Activity or the Successful Aging Program intervention, and to follow the protocol for the group to which they have been assigned.

4.2. Exclusion Criteria

Individuals are excluded from participation in the study for any of the following reasons: 1) the potential participant may have difficulty adhering to either intervention, 2) participation may be unsafe, 3) the participant has serious health conditions that would interfere with the intervention goals, and/or 4) the participant is already physically active to a degree that the adoption of an activity program would be of little additional benefit.

In many cases, participants may have conditions that would preclude participation in the study that could resolve. Therefore, we also define a set of temporary exclusions. Participants with such exclusions may be re-contacted later during the recruitment period for further evaluation.

4.2.1. Exclusion Criteria for Factors that May Limit Adherence to Interventions or Affect Conduct of the Trial

- Unable or unwilling to give informed consent or accept randomization in either study group
- Current diagnosis of schizophrenia, other psychotic disorders, or bipolar disorder
- Current consumption of more than 14 alcoholic drinks per week
- Plans to relocate to out of the study area within the next 2 years or plans to be out of the study area for more than 6 consecutive weeks in the next year
- Self-reported inability to walk across a small room
- The use of a walker to complete the 400 m walk and/or unable to complete the 400 m walk without sitting down or the help of another person
- Another member of the household is a participant in the Life Study
- Residence too far from the intervention site
- Residence in a nursing home
- Difficulty in communication with study personnel due to speech or hearing problems
- 3MSE score below the cutoff for education:

**Education**	African American	English Speaking Non- African American	Spanish Speaking
9+ years	76	80	80
<9 years	70	76	70

- Participation in LIFE-Pilot study
- Other medical, psychiatric, or behavioral factors that in the judgment of the Principal Investigator may interfere with study participation or the ability to follow the intervention protocol

4.2.2. Exclusion Criteria for Underlying Diseases Likely to Limit Lifespan and/or Affect the Safety of the Interventions

- Severe arthritis (either osteoarthritis or rheumatoid arthritis)
- Cancer requiring treatment in the past three years, except for non-melanoma skin cancers or cancers that have clearly been cured or in the opinion of the investigator carry an excellent prognosis (e.g., Stage 1 cervical cancer)
- Lung disease requiring either regular use of corticosteroid pills or injections or the use of supplemental oxygen
- Development of chest pain or severe shortness of breath on a 400 m self-paced walk test
- Cardiovascular disease (including NYHA Class III or IV congestive heart failure, clinically significant aortic stenosis, history or cardiac arrest, use of a cardiac defibrillator or uncontrolled angina)
- Parkinson’s disease or other serious neurological disorder
- Renal disease requiring dialysis
- Other illness of such severity that life expectancy is considered to be less than 12 months
- Conditions not specifically mentioned above may serve as criteria for exclusion at the discretion of the clinical site

4.2.3. Temporary Exclusion Criteria

- Uncontrolled hypertension (systolic blood pressure > 200 mmHg and/or diastolic blood pressure > 110 mmHg).
- Uncontrolled diabetes with recent weight loss, diabetic coma or frequent insulin reactions.
- Stroke, hip fracture, hip or knee replacement, or spinal surgery in the past 6 months.
- Serious conduction disorder (e.g., 3rd degree heart block), uncontrolled arrhythmia, or new Q waves or ST-segment depressions (>3 mm) on ECG.
- Myocardial infarction, major heart surgery (i.e., valve replacement or bypass surgery), stroke, deep vein thrombosis or pulmonary embolus in the past 6 months.
- Undergoing physical therapy or cardiopulmonary rehabilitation
- Currently enrolled in another randomized trial involving lifestyle or pharmaceutical interventions

5. Recruitment and Retention

5.1. Recruitment

The recruitment goal of the study is to enroll 1,600 participants, approximately 200 at each of the 8 clinical sites. Participants are recruited over a 21 month period with a goal of overall minority participation of at least 22.5%. It is a recruitment goal of the Life Study that at least 45% of participants have a baseline SPPB (Short Physical Performance Battery) score of 7 or below. In order that barriers to their recruitment are minimized, this recruitment goal will not apply to ethnic minorities. All recruitment related activities are overseen by the Recruitment, Adherence and Retention Committee. The Committee coordinates press and media and assists the sites in the preparation of recruitment materials. Each clinical site develops a site-specific recruitment plan, in order to accommodate the variability across centers in catchment area characteristics, media market outlets, and access to older participants. Recruitment strategies include the use of newspaper, radio and television advertisements, direct mail, and presentations at health fairs, senior centers, medical clinics, and churches. Participants in previous studies may also be approached and ineligible participants are asked about friends who might be eligible. All recruitment materials are reviewed by the appropriate field center IRB before being used.

5.1.1. Screening Process

The purpose of the staged screening process is to identify and verify eligible participants over a series of contacts. Interested participants are first screened by phone. The phone interview is designed to exclude individuals who are clearly ineligible or unlikely to benefit from participation in the study. At the first screening clinic visit, medical and functional exclusions are assessed, including those based on lower extremity physical function. At the second clinic visit, further medical information is collected and a final eligibility determination is made prior to randomization. The exclusion criteria likely to have the largest impact on eligibility are having a score on the short physical performance battery (SPPB) of 10 or above and reporting $\geq$125 min of moderate level exercise in the CHAMPS-18 questionnaire. To save clinic time and expense, the SPPB and the CHAMPS-18 questionnaire can be administered off-site or in a modular form during the screening process.

5.2. Retention and Drop-out Recovery

5.2.1. Identifying Secondary/Proxy Contacts

Although not a criterion for enrollment in the trial, LIFE attempts to identify a proxy respondent for all participants. A proxy respondent and two additional contact persons are identified and may be contacted to provide supplemental information on the participant.

5.2.2. Retention Promotion Efforts

During screening, participants are informed about which clinically relevant test results they are to receive and when these tests are performed during the course of the study. Such test results are provided to the participant at the end of study visits.

Before enrollment, preventive measures are taken to minimize participant non-compliance related to data collection. Because the study requires a dedicated commitment to examination schedules, only those subjects who fully understand these commitments and appear likely to follow the study protocol are enrolled. The judgment of Field Center staff is essential in determining overall eligibility with respect to adherence.

Providing clear, easy-to-follow, written instructions about when to return for follow-up visits is important. Reviewing these instructions with the participant periodically during follow-up is a priority, especially if demonstrated compliance problems exist. Involving the subject's spouse or other family members in these reviews can be useful. Attempts are made to maintain continuity of follow-up care, so that, whenever possible, the same staff

member sees the subject throughout the study. Every attempt is made to make all clinic visits pleasant. Minimizing waiting time and providing parking spaces, free transportation for the clinic assessment visits, and comfortable waiting room facilities makes the visits more pleasant, thereby enhancing participant retention in follow-up appointments.

During the follow-up phase, participants attend clinic visits every six months. If they are unable to come to the clinic, home or institutional visits are scheduled. Telephone or proxy interviews are scheduled if in-person visits cannot be completed. Attendance at scheduled visits is documented by completion of the Follow-up Visit Checklist and Missed Visit forms. **Field Centers are advised to keep detailed records of rescheduled and broken appointments for each participant. Participant retention is monitored, and efforts are made to identify those individuals who need support and encouragement.** Records of participants consenting to only a portion of the follow-up procedures, i.e., partial compliance, are also maintained. Summary reports of such difficulties help to identify problems. Critical review of such problems may offer potential solutions.

5.2.3. Drop-out Recovery Efforts

The following procedures are implemented (as appropriate in each Field Center) to carefully document and monitor missed clinic or home visits:

- Preparing for the next visit at the end of each current visit by making the appointment and giving instructions for the next visit.
- Sending out pre-visit reminders (e.g., postcards and phone calls).
- Establishing a mechanism to chart and monitor local clinic attendance, so that clinic staff would be immediately alerted to a missed visit.
- Immediately contacting participants (usually by telephone) when they miss a visit.
- Planning clinic action to rectify the problem within the scope of clinic services.
- Rescheduling the visit within the same window, if possible. Examinations that fall outside of the target window remain important and are used in all analyses. These examinations are assigned to whichever target visit would be the closest in time. If it becomes clear that a visit corresponding to a particular set of forms (e.g., a 6-month visit) is not completed, a Missed Visit form is filled out.

Some randomized participants may not actively participate in the study, perhaps by not adhering to the intervention and/or not attending the clinic. This may be due to a number of reasons, such as family objections to participation, or participant decision. Regardless of the reason(s), these participants are followed until the end of the study, and clinic staff attempts to make contact every six months after the baseline assessment. These contacts are intended to remind the participant that they are welcome to fully rejoin the study at any time. Considerable effort is expended to collect main outcome data at appropriate times.

The following guidelines promote adherence to the protocol, in terms of intervention adherence and clinic attendance. The availability of local clinic resources determines which techniques are used.

- **Participant-staff relationship**. A key element contributing to participants' continued commitment to the trial involves fostering positive, respectful relationships between study subjects and individual members of the staff.
- **Continuity of care**. In general, participants' appointments should be scheduled so that they can be seen by the same clinic staff members during each visit.
- **Clinic environment**. The clinic environment which is warm and pleasant, and

oriented to the comfort of the participant.

- **Participant-staff communications**. Good and consistent communication is essential. Instructions are clear and interactions are friendly and individualized. The participant is reminded of the benefits of study participation. Written reminders about clinic appointments further enhance communication efforts. Unmasked clinic staff meet regularly with intervention staff to reinforce the importance of consistency of communications across intervention groups.
- **Convenience and accessibility**. An easily accessible clinic location, availability of transportation, and convenient clinic hours all serve to facilitate study adherence. Field Centers make study visits as easy as possible for participants, a factor critical to the success of the study. All sites take steps to ensure that clinic attendance is not compromised by a lack of transportation, unsuitable hours of clinic operation, or any similar circumstance. If necessary, participants are reimbursed for or are provided transportation to the clinic assessment visits.
- **Time in clinic**. Total clinic visit time is kept to a minimum, consistent with maintaining quality. If waiting is necessary, the situation is explained to the participant and, if possible, an offer is made for the participant to see another staff member, or to reschedule the visit. On the other hand, participants are not rushed or made to feel unwelcome. Clinic staff is trained to take time to visit with participants.
- **Appointment reminders**. Appointment reminders are used to prompt participants to come for clinic visits. These written reminders are mailed to participants so that they receive them one to two weeks before their scheduled visit date.

5.3. Monitoring Recruitment and Retention

The Recruitment, Adherence and Retention Committee routinely monitors screening and recruitment yields, and compares them to preset gender, ethnic minority and SPPB score benchmarks for each site. If these benchmarks are not attained, the main reasons for exclusion of subjects are analyzed and the recruitment strategies are modified accordingly. The Recruitment, Adherence and Retention Committee may also recommend changes in the protocol, if needed. Reports on recruitment are generated and are reviewed by the Steering Committee, the Data and Safety Monitoring Board, and the NIA Project Office.

5.3.1. Retention and Efforts to Maintain Contact with Inactive Participants

Retention is promoted by:

1. Examining and attempting to remove barriers (e.g., by addressing parking and other transportation issues, adjusting clinic hours);
2. Incorporating a variety of methods to promote contact with all participants and provide social support for all participants, including those in the Successful Aging attention-control arm;
3. Providing all staff and investigators who have contact with LIFE participants with training and regular re-training in motivational methods; and
4. Ensuring that participants' concerns are identified and addressed before they express a desire to reduce their involvement in the study.

Efforts to Maintain Contact with Inactive Participants

LIFE has the goal of maintaining some form of contact (e.g., phone, e-mail) with participants who are unable to continue full engagement in the study and to foster some form of continued contact (e.g., even an agreement to allow future contact) with participants who are inactive in the study. The greatest importance is given to attending semi-annual assessment visits; even participants who are unwilling to continue attending intervention sessions are strongly encouraged to attend the assessment visits.

5.3.2. Monitoring and Quality Control of Recruitment and Retention

The DMAQC center collects data to monitor recruitment and retention activities, the number of potential participants contacting each site, how potential participants indicate that they heard about the study, the yield at the various screening steps, and follow-up rates. Regular web-based reports are available to field centers and the LIFE Recruitment Adherence and Retention Committee. Members of this committee maintain regular phone contact with clinic staff to:

1. Review recruitment goals and yields for all centers participating on each call,
2. Review the recruitment plan and progress in achieving the objectives outlined in the plan,
3. Share successful and unsuccessful recruitment methods, and
4. Review retention.

If centers encounter difficulties in recruitment, the Recruitment Adherence and Retention Committee (or a subgroup it designates) provides a graduated set of assistance responses that are based on the degree of recruitment shortfall. If retention becomes a problem for a clinic, a graded response of assistance that is based on clinic-specific retention issues is provided.

6. Measures and Procedures
6.1. Informed Consent
Informed consent must be obtained before participants are screened. Verbal consent is acquired prior to the administration of the telephone screen. Clinics are allowed to elect, as their IRB requires either a single consent procedure to cover consent for participation in the entire study, or a staged consent procedure in which they are asked to provide initial consent to participate in the screening followed by, for those who qualify, later consent to participate in the remainder of the study.

6.2. Measures
6.2.1. 400 Meter Walk Test
The primary outcome for the LIFE trial is time to the onset of major mobility disability. The objective outcome of major mobility disability is defined as the inability to complete a 400 m walk within 15 minutes without sitting, using a walker, or the help of another person. Major mobility disability is assessed every six months by staff who are blinded to the intervention.

6.2.2. Short Physical Performance Battery (SPPB)
The SPPB, originally developed for the Established Populations for the Epidemiologic Study of the Elderly (EPESE) is a brief performance battery based on timed short distance walk, repeated chair stands and balance test (as described by Guralnik et al.)[90, 112-116] The battery is administered by trained examiners. The measurement goal for this battery is to assess lower extremity functional limitations, which indicate functional abilities and are a strong measure of risk for future disability. The test takes about 10-15 minutes to administer and can be done in the home or the clinic setting. The battery has an excellent safety record. It has been administered to over 20,000 persons in various studies and no serious injuries are known to have occurred. The components of the battery are as follows:

Walking speed. Walking speed is assessed by asking the participants to walk at their usual pace over a 4 m course. Participants are instructed to stand with both feet touching the starting line and to start walking after a specific verbal command. Participants are allowed to use walking aids (cane, walker, or other walking aid) if necessary, but not the assistance of another person. At the screening visit, those participants who must use a walker are excluded. Timing begins when the foot starts to move across the starting line and the time in seconds needed to complete the entire distance is recorded. The faster of two walks is used to compute walking speed.

Chair stands. The repeated chair stands test is performed using a straight-backed chair, which is placed with its back against a wall. Participants are first asked to stand once from a sitting position without using their arms. If they are able to perform the task, they are then asked to stand up and sit down five times, as quickly as possible. The time to complete the task is recorded.

Standing balance. For the test of standing balance, participants are asked to maintain balance in three positions, characterized by a progressive narrowing of the base support:, with feet together (side by side position), the heel of one foot beside the big toe of the other foot (semi tandem position), and the heel of one foot in front of and touching the toes of the other foot (tandem position). For each of the three positions, participants are timed to a maximum of 10 seconds. Scores are summed for the measure of balance for a range of 0 to 30 seconds.

Summary performance score. Each of the three performance measures is assigned a score ranging from 0 to 4, with 4 indicating the highest level of performance and 0 the inability to complete the test. For the test of balance, participants are assigned a

score of 1 if they can hold a side-by-side standing position for 10 seconds but are unable to hold a semi-tandem position for 10 seconds; a score of 2 is assigned if they can hold a semi-tandem position for 10 seconds, but are unable to hold a full-tandem position for 3 seconds; a score of 3 is assigned if they can stand in a full-tandem position for 3 seconds but less than 10 seconds; a score of 4 is assigned if they can stand in a full-tandem position for 10 seconds.

Four categories are computed for walking speed and chair stands, according to cut points that are based on quartiles of the time to perform each task assessed in the EPESE.[90] The time of the faster of two walks is scored as follows: > 8.7 sec = 1; 6.21 to 8.70 sec = 2; 4.82 to 6.20 sec = 3; < 4.82 sec = 4; a score of 0 is assigned to participants unable to perform the test. The time required to perform five chair stands is scored as follows: ≥ 16.70 sec = 1; 13.70 to 16.69 m/sec = 2; 11.20 to 13.69 m/sec = 3; ≤ 11.19 = 4. A score of 0 is assigned to participants unable to perform the task. A summary score ranging from 0 (worst performers) to 12 (best performers) is calculated by adding walking speed, chair stands and balance scores. This scale has proven reliable[117] and valid for predicting institutionalization, hospital admission, mortality and disability,[114] and it is used for participant screening and as a tertiary outcome**.**[90, 115, 116, 118] It will be administered at the first screening visit, and at the 6, 12, 24, 36 month, close-out and PIV visits. The SPPB will also be completed at any visit at which the 400 m walk test is not attempted.

6.2.3. The Mobility Assessment Tool (short form): MAT-sf.

The MAT-sf is a 10-item computer based assessment of mobility using animated video clips. The 10 items in the MAT-sf cover a broad range of functioning. The items include walking on level ground, a slow jog, walking outdoors on uneven terrain, walking up a ramp with and without using a handrail, stepping over hurdles, ascending and descending stairs with and without the use of a handrail, and climbing stairs while carrying bags. The items were selected based on individual response and information curves derived from Item Response Theory. Each item is accompanied by an animated video clip together with the responses for that question (number of minutes, number of times, yes/no). The test can be done on any laptop and scores are saved to an exportable file. The time required to do the test with instructions from the examiner is <5 min. Scores for the MAT-sf have been standardized with a mean of 50 and a standard deviation of 10. A psychometric paper describing the reliability and validity of the measure is currently under review (Rejeski et al., 2009, under review). The MAT-sf is administered at baseline, 18-month and 30-month visits.

6.2.4. Hand Grip Strength

Hand grip strength is a commonly used measure of upper body skeletal muscle function and has been widely used as a general indicator of frailty with predictive validity for both mortality and functional limitation.[119, 120] Grip strength is measured in the dominant hand using a hydraulic grip strength dynamometer at baseline, the 12 month, and the PIV visits. If the participant reports current flare-up of pain in the dominant wrist or hand, or has undergone fusion, arthroplasty, tendon repair, synovectomy, or other related surgery of the dominant hand or wrist in the past 3 months, the other hand should be tested. Other than possible temporary discomfort during the test itself, there are no known risks for the participant.

6.2.5. Self-Reported Physical Function/Disability and Physical Activity – Accelerometry

Self-Reported Physical Function/Disability is assessed with a modified version of

disability instrument that was used in LIFE-P, now called the Pepper Assessment Tool for Disability (PAT-D).[121-123] Based on factor analysis, 4 items were omitted from the original instrument (doing errands, preparing meals, feeding, and raising arms above head), leaving 19 items covering 3 domains: (1) basic ADLs (moving in and out of a chair, moving in and out of a bed, gripping with hands, using toilet, dressing, getting in and out of a car, and bathing); (2) mobility (walking several blocks, lifting heavy objects, walking 1 block, lifting/carrying 10 lbs, climbing several flights of stairs, and climbing 1 flight of stairs); and (3) instrumental ADLs (light housework, participating in community activities, managing money, visiting with relatives or friends, using the telephone, and taking care of a family member).

For each item, respondents answer whether they experience 1) no difficulty, 2) a little difficulty, 3) some difficulty, 4) a lot of difficulty, 5) unable to do or, 6) did not do for other reasons. Answers are averaged across the items, in order to better assess the overall perceived disability burden by a person.

As in LIFE-P, we will add two items to the disability instrument, giving a total of 21 items. These are “walking across a small room” and "walking a quarter of a mile.” These two items have been used previously as the single outcome of interest for studies on mobility disability.[55] In addition, for the basic ADLs (including walk across a room, but not gripping with hands) we plan to ask whether the participant receives help from another person to complete the task. This will allow us to calculate a Katz ADL score. The disability questionnaire will be administered at the first screening visit, and at all subsequent visits. An abbreviated Disability Questionnaire will be administered at the 18, 30, and 42 month visits.

A proxy ADL questionnaire is administered when a participant is not available to complete a follow-up assessment or is deemed to be cognitively impaired based on the Six-item screener. The assistive device questionnaire inquiring about the use of a walker or cane is administered for all visits at which the 400 m walk test is not attempted.

Self reported physical activity is monitored by means of the Community Healthy Activities Model Program for Seniors (CHAMPS) Activities Questionnaire a validated questionnaire that takes about 15 min to complete. [124 125] The questionnaire assesses the weekly frequency and duration of various physical activities typically undertaken by older adults. This instrument is administered at the first screening visit, and at the 6-, 12-, 24-, 36-month, close-out, and PIV assessment visits. In addition, for a subset of activities, the specific amount of time will be ascertained to supplement the categorical response. A slightly modified version that includes 18 items will be used (CHAMPS-18).

Accelerometry

The ActiGraph GT3X (ActiGraph™ LLC, Pensacola, FL) is used to assess the relative impact of interventions on physical activity over time. The ActiGraph is a small (3.8 cm x 3.7 cm x 1.8 cm, 27g) triaxial accelerometer that is designed to detect accelerations in three axes. Output from the ActiGraph is in the form of step counts, body positions (standing and sitting/lying) and activity counts for a specific time period (i.e., epoch). Activity count cut-points (e.g., $counts \cdot min^{-1}$) can be identified to determine the amount of time a participant spends in sedentary, light, moderate, hard, or vigorous activity. Accelerometry will be measured for 7 consecutive days at baseline, 6, 12, 24-month, and the PIV visits, in all participants randomized at each site.

Determination of activity count cut-points. Individualized activity count cut-points for each participant will be determined during the 400-m walk. The average $count \cdot min^{-1}$ generated by a participant during the 400-m walk will serve as his/her individualized activity cut-point and can be used to evaluate the 7-day accelerometry data. The ActiGraph will be initialized prior to the participant’s assessment visit and

programmed to record data in one-second epochs.

The monitor is then attached to the participant's waistline using a belt and placed along the right mid-axillary line. Staff will record the start time and end time for the walk from the computer laptop used to initialize the monitor. The beginning and end of the 400-m walk will be identified on the ActiGraph through visual inspection and the time stamp corresponding to the time when the 400-m walk occurred. As a result, the activity counts associated with the 400-m walk can be easily identified during subsequent analyses.

7-day accelerometry. At the conclusion of the walk, participants will keep the monitor on and wear it for the 7 days immediately following their clinic visit. During the 7-day monitoring period, participants will be asked to put the monitor on each morning (after dressing) and remove the monitor just prior to going to bed at night. The monitor will also be removed for bathing, showering, or any other activity that might result in exposure to water. Participants will be instructed to contact research staff by telephone with any questions about monitor use. Following the 7th complete day of activity monitoring, participants will return their activity monitor to research staff via mail.

Accelerometry data from the 400-m walk and 7-day assessment will be downloaded from the monitor and securely transmitted to DMAQC for data cleaning and analysis.

6.2.6. Process Measures

A brief battery of tests is employed to evaluate psychological processes that are theoretically linked to adherence and success with the interventions. These include items related to performance efficacy, barriers efficacy, satisfaction with function and motivation for physical competence. This brief test battery is collected via self-administration on all participants at baseline, 12 months and 30 months. At baseline, a survey of each participant's local environment will also be obtained to examine the role of physical environmental barriers on physical activity participation and adherence.

6.2.7. Vital Signs

Prior to randomization and the 6, 18, 30 months, and PIV visits, data are collected on sitting blood pressure, heart rate, and weight. Body height is measured once prior to randomization. Waist circumference is measured at baseline and at the 24 months visit. The blood pressure assessments will allow the determination of the incidence of hypertension and serve as basis for a temporary exclusion. The other measures are collected primarily for descriptive purposes.

6.2.8. Medication Inventory

Many older adults use both prescription and non-prescription pharmaceutical products. The use of these products is of interest for several reasons. Their use is an important indicator of overall health, and the nature of the drugs taken is a strong indicator of clinically manifest disease. The response to the intervention may be enhanced or diminished by some drugs. Finally, individuals who use nutritional supplements, herbs or other complementary products may have a stronger sense of health self-efficacy, and thus the use of these products could be related to study adherence. All participants are asked to bring all prescription and non-prescription medications taken in the past two weeks to their first pre-randomization screening visit and subsequent annual follow-up visit. Medications include: pills, tables, drops, salves, injections, creams/ointments, inhalers, suppositories and dermal patches. Non-prescription medications include: vitamins, aspirin, laxatives, dietary supplements, and herbal preparations. The name, strength and formulation of each product are transcribed. These medications are coded according to

formulation for use in subsequent data analyses. This method of drug assessment has been shown to be valid in older adults.[126] The medication inventory is administered at baseline and at the 12 month clinic visit.

6.2.9. ECG

A twelve lead ECG is performed at the initial visit for safety purposes. The ECG is reviewed by the study physician at each field center to assess potential exclusion criteria. In addition, to assess silent myocardial infarction, ECG will be performed at 18 months, and at closeout only if the measures were missed at 18-month follow-up visit or if the 18-month follow-up visit occurred more than one year prior to the closeout visit.

6.2.10. Social, Economic and Health Related Questions

For descriptive purposes, the following participant characteristics are collected: age, gender, race, living situation, household composition, marital status, educational level, smoking status, alcohol consumed, employment status, occupation, volunteer work, income level and chronic conditions.

6.2.11. Cognition

Cognitive function is assessed at baseline (both computer battery and non-computer based battery) and at 18 (computer battery only), 24-months (non-computer based battery only), and at the PIV (non-computer based battery only) visits in all participants. Participants who are unable to take the battery components at 18 or 24 months will be allowed to take them at the 30 month visit. The LIFE Study cognition assessment battery measures were selected because they have been used often in research examining aging and cognitive disorders and have been improved by either cognitive or physical activity interventions. As described in Section 3.3, the DSST and the HVLT are the two cognitive measures upon which the cognition outcome in LIFE is powered and will be assessed in the following manner:

1. **Digit Symbol Substitution Test (DSST)**[127] is a measure of attention and perceptual speed[128] in which participants are given a series of numbered symbols and then asked to draw the appropriate symbols below a list of random numbers. The score is the number of correctly made matches in 2 minutes (120 seconds).
2. **The Hopkins Verbal Learning Test (HVLT)**[129] is a 12-item list learning and memory test designed for brief, easy administration with proven participant tolerability in a variety of populations. The HVLT requires the participant to listen to a list of 12 words and repeat as many as possible. The task is repeated twice, for a total of three trials. Approximately 20 minutes later, s/he is asked to recall as many words as possible. The participant is also presented a yes/no delayed recognition trial consisting of a randomized list that includes the 12 target words and 12 non-target words, six of which are drawn from the same semantic categories as the targets. Scores for immediate recall (total of three trials), delayed recall, and recognition are calculated into a summary score.

In addition:

3. **The Modified Mini-Mental Status Exam (3MSE)**[130] will be administered as a test of global cognitive function which assess a broad variety of cognitive measures. This is an expanded 100 point version of the original Folstein MMSE and has been used in large epidemiological studies and clinical trials to effectively screen for persons experiencing impaired cognition and estimate incident Mild Cognitive Impairment (MCI) or dementia (see below).

4. **The Modified Rey-Osterrieth Complex Figure (Rey-O)**[131] copy and immediate recall will be administered as a measure of visuospatial skills. The Rey-O has published normative data and there are standard qualitative scoring schemes that provide important information about the nature of underlying cognitive impairment.

The final components of the cognitive battery will be a computer-based battery of tests focused on elements of executive function:

5. **Eriksen flanker task** measures response inhibition.[132] Participants are presented with an arrow facing either right or left and are asked to press a key indicating its direction. The target displays can be neutral (no flankers), congruent (flanker arrows point in the same direction as the target arrow), or incongruent (the flanker arrows point in the opposite direction).
6. **N-Back Test** measures working memory.[133, 134] Participants see individual letters at a 2-second rate on a computer screen and are asked to indicate whether the presented letter is the same as the nth back letter, with n equal to 1 and 2.
7. **Task Switching** measures attentional flexibility.[135, 136] Participants are asked to quickly alternate between performing two different tasks, which requires executive function to reconfigure the cognitive system each time the task demands shift. They will be shown single digit numbers and asked to determine if they are odd or even, which will alternate with presentation of single letters for which they will have to indicate whether the letter is a consonant or vowel.

Participants with known dementia or who score below education-based cutoffs on the 3MSE will be excluded from the LIFE trial during the screening visit (see 4.2.1). In order to classify cognitive status on all participants enrolled at baseline, those who score–76 - 88 (English speaking non-African American) or 70-88 (African American and Spanish speakers) on the 3MSE will need an assessment of daily functioning with regard to cognitively demanding tasks. Thus, we will administer the **Functional Activities Questionnaire (FAQ)**[137] to a proxy (family member, close friend or caregiver). The FAQ is a brief, 10 item questionnaire, that has been validated for the purposes of ascertaining the impact of cognition on important daily functions. Results from the FAQ in combination with the cognitive tests will be used to classify the cognitive status of all participants at baseline.

All tests listed above will be re-administered at follow-up. At the 18 month visit the computer-based battery will be administered. At the 24 month and PIV visits, in addition to 3MSE, HVLT, DSST, and Modified Rey-O, these additional assessments will be administered to all participants: Category Fluency – Animals, Boston Naming Test (15 item), Trails A & B (See Appendix A). For participants not able to complete any aspect of the cognitive test battery at their 18 or 24 month visit, it will be administered at their 30 month visit. If an in-person assessment is not possible, a Telephone Interview for Cognition (TICS) will be obtained. If no contact with the participant is possible (severe cognitive impairment, death), a Dementia Questionnaire (DQ) will be administered to a proxy.

6.2.12. Health-Related Quality Of Life

The following key components of HRQL are assessed at baseline, year one and year two via self-administration:

1. Depressive symptoms are assessed with the 11-item version of the Center for Epidemiologic Studies Depression Scale (CES-D),[138] which queries about depressive

symptoms experienced in the previous week. Scores are transformed using the procedure recommended by Kohout et al. to make it compatible with the full 20-item instrument.[139] Total scores range from 0 to 60, with higher scores indicating more depressive symptoms.

2. Energy and fatigue level is assessed by the 6 fatigue and energy items from the Modified Exercise-induced Feeling Inventory.[140] Each item is rated on a 6-point scale, which focuses on the amount of time that individuals experienced fatigue or energy related feelings during the past week.
3. Perceived stress is assessed by the 10-item version of the Perceived Stress Scale (PSS), which assesses perceived global stress on a 5-point Likert scale.[141] The items were designed to tap how unpredictable, uncontrollable, and overloaded respondents find their lives.[142]

6.2.13. Quality of Well-Being Scale (QWB-SA)

The self-administered version of the Quality of Well-Being Scale (QWB-SA) is used to assess general quality of life and for the subsequent cost-utility analyses. Participants are asked to complete the questionnaire (described below) at home prior to the second screening visit and all subsequent visits. These forms are reviewed for completeness during the relevant screening/clinic visit. The QWB is a comprehensive measure of health-related quality of life that assesses health symptoms and functioning. The observed level of function and the subjective symptomatic complaints are then weighted by preference, or utility, on a scale that ranges from 0 (dead) to 1.0 (optimum function). The weights were obtained from independent samples of judges who rated the desirability of observable health states. Several studies have shown that the weights do not vary as a function of demographic variables, including race, income, and gender.

The QWB-SA takes about 10 minutes to complete. The assessment covers an extensive list of symptoms including both acute and chronic conditions and psychological well-being is well represented. The questionnaire asks about symptoms and functioning over the previous 3 days, minimizing recall bias, and providing a "point in time" expression of health. The measure has been selected for several multisite NIH clinical trials, including the National Emphysema Treatment Trial (NETT),[143] the Diabetes Prevention Program (DPP), and portions of the Prostate, Lung, Colorectal, and Ovarian screening trial (PLCO). In addition, the QWB has been used in a variety of clinical studies for a range of medical and surgical conditions that include COPD,[144] AIDS,[145] cystic fibrosis,[146, 147] diabetes mellitus,[148] atrial fibrillation,[149] lung transplantation,[150] arthritis,[151] cancer,[152, 153] schizophrenia,[154] and many other conditions.[155]

6.2.14. Health Care Utilization

Health care utilization is assessed at the second screening visit and all subsequent visits using a self-administered questionnaire developed at the University of California San Diego. The measure consists of 12 questions that ask about the frequency of various types of health care utilization over the previous 6 months. The questions ask about utilization of hospital days, emergency care, urgent care, primary care, telephone calls, prescriptions, and medical equipment. Health care costs are calculated by multiplying the frequency of each service by the prevailing community charge. The measure has been validated in a clinical trial of patients with chronic obstructive pulmonary disease.[156]

6.2.15. Biological Specimen Sampling and Storage

Blood samples for future assessment of biomarkers in ancillary studies are collected in the early morning, after a 12-hour fast at baseline, 6-month (only at

Northwestern University, Tufts University, University of Pittsburgh, and Wake Forest University), 12-month, and 24-month assessment visits. Blood (57.5-69.5 ml per visit) is collected via venipuncture into plain, serum-separation, EDTA-treated, heparin-treated, and citrate-treated vacutainers by a trained phlebotomist. **DNA** to be used for later genetic analyses is extracted from leukocytes collected in the EDTA-treated vacutainers at either the 1 year or 2 year follow-up visits. The participation in DNA studies is optional. Samples are stored at the individual field centers or at the central repository at the University of Florida. Blood for gene expression studies will be drawn into specially designed tubes for RNA isolation (Tempus tubes, Applied Biosystems) at baseline, 6-month (only at Northwestern University, Tufts University, University of Pittsburgh, and Wake Forest University) and the 12-month and 24-month assessment visits. This tube will be shipped to Dr. Chupp's lab at Yale University for processing and storage. The participation in the RNA studies is also optional. Part of the baseline (SV2) samples are sent to a central diagnostic testing laboratory for the assessment of cardiovascular risk and overall health risk: a lipid panel (triglycerides, total cholesterol, HDL-cholesterol, LDL-cholesterol (calculated)), cholesterol/HDL Ratio (calculated), a complete blood cell count (CBC) and a comprehensive metabolic panel with glomerular filtration rate, estimated (eGFR): albumin, albumin/globulin ratio (calculated), alkaline phosphatase, ALT, AST, BUN/creatinine ratio (calculated), calcium, carbon dioxide, chloride, creatinine with GFR estimated, globulin (calculated), glucose, potassium, sodium, total bilirubin, total protein, urea nitrogen).

Urine samples for future assessment of biomarkers in ancillary studies are collected at baseline, 12-month, and 24-month assessment visits. The urine is collected via clean-catch midstream technique. Participants are instructed on the technique and escorted to the restroom where they will provide at least 10 cc of urine into a urine collection cup. Urine samples are centrifuged at room temperature at 1000 *g* to remove cellular debris. Supernatants are aliquoted into 1 ml samples, labeled, and stored at -80 Degrees C. Specimens are either stored locally at each clinical center, or at a central repository. Urine sampling/storage is optional and not required for participation in the study.

6.2.16. Sleep-Wake Disturbances

As a tertiary outcome, Sleep-Wake Disturbances will be evaluated with four validated instruments: Epworth Sleepiness Scale (ESS), which assesses perceived daytime drowsiness during several different activities [157], Insomnia Severity Index (ISI), which assesses insomnia symptoms [158], Pittsburgh Sleep Quality Index (PSQI), which assesses perceived sleep quality and disturbances,[159] and Berlin Questionnaire (BQ), which assesses the clinical risk for obstructive sleep apnea.[160] In addition to these instruments, two additional questions will be administered regarding napping behavior and the use of caffeine and energy drinks. These sleep instruments and the additional two questions will be completed at SV2 and at the 6, 18 and 30 month clinic visits.

6.2.17. Pulmonary Questionnaires

A modified version of the ATS-DLD-78-A questionnaire assesses respiratory symptoms, prior cardiopulmonary illnesses, occupational history, and smoking exposure (total of 11 questions, each having subsections) [ATS-DLD-78-A Adult Dyspnea Questionnaire. at http://www.cdc.gov/niosh/respire.html]. To reduce participant burden and overlap with other LIFE instruments, the modified version omits demographic information, cardiovascular history and family history of pulmonary illnesses. This modified instrument will require about 20 minutes to administer. At follow-up, the ATS-DLD-78-A will be further modified to include only respiratory symptoms, and smoking status (10 minutes).

In addition, the Borg index for Dyspnea,[161] which assesses dyspnea, will be administered immediately after the 400 m walk each of the follow-up clinic visits.

6.2.18. Ventilatory Capacity

Ventilatory capacity will be assessed by spirometry and maximal inspiratory pressure (MIP). Spirometry will be recorded by the EasyOne™ PLUS spirometer to record average FEV6 and FEV1 over three reproducible trials. MIP will be recorded by a pressure gauge fitted with a disposable cardboard mouthpiece. The assessment of MIP will allow us to evaluate respiratory muscle weakness as a cause of reduced ventilatory capacity and as a risk factor for subsequent hospitalization (e.g. pneumonia). Older persons who have impaired skeletal muscle function may also have respiratory muscle weakness. Ventilatory capacity will be assessed at SV2 and at the 6, 18 and 30 month clinic visits.

6.2.19. Cardiovascular Questionnaires

The San Diego claudication questionnaire will be used to assess the claudicating and atypical leg pain with exertion. It will be incorporated into the medical history questionnaire and administered at baseline SV2 examination and 30 month clinic visits.

6.2.20. Ankle-Brachial Index (ABI)

Ankle brachial index is used to assess obstruction to arterial flow in the legs, usually due to atherosclerosis in the legs. It can limit ability to engage in walking and symptoms of leg pain can improve in response to an exercise intervention. Systolic blood pressure will be measured in duplicate in the arms and legs with a Doppler probe at the baseline SV2 examination and 30 month clinic visits. In addition, ABI will be obtained at closeout only if the measures were missed at 30 month follow-up or if the 30 month follow-up visit occurred more than one year to the closeout visit.

6.2.21. Social Cohesion and Trust Scale and Environmental Walkability Scale (NEWS-A)

To evaluate the potential impacts of perceptions of neighborhood cohesion and trust on participants' attempts to become more physically active, the 6-item social cohesion and trust scale will be collected on all participants enrolled in the study .[162] The scale, collected along with the abbreviated version of the Neighborhood Environmental Walkability Scale (NEWS-A),[163] will be given to all participants at randomization to complete at home and return at the first introductory intervention session. Each field center will send the completed forms to the DMAQC for computer data entry. The social cohesion and trust scale and the NEWS-A perceived environment scale will be evaluated as potential moderators of physical activity intervention effects.

6.2.22 Cancer Follow-up Form

This questionnaire is administered among participants who reported a history of cancer, other than a minor skin cancer prior to randomization. The objective is to collect self-reported information about the date of cancer diagnosis and type of treatment received.

6.2.23 Cardiovascular Medical History Baseline Update Form

This questionnaire is administered to all participants. The objective is to obtain more complete self-reported information from participants about their history of

cardiovascular disease, including date of diagnosis and/or treatment, prior to randomization.

6.2.24 Extension Study Participant Interest Survey

We will collect information to evaluate participant interest in a potential extension study.

6.2.25 Six-Item Screener

The six-item screener (Callahan)[208] will be administered to participants during the follow-up clinic visits at which the 3MSE is not completed to determine when a proxy informant should be used to ascertain the non-cognitive study outcomes. The six-item screener is a brief and reliable instrument for identifying participants with cognitive impairment and its diagnostic properties are comparable to those of the full Folstein Mini-Mental Status Examination (MMSE). The six-item screener can be administered by telephone or face-to-face interview and is easily scored by a simple summation of errors. The six items include the three-item recall (apple, table, penny) and three-item temporal orientation (day of the week, month, year) from the MMSE (and 3MSE).

6.3. Intentionally Blank

6.4. Randomization

6.4.1. Final Eligibility Assessment

Data related to eligibility and key measures must be entered prior to randomization. A computerized check is performed to confirm that all required elements are entered and are within range prior to randomization. If the eligibility check is not successful (i.e., it shows the participant as ineligible), staff in the clinic confirms that all required data were entered correctly, correct any omissions or errors in the database, and re-initiate the eligibility check. Any corrections that are made to the eligibility screens after the eligibility check is run are documented in the system and reviewed periodically by the Data Management, Analysis and Quality Control Center to ensure compliance with the study protocol. Eligibility is dependent on screening data being collected within a set timeframe: all screening data are to be collected within 60 days (i.e., the time between the date of the telephone screening interview and the date of randomization cannot exceed 60 days) and key clinical and performance measures (400-meter walk; weight) are to be collected within 45 days of randomization. The computerized eligibility check does not permit randomization if the dates for these data are outside of these ranges. If a screenee is ineligible, staff determines whether this may be a temporary condition (e.g., blood pressure out of range or too young of age) and discuss this with the participant. Re-screening can be conducted at a later date in such situations.

The allowable time from the date of randomization to the date of the first individual intervention is two months. Randomizations are timed at the clinic sites to allow these deadlines to be met. The Data Management, Analysis and Quality Control Center monitors these activities and provide regular reports to the study leadership.

6.4.2. Randomization Algorithm

Each eligible participant is randomized to one of the two arms of the clinical trial (physical activity intervention or successful aging program intervention) according to a variable block-length algorithm that is controlled by the Data Management, Analysis and Quality Control Center. This approach provides a high probability of balance between intervention assignments and makes anticipation of assignments difficult. Randomization

is stratified by field center to ensure nearly equal sample sizes for the two intervention groups within each center. This is necessary because the cohorts assembled by the centers differ due to local population characteristics and recruitment plans. Randomization is also stratified on gender to ensure nearly equal sample sizes for the two intervention groups within gender. Randomization assignment is made using a web-based randomization system that is part of the study data management system.

6.4.3. Masking or Blinding

Masking, which is used synonymously with the term “blinding,” refers to structured attempts to limit the disclosure of study data and participant status to as few persons (both study personnel and participants) as possible. It is generally recommended that access to all types of study data be limited. This includes access to clinic and laboratory measurements, intervention group assignment, and measures of adherence to interventions. Many examples exist in the medical literature to demonstrate that knowledge of some aspects of a participant’s status can subjectively lead to differences in how data are collected and interpreted. The assessment team is blinded to the intervention assignment.

7. Interventions
7.1. Intervention Theory and Goals
7.1.1. Intervention Theory

The intervention is based upon a **social cognitive model of acquisition and maintenance of health behaviors**. The social cognitive approach views behavior (including health behavior) as being acquired and maintained through a complex set of behavioral, cognitive, physiological and environmental conditions. Social cognitive intervention strategies are found in a number of studies to be effective with older as well as younger adults, and with programs aimed at physical activity as well as with other forms of health behavior change. Concepts from social cognitive theory are combined with strategies derived from recent applications of the Transtheoretical Model to the area of physical activity (e.g., consciousness raising and other cognitive approaches in the preparation and action phases early in the program; reinforcement, management and related behavioral approaches in the later phase of the program). These are applied on an "as needed basis" in administering the LIFE physical activity intervention using a tailored, social problem solving approach.

7.1.2. Goals of the Intervention Arms

Participants are randomized to the **physical activity (PA)** intervention or to the **successful aging program (SA)**. The physical activity intervention is of moderate intensity and consists of aerobic, strength, flexibility, and balance training with a target duration of 150 minutes per week. However, goals are individualized based on each participant's level of physical fitness and can be modified in response to illness, injury, or physical symptoms. Based on our experience, these interventions can be successfully delivered to older individuals, including frail persons, and can result in sustained participation rates and improved physical function.

The purpose of the successful aging group is to control for general levels of staff and participant time and attention, in addition to general secular and seasonal effects that could influence the outcomes of interest.

7.2. Physical Activity Intervention

The physical activity intervention includes aerobic, strength, flexibility, and balance training. **Walking** is the primary mode of physical activity for preventing/postponing the outcome of major mobility disability, given its widespread popularity and ease of administration across a broad segment of the older adult population.[77, 92] Other forms of endurance activity (e.g., stationary cycling) are, however, utilized on a limited basis when regular walking is contraindicated either medically or behaviorally. Each session is preceded by a brief warm-up and followed by a brief cool-down period. In light of current clinical guidelines, participants are instructed to complete **flexibility** physical activities following each bout of walking. Moreover, two times each week, following a bout of walking, participants are instructed during the initial phase of the program to complete a 10-minute routine that focuses on **strengthening exercises** for lower extremity muscle groups by using variable weight ankle weights. This will be followed by a brief lower extremity stretching routine.

Supplementary instructional materials are supplied to participants in this group, to reinforce the strength training occurring during center-based instruction, so that it can be generalized to the home environment. **Balance training**[52] is introduced during the adoption phase of the program as a complement to the aerobic and strength components. In addition, the intervention involves encouraging participants to increase all forms of physical activity throughout the day. This may include activities such as leisure sports, gardening, use of stairs as opposed to escalators, and leisurely walks with friends.

Intensity of training. The participants are introduced to the activities of the physical activity intervention in a structured way such that they begin with **lighter intensity and gradually increase intensity** over the first 2-3 weeks of the intervention. LIFE promotes walking for exercise at a **moderate intensity** and relies on **ratings of perceived exertion** as a method to regulate physical activity intensity.[94, 95] Using Borg's scale,[96] that ranges from 6 to 20, participants are asked to walk at an intensity of 13 (activity perception SOMEWHAT HARD). They are discouraged from exercising at levels that approach or exceed 15 (HARD) or drop to a rating of 11 (FAIRLY LIGHT) or below. Lower extremity strengthening exercises are performed (2 sets of 10 repetitions) at an intensity of 15 to 16 using Borg's scale for the strength training component of the program.

7.2.1 Contact Mode and Frequency

The physical activity intervention consists of a general weekly walking goal of 150 minutes. This is consistent with the public health message from the 2008 Physical Activity Guidelines for Americans report that moderate physical activity should be performed for 30 minutes on most if not all days of the week (150-210 total minutes). This goal is **approached in a progressive manner** across the first 3 months of the trial. There are multiple ways that the goal can be achieved, based on the physical abilities and constraints of each participant.[52, 97] In light of the heterogeneity of the target population (with respect to physical capabilities and health status), this study will specifically define the variability in participants' ability to reach this weekly target, to estimate the dose-response relationship between incremental increases in weekly physical activity and changes in the primary and secondary outcomes, and to better specify the level of ongoing behavioral instruction needed to achieve such changes.

Table 7.2.1. Intervention staff contacts for physical activity group

Week	Center-Based Physical Activity	Home-Based Physical Activity
Adoption: weeks 1-52	2 times each week	1 time/week (weeks 1-4)<br>2 times/week (weeks 4-8)<br>Up to 3-4 times/week (weeks 8-52)
Maintenance: weeks 53 – end	2 times each week	Up to 3-4 times/week

7.2.2. Participant assessment at baseline

As undertaken in other programs with older adults,[164-166] each participant randomized to the physical activity group will receive a 45-minute individualized, face-to-face **introductory session**, during which time the program is described, questions are answered, and results from each individual's baseline assessment is utilized to tailor the program with respect to physical activity progression, as well as to optimize safety and participation.

When participants first enter the physical activity intervention, their **demographic and contact information is** entered into a structured data window that is part of the computerized tracking system. In addition, the computer prompts interventionists to complete **session cards** for participants at each scheduled visit. These session cards include information on attendance, the specific goals for the physical activity prescription, and the amount of physical activity completed during the visit. In addition, on a weekly basis, interventionists enter the total number of minutes of physical activity performed each day of the previous week (recorded in logs). In this manner, LIFE can track and promote physical activity that is occurring both at the center and off site.

7.2.3. Intensive Contact Phase

The PA intervention will comprise an adoption and a maintenance phase:

7.2.4. Adoption phase (weeks 1-52)

Two **center-based** exercise instruction sessions per week will be conducted in a supervised setting. These sessions will be used to initiate the walking program and to introduce participants to the strength, stretching, and balance portions of the program in a safe and effective manner. The supervised setting will allow instructors to better tailor the program to individual needs and abilities early on, so as to prevent early dropout and to facilitate the building of self-efficacy and support, which have been found to be key to long-term physical activity maintenance.[167] These exercise sessions will involve 40-60 mins of exercise instruction. For those participants who miss 2 consecutive exercise class sessions, without informing the exercise staff of their absence, exercise staff will call the participant to problem-solve ways to get the participant back to class.

In addition, the center-based sessions will be supplemented, in a progressive fashion, by **home-based** exercises as a means of promoting physical activity in multiple settings to aid behavioral generalization and long-term adherence. This has been found to be a key feature of sustained physical activity participation among older as well as younger adults.[92] Appropriate community based exercise facilities (e.g., YMCAs; senior centers) will be identified for those persons preferring to undertake center-based activities on a more frequent basis throughout the week.

7.2.5. Maintenance phase (week 53 through the end of the trial): The Maintenance phase will consist of:

- Continued twice-per-week **center-based** group exercise sessions offered to each participant.
- Progression of home-based physical activity to 3-4 times per week.
- Quarterly project **newsletters**, used to promote ongoing support and participation and to provide ongoing information related to physical exercise participation and adherence.

7.2.6. Educational Modules

The participants receive all of the written material provided from the health education ‘Healthy Aging Workshop’ modules provided to the participants in that arm of the study.

7.3. Successful Aging Program Intervention

7.3.1. Contact Mode and Frequency

The successful aging program arm meets in small groups (approximately 25 participants per group), one time each week for the first 26 weeks of the intervention. From week 27 on, successful aging groups with be offered two times per month with required participation at least once per month. Telephone calls are made after missed visits to problem-solve barriers to attendance and to encourage regular participation. As undertaken in LIFE-P, those participants randomized into the Successful Aging Program will receive an individual 45 minute face to face introductory session, by a health educator, during which time the program is described and questions are answered.

Table 7.2.2. Intervention staff contacts for the successful aging health education group

Week	Center-Based Workshop	Telephone Contact
Adoption Week 1-26	1 time each week	Intervention staff contact made after one missed session to problem-solve around barriers to attendance
Maintenance Week 27 – end	Offered 2 times per month with ongoing participation required at least monthly	Intervention staff contact if participant misses first session of the month

7.3.2. General Content and Structure of Intervention Modules

The Successful Aging Program group is based on a **successful aging workshop series.** Participants receive information on a variety of topics of relevance to older adults (e.g., how to effectively negotiate the health care system, how to travel safely, recommended preventive services and screenings at different ages, where to go for reliable health information, etc.). The program includes an experiential component, in which participants learn how to actively 'take charge' of their health in seeking out appropriate medical information and services. In addition, to these educational offering, a short instructor led program (5-10 minutes) of **upper extremity stretching exercises** or some relaxation techniques are performed during each class. To reduce boredom, additional upper extremity stretches will be added during the study and will be included in the MOP when they are introduced.

The rationale for this "placebo exercise" activity is that it helps foster adherence to this arm of the study and increases the perceived benefit of the Successful Aging workshop series to the participants without directly affecting the study outcomes.

7.4. Strategies for Keeping Participants Involved in the Intervention

7.4.1. Adherence and Monitoring

Adherence to all scheduled intervention contacts is recorded by interventionists into a tracking system. Overall and site specific reports on adherence are posted on the web and periodically updated. In addition, the Lifestyle Resource Core monitors these adherence reports and provides monthly feedback to each site.

7.4.2. Strategies for Promoting Adherence in the Physical Activity Group

During the **intervention** the primary behavioral techniques include:

1) **Personalized feedback and setting of individualized goals**, based on functional testing that occurs during the initial center-based physical activity session, and based on determination of an individualized physical activity program that is tailored to physical performance test results. Additional regular feedback on level of activity is obtained via use of a pedometer.[169, 170]
2) **Specific structuring of expectations** concerning the effects of physical activity, to ensure that participants' expectations are reasonable and realistic.
3) **Consciousness raising** and similar experiential processes related to the problems of under-activity, and the benefits of adopting a more active, heart-healthy lifestyle (e.g., self-reevaluation processes)[171]
4) The use of a staff-participant **contract** to clarify goals and increase initial participant commitment to the goals. This contract, read and signed by the participant and staff member following random assignment to the physical activity group, restates the responsibilities of both the participant and project staff with respect to the study, and is used to note the specifics of the first several weeks of the intervention (e.g., days, location).[172]
5) Frequent **individual instruction** (via telephone and through the scheduled center-based sessions), support, goal-setting, and feedback with a trained staff person throughout the intervention period, tailored to facilitate each individual's ongoing

behavioral participation as well as performance level.
6) Provision of all center-based **exercise equipment** (e.g., exercycles), as deemed appropriate.
7) Distribution of easy-to-read **written materials** to prompt regular and appropriate participation in the physical activity programs.
8) Instructions to maintain a simple daily **activity calendar/log**, which details the intensity (rating of perceived exertion),[96] duration, frequency of activities being undertaken, and the number of steps recorded on the pedometer (in some participants if found to be useful). Such calendars have been used extensively in previous studies of older adults and have been found to be brief and easy to complete by older men and women across periods spanning 12 to 24 months.[164-166, 168, 173] To reduce participant burden and costs associated with mailing physical activity logs back to the clinic, participants record their physical activity behaviors on a simple, easy to use magnetic calendar, which is affixed to the refrigerator. Participants subsequently report this recorded information to clinic staff during intervention visits.
9) Instruction in the use of **visual prompts** to encourage and reinforce successful change.[92]
10) Monitoring of **immediate disincentives** to adherence (e.g., discomfort, perceived inconvenience) on the activity logs/calendar, and active brainstorming with staff members via telephone to minimize them.
11) Introduction to **relapse prevention** strategies via telephone, mail, and setting-based contacts by identifying and planning for high-risk situations such as illness, in which early relapse from physical activity programs is likely. This also includes instruction in problem-solving methods and skills to help individuals develop and apply strategies, so that they may overcome barriers to attaining their physical activity goals.

During the **maintenance phase,** the primary behavioral techniques include:

1) **Regular updating of behavioral and performance-based goals,** to ensure that goals remain realistic yet challenging.
2) Continued **logging** of target behaviors.
3) Further development of plans to keep the regimen **flexible**, with respect to location, scheduling, and other issues, to accommodate preferences as well as periodic fluctuations in motivation and schedules.
4) Increased instruction in and use of **self-rewards** and other self-control, reinforcement management strategies for behavioral maintenance.[167]
5) Increased practice in the application of subject-initiated **relapse prevention and problem-solving strategies**, with relevant feedback and support provided by the intervention staff through telephone and center-based contacts.
6) Continued use of **stimulus control** strategies (e.g., visual prompts) to promote maintenance.
7) Continued receipt of **social support** via regular staff telephone, mail, and setting-based contacts.

7.4.3. Strategies to Enhance Participation Rates in The Successful Aging program

The following behavioral strategies, which have been used successfully to promote sustained participation in previously studied health education control groups,[165] parallel the behavioral strategies to be used in the physical activity group. These include the following:

During the adoption and transition phase (first 6 months) for the successful aging program group the primary behavioral techniques include:

1) **General feedback** obtained from baseline testing related to overall levels of health and functioning.

2) **Specific structuring of expectations** concerning the Successful Aging curriculum, to ensure that subjects' expectations are reasonable and realistic.
3) **Consciousness raising** and similar experiential processes related to the problems of a poor diet and other health areas (e.g., foot and eye care; medical screening), and the benefits of adopting a healthier lifestyle.
4) Establishing **concrete goals** related to attending the Successful Aging sessions and participating in that intervention throughout the one-year intervention period.
5) A staff-participant **contract** (following randomization) is used to clarify the above goals and expectations and to increase initial participant commitment to the goals. This contract, reviewed and signed by the participant and a staff member, restates the responsibilities of both the participant and project staff with respect to the study, and is used to note the specifics of the first several weeks of the successful aging program intervention (e.g., days, location).[172]
6) Distribution of easy-to-read **written materials** to prompt regular and appropriate participation in the Successful Aging program, including mailing a monthly health education newsletter.
7) All participants assigned to this group are encouraged to attend the Successful Aging Program sessions on a weekly basis, to foster **early 'buy in'** to this intervention group, and to set the stage for continued participation throughout the intervention period. During the latter portion of the initial 6-month period, participants are encouraged to actively participate in choosing topic areas that receive additional focus during the maintenance phase.
8) Similar to the physical activity group, participants assigned to the successful aging program group are encouraged to **track behavior changes** related to nutrition and other areas; they are given relevant homework assignments on an as needed basis to complete prior to the next class meeting (e.g., trying specific healthful recipes; undergoing simple pantry checks in their homes; food label reading activities).
9) Participants who miss a scheduled meeting are contacted via telephone by a study interventionist to encourage continued participation in this group and to use **problem-solving** skills to overcome potential barriers to continued participation.

During the **maintenance phase** (7th month through the end of the trial), participants in the **successful aging program** continue to receive support from study intervention staff that relates to participation in the monthly Successful Aging meetings. Those participants who miss a scheduled meeting are contacted to encourage continued participation in this group and to use problem-solving skills to overcome potential barriers to continued participation. Participants are encouraged to actively 'take charge' of their ongoing program experience, with respect to topic areas of interest, guest speakers, etc.

7.4.4. Protocol for Managing Illness/Injury and Other Health Problems

If physical activity is reported to have been suspended due to a hospitalization, injury or other health reason, the participant is asked to come to the center for re-evaluation to determine the level of physical activity for restarting, once it is determined that the health event has resolved. If the health event remains unresolved, monthly calls are made to reassess whether criteria for restarting are met, as described below.

Restarting a suspended physical activity program. Evaluation for restarting physical activity depends on the functional impact of the illness and any activity limitation prescriptions that may have been provided by the participant's health care team, including the primary care physician, surgeon, consultants, or therapists.

a. If, after the illness episode, the participant is able to leave the home and walk independently outside the home with no more assistance than a straight cane, and if there is no prescribed activity or weight bearing-limitation or therapy, reevaluation is done at the Field Center, and a new physical activity prescription begins. The same protocol as was used for the baseline program prescription and progression is used.
b. Regardless of ability to leave the home, if after an acute illness and suspension of physical activity the participant is under prescribed activity or weight bearing limitation or rehabilitative treatment, re-evaluation is made at the end of the activity limitation prescription or treatment course.
c. If the physical activity is specifically limited due to chest pain or dyspnea, physical activity is suspended and is not restarted without definitive treatment by the participant's health care provider. In some of these cases, the primary care physician may refer the participant to a medically supervised rehabilitation program. When this occurs, the intervention staff attempts to obtain information on what the participant is doing in the rehabilitation program so that this information can be added to study records.
d. If the participant remains unable to leave the home under the conditions prescribed above, and is nearing the end of a six-month assessment window, a home examination is done at the required interval to assess for study endpoints. A similar protocol is used for the control group.

Individualizing restart of physical activity after illness or injury episode. The physical activity program is adapted to the assessed level of ability. This is the same protocol as the baseline starting protocol for individualizing the start of physical activity. A special remedial program is provided for those who fall below the original starting criteria for enrollment. There may be appropriate individual variations in the resumption of physical activity during this period due to the length of the suspension and the severity of the participant's illness or injury. For this reason, all individual plans for the resumption of physical activity for a participant who has been placed on "suspended" status will be reviewed and approved by the **Lifestyle Resource Core**.

Individualizing goals when physical activity is reduced because of illness or injury. If there is an illness episode that does not meet the above criteria for suspension of the physical activity program, reduction in physical activity may still occur, and is detected by either the tracking system, observation by staff, or self-report at a center visit. Physical activity goals are re-adjusted on an individual basis. Re-assessment or need for special attention and individualization is performed at the field center. All injuries are reported to the Medical Safety Committee. Rehabilitation staff and primary care physicians may also be consulted as needed.

8. Participant Safety and Confidentiality

The study monitors the medical safety of participants. One aspect of this monitoring is to evaluate potential volunteers at screening to determine whether it is safe for them to participate in the planned intervention. Another aspect is monitoring of safety during study assessments. A third area is safety during physical activity, both supervised and unsupervised. Also, if a volunteer has a medical or surgical illness, the safety of continuing or resuming participation in interventions is ascertained by the medical staff at the local center in cooperation with the participant's primary care physician. Finally, the study monitors adverse events, assess their potential relationship to the intervention and report events to the Data Safety Monitoring Board (DSMB).

8.1. Data Safety Monitoring Board

A **Data Safety Monitoring Board (DSMB)** is established, with responsibility to monitor all aspects of the study. The **Medical Safety Committee** reports to the DSMB for issues related to participants safety.

The DSMB has the following charges:

- Review the entire study protocol and the informed consent form with regard to recruitment, randomization, intervention, subject safety, data management, plans for auditing of subject records, and quality control and analysis plans, and to identify needed modifications prior to the start of the study.
- Identifies the relevant data parameters and the format of the information to be regularly reported.
- Review data (including masked data) over the course of the trial relating to efficacy, recruitment, randomization, compliance, retention, protocol adherence, trial operating procedures, forms completion, intervention effects, gender and minority inclusion and subject safety.
- Identify problems relating to safety over the course of the study and inform study PI via written report, who, in turn, ensures that all Field Center PIs receive this report.
- Identify needs for additional data relevant to safety issues and request these data from the study investigators.
- Propose appropriate analyses and periodically review developing data on safety and endpoints.
- Make recommendations regarding recruitment, intervention effects, retention, compliance, safety issues and continuation of the study.
- Send the Program Administrator and PI written reports following each DSMB meeting. These reports may address all (blinded) issues reviewed by the DSMB. The PIs then send the DSMB report to their respective IRBs.(The study PI is responsible for sending the reports to individual site PIs, who in turn are required to distribute the report to their local IRBs.)

At any time, the DSMB may recommend discontinuation of any component/intervention group of the study for any of the following reasons:

1) Compelling evidence from this or any other study of an adverse effect of the study intervention(s) that is sufficient to override any potential benefit for the interventions to the target population.
2) Compelling evidence from this (or any other) study of a significant beneficial effect of the study intervention(s), such that its continued denial to other study group(s) would be unethical.
3) A very low probability of addressing the study goals within a feasible time frame.

The DSMB may convene an executive session at any time. The NIA makes the final decision on whether or not to accept the DSMB's recommendation about

discontinuation of any component of the study. Any serious adverse events that might be due to the study intervention are reported to the DSMB, the IRB and to the Project Office.

8.2. Medical Problems Detected During the Study Assessments

Medical problems that increase risk of study participation are assessed through structured telephone interviews and in person physical examinations during the initial subject evaluation, prior to randomization. The goal of these assessments is to detect conditions by history, such as recent major surgery, symptomatic conditions such as angina or weight bearing pain and asymptomatic conditions, such as valvular heart disease or abdominal aortic aneurysms. Such persons are excluded from further participation and are referred to their primary care physician for further care.

8.3. Safety Considerations for Study Assessments

All study assessments are done by trained and certified staff. Safety precautions are taken during the 400 m walk test by applying standardized stopping criteria. If the participant reports chest pain, tightness or pressure, significant shortness of breath or difficulty breathing, or feeling faint, lightheaded or dizzy the test is stopped. During the 400 m walk tests a defibrillator is available. Onsite staff are trained to provide basic life support and to provide immediate care when faced with medical emergencies. Also, institutional and community EMS services are activated if needed.

It is anticipated that some medical problems occur during the course of the study while some participants are in the clinic. The following is a summary of a plan of action based on level of acuity of the problem.

Emergent problems and problems that are life threatening or require life saving attention should be dealt with using the local Emergency Medical System (EMS). Clinical staff may provide basic life support as an interim measure when appropriate until EMS personnel arrive. CPR training is recommended but is not required. The study staff is responsible for notifying the participant’s family or designated contacts and the participant’s primary care provider.

Urgent medical problems and problems that require immediate attention but that do not require life saving attention are dealt with by taking measures to ensure the participant’s comfort and offering first aid, as appropriate. Disposition plans should be made with the participant, clinic staff, investigators, family, and primary care provider. The clinic staff may arrange transportation of the participant to another medical care site for definitive care. The primary care provider and family or designated contacts should always be notified.

General medical problems or those problems that require attention when feasible should be dealt with by contacting the primary care provider. The clinic staff should follow the primary care provider's directions regarding disposition and follow-up. The participant should be advised regarding the primary care provider's instructions and documentation of the problem and actions should be placed in the participant’s record on a progress note. A follow-up letter to the primary care provider documenting the problem and actions taken should be sent by clinic staff.

There are several types of alerts in LIFE. The table below describes a summary of alerts and the appropriate action.

ALERT	ACTION
Blood Pressure<br>SBP > 140mm/Hg or<br>DBP > 90mm/Hg	Clinic staff inform the participant
Blood Pressure<br>SBP > 170mm/Hg or<br>DBP > 100mm/Hg	Qualified staff should talk to participant, and encourage participant to seek additional follow-up and/or evaluation.
Resting Pulse<br>Rate > 100 or < 40 beats/min	Qualified staff should talk to participant, and encourage participant to seek additional follow-up and/or evaluation.
Mood questionnaire score >= 24 or 3MSE score below cut-points in Section 4.2.1	Qualified staff should talk to participant, and encourage participant to seek additional follow-up and/or evaluation.
ECG meets exclusion criteria.<br>Serious conduction disorder (e.g., 3rd degree heart block), uncontrolled arrhythmia, or new Q waves or ST-segment depressions (>3 mm) on ECG.	Qualified staff should talk to participant, and encourage participant to seek additional follow-up and/or evaluation.
Ankle Brachial Index <0.90	The participant will receive a letter that provides the ABI result and suggests that the participant share the letter with their physician.
No posterior tibial arterial signal present in either leg	The participant will be examined by a qualified staff member who will evaluate the participant for signs of critical limb ischemia. The qualified staff member will re-check for presence of a posterior tibial artery signal and will check for presence of a dorsalis pedis arterial signal.<br>Appropriate follow-up will be recommended. If critical limb ischemia is determined to be present, the study physician will be notified.
Forced Expiratory Volume in 1-Second (FEV1) <80% Predicted but >50%<br>Predicted.	The participant will receive a letter that provides the spirometry result and suggests that the participant share the letter with their physician.
FEV1 < 50% Predicted	The participant will be examined by a qualified staff member who will evaluate the patient for signs of respiratory distress (i.e., severe shortness of breath).
Serum glucose < 60 during intervention session in diabetic on hypoglycemic agent	Intervention staff provide immediate care such as offering beverage with sugar and advise participant to contact PCP regarding adjustment of hypoglycemia medication

Alert criteria for blood tests

Test	Value out of reference range:<br><br>Participant should be notified at a routine visit or within two weeks by a qualified staff member that the value is out of the normal range for all adults but may or may not be medically important and could be discussed with PCP.	Value may be medically significant<br><br>Participant should be notified at a routine visit or within two weeks by a qualified staff member that the value is potentially medically significant and strongly recommend that it be discussed with PCP	Value requires immediate notification<br><br>A qualified staff member should contact the participant within one working day and request permission to contact a PCP. Assuming that the participant has normal vital signs and is feeling well, there is no need to invoke emergency medical systems.
Triglycerides	>199		
Total cholesterol	>199		
HDL	<40		
Hemoglobin	M <13 or > 16<br>F <12 or > 15	M < 12 or > 16.5<br>F < 11 or > 16	< 8 or > 18
WBC count	< 4000 or >11000	< 3000 or > 12000	< 2000 or > 20000
Platelet count	< 130000 or > 400000	< 100000 or > 500000	< 30000 or > 1000000
Sodium	< 135 or > 146	< 130 or > 155	< 125 or > 160
Potassium	< 3.5 or > 5.3	< 3.0 or > 5.7	< 2.6 or > 6.2
Calcium	<8.5 or > 10.3	< 8.0 or > 11.5	< 7.0 or > 13.0
Glucose	< 70 or > 125	< 60 or > 140	< 50 or > 400
BUN	> 30	> 40	> 80
Creatinine	> 1.4	M >2.0<br>F > 1.6	> 3.5
Albumin	< 3.5	< 3.0	

8.4. Safety Considerations for the Physical Activity Intervention

Appropriately designed and implemented physical activity interventions have been shown to be safe and efficacious in older adults.[35, 52, 174] The literature on physical activity training in the frail elderly in nursing homes contains no reports to date of serious cardiovascular incidents, sudden death, myocardial infarction, or exacerbation of metabolic control or hypertension.[175] Also, LIFE researchers have conducted over 26,000 assessments of maximal dynamic strength without one single cardiovascular event.[176] A recent review concluded that an appropriately prescribed resistance physical activity program is a safe form of physical activity for the majority of the population and is associated with minimal risk of cardiovascular events, even in those with previous myocardial infarction or chronic congestive heart failure.[177]

8.4.1. Pre-Physical Activity Safety Screening

To maximize the **participants' safety** we follow a standardized screening protocol (Figure 8.4.1.). Accordingly, all potential participants undergo **screening for cardiovascular and other major diseases** by means of a health questionnaire, medication inventory, ECG and physical exam, which are initially collected and reviewed by a qualified health professional such as a nurse practitioner or physician assistant. In cases where there are no potential alert values on any of the forms and no unexpected or unusual symptoms or conditions, the qualified health professional may approve performance of the 400 meter walk. In all other cases, the study physician must review and approve the participant for performance of the 400 meter walk. Those with overt cardiovascular diseases (or other severe diseases) that meet the exclusion criteria as determined by the study physician are excluded. Prior to randomization of a potential participant, all medical information must be reviewed by a study physician, who is ultimately responsible for determining study eligibility and approving randomization. Next, otherwise eligible persons undergo the **400 m walk** test. According to a protocol to evaluate cardiovascular reserve similar to the one suggested by Gill et al.,[178] persons who develop chest pain or substantial shortness of breath during the 400 m walk test are also excluded. Those who are not excluded are randomized to the physical activity intervention group or to the successful aging program.

Figure 8.4.1. Algorithm for screening morbid conditions

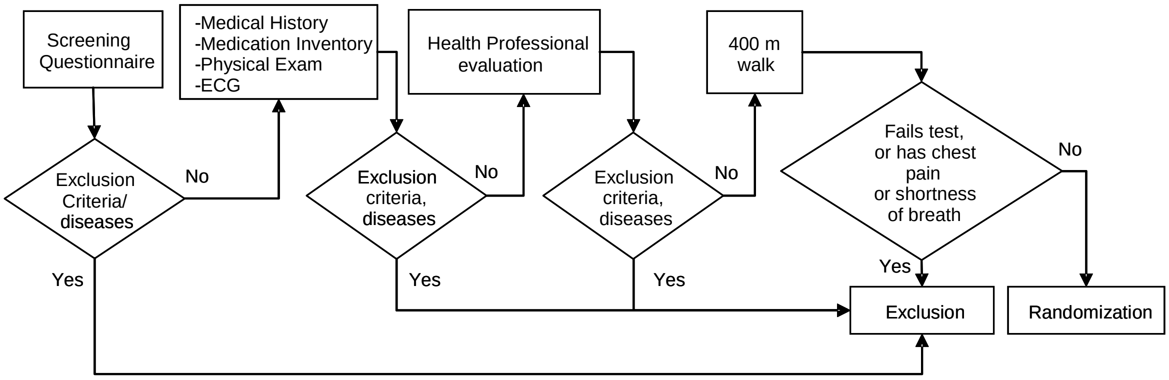

Participants do not undergo physical activity stress testing. This decision is based on the following considerations:

- The **recommendations** published by Gill et al.[178] advised that a screening protocol based on a simple cardiovascular reserve test, similar to the one described above is more suitable for screening older adults than a protocol based on stress physical activity testing.
- The American Heart Association (AHA) and the American College Sports Medicine (ACSM) **joint position statement** advised that "apparently healthy persons of all ages and asymptomatic persons at increased risk may participate in moderate-intensity physical activity without first undergoing a medical examination or a medically supervised, symptom-limited physical activity test."[179]
- The **AHA Scientific Statement** on Exercise Standards for Testing and Training by Fletcher et al., advised that "for older, apparently healthy persons desiring to participate in a low to-moderate intensity activity such as walking, an exercise test may not be required", and that "the role of exercise testing among the elderly (>75 years) as a guide to identifying the high-risk patient for primary prevention requires further study".[94]
- The majority of older persons (>75%) are **unable to satisfactorily complete** a treadmill exercise test,[180] which makes its utility as a screening tool in the elderly population questionable.
- Older persons have a **high prevalence of ECG abnormalities**,[181] which diminish the diagnostic accuracy of treadmill exercise testing.[182]
- Participants with potential cardiac contraindications to the physical activity program are identified and excluded by means of the **screening** process described above.
- Physical activity of **moderate intensity** is conducted in a **supervised environment**.
- A maximal or near maximal exercise test on a treadmill is an **unpleasant, if not frightening experience**, for sedentary and unfit adults (unpublished data from WFUHS and Cooper Institute). Requiring an exercise stress test may deter older persons from participating in the trial.
- Regular exercise and physical activity **may actually reduce the overall risk of MI and death** among older persons,[183, 184] possibly through improvements in cardiac risk factors and overall fitness.[95]

In summary, exercise stress testing provides little additional information, is not necessary to protect the safety of participants, and is disliked by sedentary and unfit participants. The physical activity intervention protocol also requires that the center-based sessions at the beginning of the study include careful monitoring of cardiac and other signs and symptoms by trained staff.

8.4.2. Safety Measures During Physical Activity

Center based interventions are conducted at a central location and all sessions are conducted and supervised by **trained interventionists,** who monitor potential adverse experiences and symptoms. During the physical activity sessions a defibrillator and on-site trained staff are available to deal with medical emergencies. Also, institutional and community EMS services are activated if needed. As indicated previously, participants are taught the importance and proper method of **warming-up** prior to and **cooling-down** following structured activity sessions. If at any point during a physical activity session, participants develop chest pain, shortness of breath, or dizziness, they are instructed to rest and to contact the center and their physicians if these symptoms persist or recur with further physical activity. The implementation of the physical activity sessions is consistent with the recommendations published by Fletcher et al. for older adults who may have

stable cardiovascular disease.[94]

Blood pressure and heart rate are monitored before and after the walking activity at each center-based intervention session. Blood pressure and heart rate are measured during the walking at each center based session in participants who had experienced any of the following at a previous physical activity session:

- Resting blood pressure systolic $\geq$ 200 mm Hg or diastolic $\geq$ 100 mm Hg
- Decrease in systolic blood pressure ≥20 mm Hg following the activity
- Increase in systolic blood pressure to ≥250 mm Hg or in diastolic blood pressure ≥115 mm Hg following the activity
- Resting heart rate $\geq$120 beats/min or $\leq$ 45 beats/min
- Increase in heart rate ≥90% of age predicted maximum
- Unusual or severe shortness of breath
- Chest pain or discomfort, or heartburn
- Palpitations
- Light headedness, dizziness or feeling about to faint
- A physical activity session had to be discontinued because of other symptoms, excluding musculoskeletal symptoms (e.g., knees, ankles, hips), reported by the participant.

If any of the above occurs, the individuals are instructed to seek their physician's permission before continuing with the physical activity program.
Very few persons are expected to drop out for this reason based on previous experience and cardiac-based exclusion criteria.

Procedures to minimize discomfort include **warm-up and cool-down activities** that include light walking or cycling and flexibility exercises. The participants are also introduced to the intervention activities in a structured way, such that they begin with **lighter resistance and gradually increase** over the course of the first 2-3 weeks of the intervention. During the intervention visits, participants are **supervised at all times and instructed** on correct physical activity techniques. Participants are instructed to talk with the interventionists about any muscle soreness.

If for any reason the participant reports an injury, chest pain, shortness of breath, or dizziness, they are referred to their doctor, or the study clinician calls the doctor or other health care provider. The participating institutions are in compliance with NIH policies regarding physical injuries resulting from experimentation of human subjects. Trained technicians administer all tests, with an emphasis on the well being of the participant. In addition, specific criteria for **suspending or stopping physical activity** are developed to adjust the program for **intercurrent illness**.

8.5. Adverse Events

Serious adverse events in LIFE are defined to include: death, a life-threatening adverse experience, inpatient hospitalization or prolongation of existing hospitalization, a persistent or significant disability/incapacity, or a clinically significant laboratory or clinical test result. Important medical events that may not result in death, be life-threatening, or require hospitalization may be considered serious adverse experiences if they might jeopardize the participant or might require medical or surgical intervention to prevent one of the outcomes in the definition. An example of this in LIFE is an injurious fall resulting in a fracture that occurred during walking for physical activity.

For LIFE purposes, an **adverse event or experience** is defined as any health-related unfavorable or unintended medical occurrence that happens during the process of screening or after randomization. Certain adverse events may be protocol-defined outcomes (serious fall injury). Minor adverse events are defined as conditions that may be

unpleasant and bothersome to the participant, such as sore muscles, but that do not require discontinuing the study intervention or components of the intervention. Examples of minor adverse events include but are not limited to the following: anxiety, fatigue, decreased appetite, insomnia, dizziness, muscle or joint stiffness, muscle strain or soreness, ankle or knee pain, foot pain, and other minor symptoms that may have restricted the participant’s usual activities for at least ½ day like a head cold, flu or allergy problems. Minor adverse events should be reported on an annual basis to each site’s own IRB.

Potential adverse events for study related activities and interventions are explained to each participant by trained study personnel during the informed consent process. Each participant is instructed to report the occurrence of an adverse event at scheduled data collection times (scheduled clinical exams or phone interviews). Participants also have access to study clinic personnel at other times to report serious adverse events or concerns about the safety of participating in the LIFE Study.

Expected serious adverse events related to the exercise intervention include in rare instances, heart attack, stroke, and death. Cardiovascular events are assessed using standard protocol measures including ECGs. When a cardiovascular event has occurred, a study physician decides whether it is permissible for the participant to continue interventions. If the LIFE interventions are discontinued for safety reasons, they may be resumed after consultation with the participant’s primary care physician.

Serious fall injuries and fractures are assessed using standard protocol measures, including radiographs and hospital records. When a serious fall injury or fracture occurs, a study physician decides whether it is permissible for the participant to continue interventions. If the LIFE interventions are discontinued for safety reasons, they may be resumed after consultation with the participant’s primary care physician.

In the LIFE safety monitoring system, participants who report adverse events to any staff person at any time are referred to unmasked medical staff responsible for identifying, recording, and managing these events. Safety-related events are reported in a timely fashion as required by the Data and Safety Monitoring Board and the IRBs responsible for the study. Interventionists and other staff reporting or managing adverse events for safety purposes do not at any time communicate information regarding these events to study assessment personnel.

LIFE maintains an event outcome database that is completely separate and distinct from the safety monitoring system for the intervention group. This is necessary because many of the LIFE staff members are not masked to intervention assignment, and it is critical that the identification and reporting of serious adverse events for safety reasons not bias the study’s collection of outcome data. Thus, for outcome purposes, all LIFE participants are systematically queried at clinic visits or on clinic phone calls scheduled according to the protocol to capture outcome data on study outcomes, medical events, or adverse experiences. This separate outcome database contains solely those adverse events that are reported through these regularly scheduled event interviews conducted by designated outcome assessment staff who are masked to intervention assignment.

All deaths shall be reported within 48 hours to both DSMB and NIA. All Serious Adverse Events shall be reported in a summary format by the blinded treatment arm (A and B) to the DSMB and NIA monthly.

8.6. Confidentiality

The information below relates to all collaborating performance sites for the study. Data are used only in aggregate and no identifying characteristics of individuals are published or presented. Results of testing are sent to participant's private physicians if

participants agree to this. Alert values for all medically relevant procedures (e.g., ECGs) are developed, and a system is in place to alert study physicians and participants' private physicians, depending on the urgency of the values.

Confidentiality of data is maintained by using research identification numbers that uniquely identify each individual. Safeguards are established to ensure the security and privacy of participants' study records. The information collected from participants in this study has a low potential for abuse, since the data do not address sensitive issues. Nevertheless, appropriate measures are taken to prevent unauthorized use of study information. The research ID number is used. The research records are kept in a locked room in the Field Center. The files matching participants' names and demographic information with research ID numbers are kept in a separate room and are stored in a locked file that uses a different key from that of all other files. Only study personnel have access to these files. After the study is completed, local data are stored with other completed research studies in a secured storage vault.

In compliance with the Health Insurance Portability and Accountability Act (**HIPAA**) and the Standards for Privacy of Individually Identifiable Health Information of the Department of Health and Human Services, LIFE accesses personal health information and **medical records** only after receiving signed **informed consent.** Participants' medical records are obtained, reviewed and abstracted. Such records are in a locked cabinet that is separate from other files cabinets and that uses a different key from that of all other files.

Biological samples. LIFE complies with the OHRP requirements and guidelines related to the research use of stored biological samples as stated in "Issues to consider in the research use of stored data or tissues" from the OPRR. (http://ohrp.osophs.dhhs.gov/humansubjects/guidance/reposit.htm).

Use of stored biological samples by other investigators. Biological samples may be used by investigators other than the investigators of the current study. The use is limited to non-commercial purposes.

Storage and disposal of data and biological material. Data, DNA and other biological components are stored for up to 40 years after study completion after which time all data and samples will be destroyed. All specimens have numerical samples IDs with no personal identifiers.

9. Feasibility Evaluation and Stopping Rules

9.1. Feasibility Evaluation of Definitive Trial

The data from the LIFE-P Study have demonstrated the feasibility of recruiting a geographically and ethnically diverse population of older community-dwelling adults at high risk for mobility disability into a physical activity intervention trial. The feasibility of the LIFE Study will depend on the ability to recruit, to maintain participant adherence, and to deliver a consistent intervention across sites, among other factors. No explicit feasibility criteria are established for The LIFE Study; however, the progress of The LIFE Study group and the study's potential of attaining its goals will be regularly evaluated by the Data and Safety Monitoring Board (DSMB). This committee reviews and provides feedback to the NIA on the overall performance of the study group, including its success with respect to goals for recruitment, retention, and data quality.

9.2. Stopping Based on Safety Concerns

At each meeting, the DSMB will review data on adverse events and other safety issues to make an overall recommendation to the NIH concerning the safety of continuing The LIFE Study. While adverse events will be monitored regularly, as is standard practice, formal statistical stopping boundaries will not be developed for these outcomes. Consistent with NIH policy, each Field Center Principal Investigator receives a report summarizing the DSMB review of the adverse event data. Field Center Principal Investigators are responsible for providing this report to the IRB.

9.3. Monitoring the Primary Outcome

LIFE also may be monitored for futility and efficacy by its DSMB. The DMAQC will work with the DSMB to develop statistical monitoring plans.

Futility may be monitored through the use of **unconditional** and **conditional** power. **Unconditional power** is computed based on assumptions about event rates and initial assumptions about intervention effects. Once the trial is underway, unconditional power may be recomputed based on more recent external data or on within-trial (pooled or control) event rates and recruitment and retention data. **Conditional power** is the probability of reaching a statistically significant intervention group difference in the primary endpoint at the planned end of the trial based on the current observed data (including the observed intervention effect) and assumptions about future data. It may be computed under a range of hypothesized future intervention effects.

10. Assessment Schedule and Outcomes Ascertainment

10.1. Summary of Baseline and Follow-Up Assessments

The schedule of clinic visits, procedures and assessments is summarized in Table 10.1.

Pre-randomization screening visits. The preliminary phone screen focuses on inclusion and exclusion criteria. Those who qualify are invited for the first two screening clinic visits (SV1 and SV2, Table 10.1.). At SV1, participants are asked to give the informed consent before any study procedures are performed. Participants are administered the SPPB,[114] the CHAMPS, and the 400 m walk to further assess study eligibility. A personal interview is administered, which focuses on medical history, inclusion and exclusion criteria, socio economic, health-related factors, physical activity and physical disability questionnaires. Medication use is assessed. Participants undergo measurement tests for blood pressure, pulse rate, anthropometric measures, cognitive testing, a focused physical exam, and a physician evaluation. The 400 m walk test is administered after the health professional or study physician has reviewed all medical assessments, including physical exam, medical history, medication use, and ECG. Participants who meet the initial entry requirements are then invited to complete the remainder of the baseline assessments at a second screening visit (SV2). During this visit the following assessments are administered: 3-MS Exam and cognitive battery, complete cognitive assessment (in a subset), quality of well being, health care utilization, phlebotomy, grip strength, Health Related Quality of Life (HRQL). After review of the Study Eligibility Review Checklist, those who qualify are randomized to the one of the two intervention programs.

To ensure blinding of the assessment staff to intervention assignment, the randomization is performed by staff members that are not involved in the assessments.

Clinic follow-up visits occur every six months as summarized in Table 10.1. Blood and urine will be collected at baseline, 6 months (only at Northwestern University, Tufts University, University of Pittsburgh, and Wake Forest University),12 months and 24 months. The **Cognitive Assessment Battery** will require about an hour and will be administered to all participants at baseline (3MSE, HVLT, Modified Rey-O copy and DSST at SV1 and computer-based battery at SV2), at 18 months (computer-based battery) and at 24 months and PIV visits (all tests from SV1 plus Category Fluency, Boston Naming, and Trails A and B). When the 3MSE is not administered, participants will be evaluated for cognitive impairment with the 6-item screener. When the participant makes more than 3 errors, a proxy informant should be interviewed to ascertain the non-cognitive study outcomes. In cases where the 6-item screener yields 3 errors or less but staff feels that the participant is seriously impaired cognitively, the LIFE staff member can decide, in consultation with the site PI, that a participant's proxy should be interviewed.

The **close-out** visit occurs for participants who did not receive a follow-up visit in the past three months. Every effort is made to conduct the study visits in the clinic. If participants are unable to come to the clinic according to the 8-week time window, the assessments are done in home or institution, as indicated. If participants are not available for in-person visits, personal or proxy telephone interviews are conducted.

Following the completion of the study intervention period, all participants will be asked to return for an additional follow-up assessment visit, **Post Intervention Visit.** Participants will be consented for this additional visit.

Safety monitoring. Safety is assessed at each clinic visit by in-person interviews and by means of telephone and proxy interviews.

Table 10.1 Assessments Schedule

Visit type	Scr	Scr	Rnd	Fu	Fu	Fu	Fu	Fu	Fu	Fu	Fu	Fu
Visit Code		SV1	SV2	F06	F12	F18	F24	F30	F36	F42	Cls	PIV
Clinic or Home Visit number		1	2	3	4	5	6	7	8	9	10	11
Telephone call	1											
Activity/assessment Month		-0.5	0	6	12	18	24	30	36	42		
**Form name**												
Verbal Consent	x											
Telephone screener	x											
SPPB and CHAMPS Consent		x										
SPPB Battery		x		x	x	x[~]	x	x[~]	x	x[~]	x	x
CHAMPS-18[x]		x		x	x		x		x		x	x
Informed Consent		x										
Post-intervention Informed Consent												x
Contact Information / update		x		x	x	X	x	x	x	X	x	x
Demographic, social, economic		x										
BP, Radial Pulse and Weight		x		x		x		x				x
Waist Circumference		x					x					
Physical exam, Body height		x										
Medication inventory		x			x							
Medical, hospital admission history		x										
Cancer follow-up form[+]		x[+]										
Cardiovascular medical history baseline update form[+]		x[+]										
ECG		x				x					x**	
Disability Questionnaire		x		x	x	x[a]	x	x[a]	x	x[a]	x	x
400 M Walk Test *		x*	*	x	x	x	x	x	x	X	x	x
Accelerometry[#]		x[#]	[#]	x	x		x					x
Process measures		x^	^		x			x				
Mobility Assessment Tool, short form MATsf			x			x		x				
3-MS Exam and cognitive battery		x					x[°]					x
EPrime cognitive assessment[&]			x			x[&]		x[&]				
Quality of well being (CEA)			x	x	x	x	x	x	x	X		x
Health care utilization (CEA)			x	x	x	x	x	x	x	X		x
Study Eligibility Checklist			x									
NEWS-A			x									
Ankle Brachial Index (ABI)			x					x			x**	
Claudication questionnaire			x					x			x**	
Sleep-wake disturbances			x	x		x		x				
Pulmonary questionnaires, ventilatory capacity			x	x		x		x				
Phlebotomy/Blood Processing/Urine			x[@]	x[@@]	x		x					
Grip strength			x		x							x
Health Related Quality of Life (HRQL)			x		x		x					
Proxy ADL Questionnaire[%]				x	x	x	x	x	x	x	x	x
Assistive Device Questionnaire[$]				x	x	x	x	x	x	x	x	x
Outcome Events				x	x	x	x	x	x	x	x	x
Other Health Related Events Questionnaire				x	x	x	x	x	x	x	X	
Six –Item Screener				x	x	x		x	x	x	x	

Scr=Screening visit; V=Visit; Rnd=Randomization; F=follow-up visit; Cls=Close out visit; CEA=Cost Effectiveness Analysis; PIV=Post intervention visit; [~]The SPPB test is administered when the 400 m walk test is not attempted. [x]CHAMPS-18 can be completed during group information/screening sessions prior to SV1; [+]The Cancer Follow-up Form and Cardiovascular Medical History Baseline Form can be collected during a subsequent follow-up visit during the first 24 months if not collected prior to randomization.[a] An abbreviated Disability Questionnaire will be administered at the 18, 30, and 42 month visits. *The 400 m walk test can be administered either at the end of SV1 or at SV2; ^Only 1 of the measures needs to be completed immediately after the 400 m walk: Efficacy for Walking; the others should be completed at SV2; [#]Accelerometry is performed in all participants at each site in conjunction with the 400 m walk; [˚]Participants who do not complete the cognitive battery at the 24 month visit or PIV will be administered the Telephone Interview of Cognitive Status (TICS). For those participants that do not complete the TICS or who score < 30 on the TICS, the Dementia Questionnaire will be administered to the participant's proxy. [&] At follow-up, the EPrime computerized testing will be administered at the 18 month visit; a small number of participants will complete this at their 30 month visit rather than their 18 month visit. [@]Includes lipid and metabolic panel, and CBC; [@@]The 6-month phlebotomy is performed only at the following sites: Northwestern University, Tufts University, University of Pittsburgh, and Wake Forest University. Urine samples are not collected at the 6-month visit. [%]The proxy ADL questionnaire is administered when a participant is not available to complete a follow-up assessment or is deemed to be cognitively impaired based on the Six-item screener. [$]The Assistive Device Questionnaire is administered at any visit at which the 400 m walk test is not attempted. **Measures to be obtained at closeout only if the measures were missed at the scheduled follow-up or if the scheduled follow-up visit occurred more than one year to the closeout visit.

10.2. Primary Outcome Measure: Major Mobility Disability

The primary outcome for the full-scale trial is time to the onset of major mobility disability. Mobility disability is determined by the objective 400m walk or by adjudicated evidence that the individual could not perform the 400 m walk. The objective component of major mobility disability is defined as the inability to complete a 400 m walk test within 15 minutes without sitting or the help of another person. Individuals who complete the walk in more than 15 minutes have an extremely slow pace (<0.45 m/sec), which would make their walking capacity of little utility in daily life.[91] Major mobility disability is assessed every six months by staff who are blinded to the intervention.

10.2.1. Time Frame For Follow-Up Assessments Of The Major Mobility Disability Outcome

The primary and secondary outcomes are assessed at baseline and at semiannual follow-up visits. Participants receive a modest payment for each assessment and are provided with transportation to the assessment clinic as needed. If the participant is unable or unwilling to come to the clinic, the assessment is done in the participant's home or institution. Every effort is made to personally interview and assess all participants. Telephone interviews are conducted in the event that personal assessments are not possible. Some flexibility is needed to account for occasions when participants are not readily available to complete the outcomes assessment, e.g., during acute care hospital admissions. To minimize the possibility of missing an assessment, the following protocol is followed:

1. At the time of enrollment, two persons are identified (names, addresses, phone numbers) who do not live with the participant and who would likely know the whereabouts of the participant if he/she could not be contacted for a follow-up assessment;
2. For each follow-up assessment, LIFE allows a 4-week window for completion on each side of the "anniversary" date (for a total of 8 weeks); the time between assessments is no shorter than 5 months and no longer than 7 months;
3. If the participant is acutely ill, is in the hospital, has a temporary condition that interferes with walking capacity (for example, ankle sprain or foot surgery), or is otherwise unavailable, LIFE attempts to complete the assessment at another time within the 8-week window; if possible, LIFE waits at least one week after an acute illness or hospital discharge to complete the assessment;

Since participants who are acutely ill may subsequently die, LIFE attempts to determine their self-reported major mobility disability during the initial contact, to minimize potential losses to follow-up; this information is used if the participant subsequently dies or refuses to complete the follow-up assessment. Proxy respondents are also used during follow-up to assess the mobility status.

10.3. Secondary and Tertiary Outcome Measures

The secondary outcomes include cognitive function; serious fall injuries; persistent mobility disability; the combined outcome of major mobility disability or death; proportion of 400 m walk failures over time; disability in ADLs; and cost-effectiveness.

10.3.1. Cognitive Function

Cognitive function is assessed at baseline and at the 24-month follow-up. The DSST and HVLT are the two battery components that serve as the secondary cognitive outcomes.

10.3.2. Serious Fall Injuries

Serious fall injuries include only those falls that result in a clinical, non vertebral fracture and/or lead to hospitalization for one of the reasons described below. Falls that meet these criteria are associated with the greatest morbidity and costs.[76] Criteria for serious fall injuries do not include other adverse consequences of falls, e.g., restricted activity, fear of falling, non-fracture injuries that do not lead to hospitalization, etc.

1. Non-Fracture head injury with loss of consciousness, subdural or other intracranial hematoma by MRI or CT, facial trauma requiring sutures with report, traumatic vision loss with report of visual function, or other similar head injury sequela.

2. Consequences of long lie such as rhabdomyolysis with increases in muscle enzymes dehydration with use of parental fluid replacement, decreased blood pressure, increase BUN and/or sodium level, or hypothermia (rectal temp $< 36^{o}$ C)
3. Other injury attributed to a fall such as burns with report of burn severity and extent, severe sprains with description of swelling, pain and change in motion, visceral injury with radiologic confirmation of blood collection or change in organ features, internal bleeding with report of hematoma or need for surgery, or acute peripheral nerve damage with neurological description of acute deficit.

10.3.3. Persistent Mobility Disability

Persistent mobility disability is defined as having major mobility disability at two consecutive clinic visits.

10.3.4. Self-Reported Disability

As described in Chapter 6, the primary measures of self-reported function are the MAT-sf and the 19-item Pepper Assessment Tool for Disability (PAT-D).

In addition, for the basic ADLs the participant is also asked whether the participant receives help from another person to complete the task. This allows calculation of a Katz ADL score. The proxy ADL questionnaire is administered when a participant is not available to complete a follow-up assessment or is deemed to be cognitively impaired based on the Six-item screener.

10.3.5. Cost Effectiveness

Quality-adjusted life years (QALYs) are used for cost-effectiveness analysis.[185-187] QALYs integrate mortality and morbidity to express health status in terms of equivalents of well-years of life.

Although there has been considerable interest in measuring cost effectiveness of treatments in old age, the validity of most general preference weighted measures has not been well evaluated in this field. One exception is the **Quality of Well-being Scale (QWB)**, which has been used in several trials with seniors.[149, 151, 153, 185-193] Studies using the QWB suggest that physical activity interventions may produce benefits for older adults at a cost comparable to many widely advocated programs.[194] In addition to the ease with which QALYs can be calculated from the QWB-SA, the measure allows for specific areas of clinical improvement to be identified among its 58 acute and chronic symptoms.

10.3.6. Tertiary outcomes include (a) the combined outcome of MCI or dementia, (b) a composite measure of the cognitive assessment battery, (c) the SPPB score and 400 m walk speed assessed in ethnicity/race, gender and baseline performance subgroups; (d) time by intervention interactions on the SPPB score and 400 m walk speed endpoints; (e) sleep-wake disturbances, (f) dyspnea, (g) ventilatory capacity, (h) pulmonary events and (i) cardiovascular events.

(a) Mild Cognitive Impairment/Probable Dementia (MCI/PD). We will contrast the rates of all-cause MCI/PD at 2 years. While assessment of the impact of PA on the incidence of dementia and mild cognitive impairment is of great interest, LIFE may not have sufficient power for a definitive result. This outcome will therefore be explored as a tertiary aim. Cases will be identified using a **2-staged** approach similar to what has been found to be efficient in other trials of cognitive studies (Appendix A).[195-199]
Stage 1 is the LIFE cognitive screening battery (See 6.2.11) followed by a more detailed neuropsychological assessment for incident MCI or dementia. In **stage 2**, MCI/PD status will be adjudicated by an **expert panel** (masked to intervention assignment) based on all cognitive assessments (administered by centrally trained and certified staff), informant based report of functional impairment, and review of medical records.

(b) A composite measure derived from the cognitive assessment battery (DSST, HVLT, Flanker, N-back and Task Switching), excluding the 3MSE, will be standardized and averaged to form a single composite score for additional analysis as a tertiary outcome.

(c) Short Physical Performance Battery (SPPB). The SPPB, originally developed for the Established Populations for the Epidemiologic Study of the Elderly (EPESE) is a brief performance battery based on timed short distance walk, repeated chair stands and balance test (as described by Guralnik et al.)[90, 112-116]

(d) **Sleep-Wake Disturbances** will be evaluated with four validated instruments: Epworth Sleepiness Scale (ESS),[157] Insomnia Severity Index (ISI),[158] Pittsburgh Sleep Quality Index (PSQI),[159] and Berlin Questionnaire (BQ).[160]

(e) **Dyspnea**.
Dyspnea will be assessed with a modified version of the ATS-DLD-78-A questionnaire [ATS-DLD-78-A Adult Dyspnea Questionnaire. at http://www.cdc.gov/niosh/respire.html] and the Borg index for Dyspnea.[161]

(f) **Ventilatory Capacity**
Ventilatory capacity will be assessed by spirometry and maximal inspiratory pressure

(MIP). Spirometry will be recorded by the EasyOne™ PLUS spirometer. MIP will be recorded by a pressure gauge fitted with a disposable cardboard mouthpiece.

(g) Pulmonary outcomes
Pulmonary outcomes to be assessed include hospital admission for exacerbation of airway disease (asthma, bronchitis, emphysema, &/or COPD) or pneumonia. Initial ascertainment of pulmonary hospitalizations will be made at the every 6 month follow-up interviews. Medical records will be obtained and reviewed blinded to group assignment. Details of the review and adjudication are provided below.

(h) Cardiovascular outcomes
Cardiovascular events to be assessed as tertiary outcomes include the combined outcome of:

- Myocardial infarction (MI),
- Angina requiring hospitalization,
- Any stroke (ischemic or hemorrhagic),
- Transitory ischemic attack (TIA) requiring hospitalization,
- Hospitalization for carotid artery disease,
- Hospitalization for congestive heart failure (CHF),
- Hospitalization for peripheral artery disease (PAD) or outpatient revascularization for PAD,
- Ruptured abdominal aortic aneurism (AAA), and
- CVD death.

Initial ascertainment of cardiovascular hospitalizations and procedures will be made at the every 6 month follow-up interviews. Medical records will be obtained and reviewed blinded to group assignment. Details of the review and adjudication are provided below.

10.4. Outcome Adjudication Procedures
10.4.1. Adjudication of the Major Mobility Disability Outcome

Final determination regarding when study participants reach the major mobility disability outcome is made by the Outcomes Committee, using an adjudication process. Final assignment of endpoint requires unanimous agreement by the committee which reviews cases at least every 6 months. In most instances, the outcome of major mobility disability is readily apparent from the results of the 400 m walk performance test. All subjects who attempt but do not complete 400 m in 15 minutes or less are categorized (on the day of the assessment) as **mobility disabled**. Any individual requiring an alternative assessment has all available records summarized and reviewed by the committee, for determination of disability status. These alternative contacts may include a home visit, telephone interview with participant or proxy, or review of hospital records.

The process of review of all alternative contacts is sensitive to cases of possible disability and minimizes loss-to-follow-up by making use of all available information. The criteria for adjudication of these potential cases as mobility disabled are designed to maximize specificity and minimize bias. There may be some cases where only self-report or proxy-report may be available. LIFE has designed an interview that is quite specific, as compared to performance testing. No self- or proxy-report instrument is perfectly sensitive and specific; hence the primary method of outcomes assessment must be based on objective performance. Those with inadequate information for a definite diagnosis of major mobility disability are considered to be non-disabled for mobility. Some may become mobility disabled by the subsequent visit. Every effort is made to obtain follow-up contact within the required time window using the same methods in both intervention groups. All

outcomes assessments are performed by researchers who are blinded to group assignment. The committee is also blinded to intervention assignment. This approach is similar to methods used for adjudication of cardiovascular disease, cancer, dementia, fracture and other health outcomes in RCTs.

Hierarchical Adjudication of Major Mobility Disability

Definite: ANY of the Following:

(1) Primary
Unable to complete 400-meter walk in 15 minutes without sitting or the help of another person

(2) Alternative (in home or clinic)
Unable to walk 4 meters without assistance of another person or use of a walker OR (or comparable device) Unable to complete 4-meter walk test in 10 seconds or less, i.e. gait speed less than 0.4 meter/sec

(3) Alternative (telephone or in home)
(a) Self report of inability to walk across a room (12 ft) without the assistance of another person

Operationally, this criterion is met based on an affirmative response to one or more of the following 2 questions:

(i) respondent answers "unable to do" when asked, "During the past month, how much difficulty have you had walking across a small room because of your health?"

(ii) respondent answers "Yes" to "Do you usually receive help from another person when you walk across a small room";

OR

(b) Proxy report of inability to walk across a room (12 ft) without the assistance of another person

Operationally, this criterion is met based on an affirmative response to the following question:

(i) proxy answers "Yes" to "Does (participant) usually receive help from another person when he/she walks across a small room";

(4) Alternative (medical record)
Documentation of inability to walk across a room (12 ft) without the assistance of another person or use of a walker (or comparable device); example of descriptors include: bed-bound or wheelchair-bound, obtunded or moribund, etc.

10.4.2. Death
The fact and date of death are confirmed by death certificate. Cause is determined from hospital records, death certificate and informant interview information and coded into major categories of death.

10.4.3 Serious Fall Injuries
Clinical fractures[200] are defined as fractures involving any skeletal site (except

vertebral) that occur after randomization, are diagnosed because of fracture-related symptoms, are reported to the investigators, and are documented by a definite radiologic diagnosis (radiographs, bone scan, etc.). These fractures are ascertained using a protocol[200] that was originally developed by the FIT (Fracture Intervention Trial) investigators and is now being used in the Osteoporotic Fractures in Men Study.

Data are collected on all hospital admissions. These data are reviewed by an expert physician, who is masked to the intervention group, and who identifies hospital admissions that are primarily attributable to a fall injury, including: fractures, head injuries resulting in loss of consciousness, joint dislocations and other serious joint injuries, severe lacerations, serious internal injuries (e.g., retroperitoneal hematoma) and the major sequelae of aging (rhabdomyolysis, dehydration, and hypothermia).

10.4.4 Pulmonary and cardiovascular outcome adjudication

All hospital records will be reviewed to determine whether there was a pulmonary or cardiovascular outcome for the following conditions:

a) Pulmonary outcomes to be assessed include hospital admission for exacerbation of airway disease (asthma, bronchitis, emphysema, &/or COPD) or pneumonia. Initial ascertainment of pulmonary hospitalizations will be made at the every 6 month follow-up interviews. Medical records will be obtained and reviewed blinded to group assignment. Details of the review and adjudication are provided below.

b) Cardiovascular outcomes to be assessed as tertiary outcomes include the combined outcome of:

- Myocardial infarction (MI),
- Angina requiring hospitalization,
- Any stroke (ischemic or hemorrhagic),
- Transitory ischemic attack (TIA) requiring hospitalization,
- Hospitalization for carotid artery disease,
- Hospitalization for congestive heart failure (CHF),
- Hospitalization for peripheral artery disease (PAD) or outpatient revascularization for PAD,
- Ruptured abdominal aortic aneurism (AAA), and
- CVD death.

Initial ascertainment of hospitalizations will be made at the every 6 month follow-up interviews. Medical records will be obtained and abstracted for codes and diagnoses compatible with one f these events and will be blinded to group assignment. This summary data along with a de-identified copy of the records will be forwarded to the DMAQC for review by a central adjudication committee. The committee will be composed of clinicians with expertise in the diagnosis of these conditions (cardiologists, pulmonologists and geriatricians) who will assign presence or absence of each diagnosis using standardized criteria.

If death is reported to be in the hospital or subsequent to a hospitalization (in nursing home, hospice or home), hospital records, autopsy reports if available, and death certificate will be requested and reviewed. Abstraction forms and de-identified records will be forwarded to the DMAQC for review and adjudication by the central adjudication committee based on standard criteria to determine whether one of the cardiovascular outcomes of interest is the underlying cause of death. For out-of-hospital deaths, an interview will be conducted with the previously designated informant to determine the

circumstance of death.

10.5. Ancillary Studies

Proposals of ancillary studies are subject to review by the Emerging Science Committee.

10.6. Tracking Health Care Utilization and Vital Status, and HIPAA Compliance

Health care utilization is assessed at baseline and every 6 months using a self-administered questionnaire developed at the University of California San Diego. The measure consists of 12 questions that ask about the frequency of various types of health care utilization over the previous 6 months. The questions ask about utilization of hospital days, emergency care, urgent care, primary care, telephone calls, prescriptions, and medical equipment. Health care costs are calculated by multiplying the frequency of each service by the prevailing community charge. The measure has been validated in a clinical trial of patients with chronic obstructive pulmonary disease.[156]

In compliance with the **HIPAA** and the DHHS' Standards for Privacy of Individually Identifiable Health Information, LIFE accesses personal health information and **medical records** only after obtaining **informed consent.** Social security number, Medicare number, date of birth, and health insurance information is collected. A proxy respondent and two contact persons are also identified.

Abstraction of hospital records. Participants are asked to notify study personnel about any hospitalization or serious illness. At each clinic visit or telephone contact, participants are questioned about interim hospitalizations. Since any hospitalization may potentially result in changes in activity level or performance, LIFE abstracts minimal information on all hospitalizations (discharge diagnoses, procedures, and length of stay). Hospitalizations are reviewed to assess study outcomes. For fractures, type of fracture and treatment are verified by review of the medical record. Reports of **death** through regular surveillance or via databases searches are tracked by collecting death certificates and relevant medical records, including autopsy reports if available. For all medical record reviews, standard forms are completed by trained abstractors.

11. Data Management and Quality Control

11.1. Data Management

11.1.1. Field Centers-Screening, Randomization, and Follow-Up Visits

An internet-based, web browser application is used to manage screening, randomization and follow-up visits in this project. Field Centers access the study web site and initiate the interactive randomization page. Entry into this area is password protected and encrypted. Once security requirements have been satisfied, a series of questions establish identifying and eligibility information, and a participant identification number is issued. When the randomization session is complete, an e-mail process is initiated and a record of the transaction is sent to the clinic coordinator and the project manager at the DMAQC indicating that the participant has been properly appended to the database.

The 'Participant Tracking System' (PTS) is a fully integrated tracking and notification system that advises clinic staff about participant follow-up windows and projects clinic and laboratory workload. Participant tracking begins at screening and randomization and continues automatically throughout the project by integrating participant follow-up data with predetermined follow-up "windows." Reports about protocol deviations are automatically generated and transmitted to the clinic via e-mail attachments. These data are available in the study web site.

A Participant Label tool will also be included in the system, to assist in the creation and printing of the various labels needed during data collection. This system will support multiple label formats and label paper stock.

11.1.2. Data Entry, Verification and Quality Control

Field Center data coordinators review each set of completed forms for accuracy and completeness. During data entry, key variables are checked for accuracy with the assigned range checks. A review is required for any data entered outside of preset ranges. Override capabilities exist; however these are flagged for review upon receipt by the DMAQC. Through communication with the clinic coordinators, the DMAQC project manager reconciles any responses that continue to be questionable within 30 days. A random verification pass is performed to detect error patterns and logic flaws

11.1.3. Intentionally Blank

11.1.4. Database Closure

Upon study completion, after all clinic and laboratory data have been collected and filtered through the appropriate quality control procedures, the database is certified. The database is taken off-line and archived. The final datasets are certified and issued version numbers to synchronize analytic efforts, after which they are distributed in accordance with Steering Committee and Institute policy.

11.2. Management of Administrative Data

A Web-based administrative tracking and monitoring system facilitates the flow of information and increases the level of communication within LIFE. Its Web site includes the Study Directory, meeting times and locations, minutes, data reports, IRB status of projects, and other procedural, technical or administrative documents.

11.3. Quality Control (QC)

QC is a shared responsibility of all investigators. The DMAQC takes a vigorous lead in assuring the quality of study databases. The quality and eventual acceptance of all studies depend on issues such as: maintaining randomization integrity, accurately assessing participant eligibility, recording dropouts and adherence, measuring outcome

variables without bias, preventing premature release of results, monitoring and assessing protocol adherence, and avoiding biases in the analysis of the results. QC procedures are devised to monitor screening, data collection, follow-up, clinical measurements, collection of forms, data entry procedures, implementation of interventions and overall scientific and leadership operations.

12. Statistical Considerations

12.1. Introduction and Aims

The **primary aim** of the LIFE randomized controlled clinical trial is to assess the long-term relative effect of randomization to a physical activity intervention on the **primary outcome** of time until major mobility disability defined as inability to walk 400 m in sedentary persons aged 70-90 years.

The **secondary aims** of the LIFE trial are to assess the relative effect of randomization to the intervention on the following **secondary outcomes**:

- Cognitive function based on the Digit Symbol Test (DSST) and the Hopkins Verbal Learning Test (HVLT)
- Serious fall injuries;
- Persistent mobility disability and the combined outcome of major mobility disability or death;
- Proportion of 400 m walk failures over time;
- Disability in activities of daily living; and
- Cost-effectiveness.

Tertiary aims of the LIFE trial are **to explore** the effects of random assignment to the intervention on the following **tertiary outcomes**:

- Mild cognitive impairment / dementia;
- A composite measure of the cognitive assessment battery; and
- The Short Physical Performance Battery (SPPB) score and the 400 m walk speed within ethnicity/race, gender and baseline physical performance subgroups;
- Sleep-wake disturbances;
- Dyspnea and ventilatory capacity;
- Hospital admission or death for exacerbation of airway disease (asthma, bronchitis, emphysema, &/or COPD) or pneumonia;
- Combined cardiovascular events including:
    - Myocardial infarction (MI),
    - Angina requiring hospitalization,
    - Any stroke (ischemic or hemorrhagic),
    - Transitory ischemic attack (TIA) requiring hospitalization,
    - Hospitalization for carotid artery disease,
    - Hospitalization for congestive heart failure (CHF),
    - Hospitalization for peripheral artery disease (PAD) or outpatient revascularization for PAD,
    - Ruptured abdominal aortic aneurism (AAA), and
    - CVD death;
- In addition, as additional **tertiary aim**, we will explore the effect of the length of the intervention on the SPPB score and 400 m walk speed endpoints (i.e. time since randomization by intervention effect.

12.2. Analysis Plans

The primary aim is to assess the long-term relative effect of randomization to a physical activity intervention on time until major mobility disability (defined as inability to walk 400 meters (through objective or adjudicated evidence).

The primary study hypothesis of LIFE will be tested based on a two-tailed significance level of 0.05. In this analysis, the "intention to treat" approach will be used in

which participants are grouped according to randomization assignment.

The main comparisons of intervention groups with respect to the distribution of time until the first post-randomization occurrence of a primary outcome will be based on survival analyses. To compare intervention arms, we will use a likelihood ratio test from a Cox regression model, stratified by Field Center and gender. Failure time is measured from the time of randomization.

Secondary aim #1 will be to assess the relative effect of randomization to the intervention on cognitive function as measured by the DSST and HVLT instruments.

Cognitive data will be collected at the baseline and 2-year visits. For the primary DSST and HVLT analysis, the data will be transformed to better approximate the conditional normality assumption if necessary. Analysis of the intervention effect will be carried out using analysis of covariance with variables in the model representing field center, gender, the baseline outcome and the intervention effect. The effect of the intervention on DSST and HVLT will be based on a two-tailed significance level of 0.05 and will use the “intent to treat” approach.

Secondary aim #2 will be to assess the relative effect of randomization to the intervention on serious fall injuries.

The main comparisons of intervention groups with respect to the distribution of time until the first post-randomization occurrence of a serious fall injury will be based on survival analyses. To compare intervention arms, we will use a likelihood ratio test from a Cox regression model stratified by gender. Due to the expected small number of serious fall injuries, we have chosen not to stratify this outcome by field center. Failure time is measured from the time of randomization.

Secondary aim #3 will be to assess the relative effect of randomization to the intervention on persistent mobility disability.

Of primary interest is a comparison of the probability of being classified as having major mobility disability at two consecutive assessments. This outcome will be analyzed using transitional models for categorical endpoints. A first-order Markov model will be used to compare between intervention groups the probability of being classified in the major mobility disability category at a subsequent visit, given a participant’s previous classification state. Validity of the first-order versus higher-order Markov assumptions will be explored within these models.

Secondary aim #4 will be to assess the relative effect of randomization to the intervention on the combined outcome of major mobility disability or death.

The main comparisons of intervention groups with respect to the distribution of time until the first post-randomization occurrence of the combined outcome of major mobility disability or death will be based on survival analyses. To compare intervention arms, we will use a likelihood ratio test from a Cox regression model, stratified by field center and gender. Failure time is measured from the time of randomization.

Secondary aim #5 will be to assess the relative effect of randomization to the intervention on the proportion of 400 m walk failures over time.

This analysis will use the repeated 6-month indicators of 400 m walk status (rather than the time until the initial failure) and compare the average proportion of participants in each intervention group that fail the 400 m walk across all time points using generalized estimating equations (GEEs) that account for the dependency between repeated measures. Odds ratios for the association between 400 m walk status and intervention will be estimated after adjusting for field center, gender, a follow-up time effect, and an intervention effect. This approach is most consistent with 400 m walk serving as a marker of functional status and the trial examining whether lifestyle interventions may alter the distribution of this construct.

Secondary aim #6 will be to assess the relative effect of randomization to the

intervention on the disability in activities of daily living.

Level of ADL disability will be repeatedly measured as a continuous, score variable. A comparison of average post-randomization levels of ADL disability between intervention groups will be performed using mixed-effects analysis of covariance techniques appropriate for repeatedly measured outcomes. These models will contain variables representing field center, gender, a follow-up time effect, the baseline level of ADL disability and an intervention effect. Initially, we will explore the appropriateness of a model containing random intercepts and slopes (for follow-up time). The appropriateness of a model containing a fixed effect for the intervention by follow-up time interaction will be evaluated.

Secondary aim #7 will be to assess the cost-effectiveness of the intervention.

Cost-effectiveness analyses will be conducted following the guidelines of the Panel of Cost-Effectiveness in Health and Medicine. The ratio of direct costs of the physical activity intervention to the amount of quality-adjusted life years (QALYs) produced is calculated. Health care costs will be estimated and differences between the physical activity and lifestyle intervention groups will be calculated to examine whether any cost-offset may occur. LIFE takes a societal perspective. The trial uses the health education intervention as the comparator for all cost-effectiveness analyses. Results will be described as the incremental cost-effectiveness over the comparator. Sensitivity analyses will be conducted to examine whether the cost-effectiveness results change as a function of any estimates or assumptions made in the process. Decision modeling will be used to estimate long-term cost-effectiveness beyond the 1-year time horizon for which data collection is planned. Future health care costs will be discounted at a rate of 3% for any calculations or projections beyond the first year of follow-up.

Tertiary aim #1 will be to explore the effects of the interventions on MCI/D.

Logistic regression will be used to assess whether the proportion of participants who convert to the composite outcome of MCI/D from baseline varies by intervention assignment. Baseline level of global cognitive function (3MSE score) will be used as a covariate in these analyses.

Tertiary aim #2 will be to explore the effects of the intervention on composite measure of the cognitive assessment battery. We will also construct a composite measure to include all components of the Cognitive Assessment Battery (but not the 3MSE, which is used as to screen participants for MCI/D classification). For this composite measure of cognitive performance we will z-transform each score by dividing its difference from the baseline mean by the baseline standard deviation, and then will average the z-transformed measures. This approach, rather than adopting weighted averages, provides slightly greater emphasis on executive function.] The relative effect of the PA intervention on this composite will be assessed with analyses of covariance applied to the measures collected at Year 2, with the baseline composite included as a covariate. Differences between intervention arms for each individual test that contributes to the composite measure will be described. A similar approach will be used to assess the impact of the intervention on executive functions using a composite score for the three executive function tests (Flanker, N-back and Task Switching)

Tertiary aim #3 will be to assess the effects of the interventions on the SPPB score and 400 m walk speed within ethnicity/race, gender and baseline physical performance subgroups. Repeated measures analysis of covariance techniques for continuous outcomes (such as described for ADLs) will be used to explore these effects (mixed effects models). Estimates of the intervention effect will be obtained within these subgroups and formal tests of interactions between intervention and subgroup variables will be performed. Forest plots will be used to graphically display the results of these subgroup analyses.

Tertiary Aims #4 will be assessed using a Cox proportional hazards model, stratified by Field Center and gender. Failure time is measured from the time of randomization to the first sleep-wake disturbance.

Tertiary Aim #5 will be assessed using a logistic regression model with adjustment for Field Center and gender for the outcome of dyspnea. Ventilatory capacity will be examined using two types of mixed model analysis of variance models. First, we will examine for different post-baseline mean levels of FVC and FEV1 using a model adjusting for the baseline level, time (categorical), Field Center, and gender. We will account for within-person correlation using a subject random effect. Second, we will examine for a difference in the rates of decline using a random intercept/random slope mixed model.

Tertiary Aims #6 and 7 will be assessed using Cox proportional hazards models, stratified by Field Center and gender. Failure time is measured from the time of randomization to the occurrence of a pulmonary disorder (Aim 6) or combined cardiovascular event (Aim 7).

Tertiary aim #8 will be to explore time by intervention interactions on the SPPB score and 400 m walk speed endpoints. Separate estimates of the intervention effect will be obtained for participants with different planned lengths of intervention. This will be achieved by introducing variables representing periods of randomization (i.e., groupings of consecutive months) into the repeated measures models for SPPB score and 400 m walking speed and exploring the interaction between these variables and the intervention effect. There may be some confounding between other factors and the month of randomization (e.g., staff familiarity with the intervention); however, this analysis could be important in determining how length of intervention may relate to efficacy. We also will use interaction terms like these to estimate intervention effects for 1-year epochs across follow-up to examine their consistency over time.

Tertiary aim #9 will be to explore whether the relative effect of the PA intervention on measures of cognitive function varies according to baseline level of global cognitive function. Separate estimates of the intervention effect on measures of cognitive function will be obtained for participants grouped by baseline 3MS (<90 versus $\geq$ 90). Tests of interaction will be used to compare any differences between these groups.

13. Trial Organization

Several centers, cores and committees support key components of the study and ensure its successful conduct and completion (Figure 13.1.).

Figure d.13.1. Study organization scheme

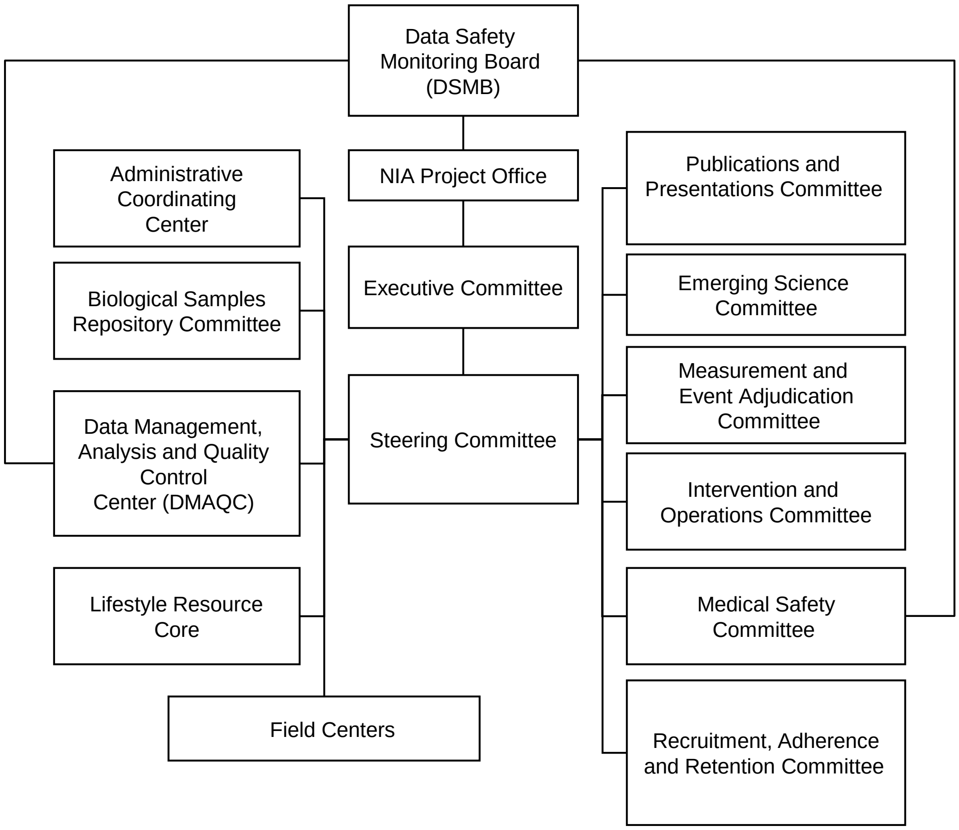

The **Steering Committee,** which is charged with the overall governance of study conduct, consists of selected investigators of the Field Centers and other support Centers, and the NIA Project Officers. The Steering Committee approves the final protocols and manuals of operations, supervises the overall execution of the trial, generates and approves study policies, considers modifications of the protocol and study operations, plans and drafts study-related publications, and plans the protocol for the full-scale study. The Steering Committee appoints and charges the subcommittees described below. All major scientific decisions are determined by majority vote of the Steering Committee.

The **Executive Committee** includes the Co-chairs of the Steering Committee, the NIA project officer and two rotating members selected among Field Center PIs and the DMAQC PI. The Executive Committee functions as the main liaison between the study investigators and the NIA, is responsible for negotiating budgets, the fiscal management of the trial, allocating resources based on pre-set budgets and on performance of individual Field and Support Centers, and evaluating and reporting on progress, timeline benchmarks and deliverables.

The **Administrative Coordinating Center** performs the following tasks:

- Development and monitoring of subcontracts with all sites, matching timelines and deliverables
- Coordination of meetings and conference calls for Committees and Sub-Committees:
- Development of the Manual of Operations (MOP), protocol and intervention materials
- Update the MOP, protocol and intervention materials
- Development of the questionnaires and forms jointly with the DMAQC Center and with relevant sub-committees of the Steering Committee (see below)
- Development of study recruitment materials, jointly with a Centralized Media Group
- Development of systems for communication among Steering Committee and sub-committee members
- Mailing of materials to the Field Centers
- Tracking of equipment and supplies
- Coordination of training and certification of Field Center staff.

The Data Management, Analysis and Quality Control Center (DMAQC) performs the following tasks:

- Development of sample size and statistical analysis plans, including stopping guidelines
- Development of the centralized web-based data management system
- Development and maintenance of the study wide web-based tracking and monitoring system
- Development of the decentralized participant tracking system
- Development of the randomization protocol and procedures
- Development of systems for obtaining data from the ECG reading center
- Generating data quality reports for study sites, Steering Committee and DSMB meetings
- Participating in presentation of blinded and unblinded data to the DSMB
- Monitoring of adverse events
- Monitoring of all QC aspects of data collection, including measurement and intervention reliability
- Reviewing of proposed ancillary study protocols
- Participation in writing teams for manuscripts

- Generating tables of results and performing analyses for manuscripts
- Establishing procedures for ensuring safety and confidentiality of records to meet HIPAA guidelines
- Establishing procedures for archiving and backup of data
- Participation in meetings and calls for all committees

The **Lifestyle Resource Core** serves as the primary group responsible for monitoring the fidelity and quality control of the intervention, training and certifying all project intervention staff involved in the intervention protocols and in operation of the computerized tracking system, and assisting interventionists with problem solving and related adherence strategies throughout the course of the intervention. The team reviews tracking system reports, operates e-mail and phone-based contact systems for assistance with the intervention protocol and provides advice on dealing with problems that arise in delivering the intervention.

The **Biological Samples Committee** coordinates and standardizes the collection, processing and short-term storage of blood samples across all Field Centers; and devises training and quality control procedures for the biological samples processing and storage at the Field Centers.

The **Field Centers** recruit study participants, administer the physical activity intervention and the health education control, ensure retention and adherence of study participants, perform all study related assessments (including complete tracking of outcomes during follow-up), and enter the data into the web-based data entry system. The Field Centers work with all other centers, cores and committees to ensure the accurate implementation of the study protocol and the successful conduct and completion of the trial. The Field Center investigators participate in the study committees and manuscript.

The **Publications and Presentations Committee** (P&P) (a) encourages production of high quality publications and presentations in a timely fashion, (b) encourages broad participation by the study investigators in publications and presentations, and (c) assures accurate maintenance of a database on study publications.

The **Emerging Science Committee** monitors the literature, scientific meetings and input received from colleagues on the cutting-edge science related to topics that are relevant to the project, and advises the Steering Committee on emerging scientific issues that may affect the conduct and future directions of the study. The Committee also reviews plans for ancillary study proposals, including those that involve utilization of biological samples.

The **Measurement and Event Adjudication Committee** refines the assessment protocols, and works closely with the DMAQC Center and the Field Centers to ensure the quality control procedures. The Committee refines and implements strategies for the outcomes tracking and adjudication. The **Cognition Sub-Committee** serves as the primary group responsible for the design and implementation of the cognitive function assessment aspects of the trial. This committee will also oversee training and quality assurance of the cognitive assessment aspects of the trial as well as the procedure for adjudicating the exploratory outcome of MCI/Dementia incidence in the LIFE Study. Scientific leadership for manuscripts that involve reporting of the impact of the LIFE interventions on cognitive function will also originate from this committee

The **Intervention and Operations Committee** finalizes and refines the intervention protocols, and works closely with the DMAQC Center and the Field Centers to ensure the QC procedures and training for the intervention. The Committee develops the Intervention Manual, refines and implements strategies to monitor compliance, and together with the Recruitment, Adherence and Retention Committee, refines and implements strategies to enhance adherence to the intervention.

The **Medical Safety Committee** reviews masked study data related to the overall safety of study participation, develops safety reports for the Data and Safety Monitoring Board, addresses IRB issues (related to participant safety) that may arise, reviews clinical practice-related issues and oversees the clinical safety of all study participants.

The **Recruitment, Adherence and Retention Committee** refines and optimizes protocols and strategies for recruitment, adherence and retention of study participants. The Committee oversees recruitment progress at all sites, intervenes in cases of under-recruitment, and reports recruitment progress to the Steering Committee. The Committee oversees retention efforts and investigates and intervenes when a site is having retention problems.

A **Data Safety Monitoring Board (DSMB)** monitors all aspects of the study, including those that require access to any blinded data.

14. Study Timeline

The overall duration of the funding period for this study is six years (Table 14.1). The first six months are dedicated to setting up the study, including subcontracting with the field centers, finalizing the manual of operations, standardization of the procedures, development of the web-based data entry and tracking system, staff recruitment, and training of the field centers.

Table 14.1. Trial timeline

**Funding Year**	**Year 1**				**Year 2**				**Year 3**				**Year 4**				**Year 5**				**Year 6**			
**Funding year quarter**	**1**	**2**	**3**	**4**	**1**	**2**	**3**	**4**	**1**	**2**	**3**	**4**	**1**	**2**	**3**	**4**	**1**	**2**	**3**	**4**	**1**	**2**	**3**	**4**
**Calendar Year**	**09**	**2010**				**2011**				**2012**				**2013**				**2014**				**2015**		
**Calendar year quarter**	**4**	**1**	**2**	**3**	**4**	**1**	**2**	**3**	**4**	**1**	**2**	**3**	**4**	**1**	**2**	**3**	**4**	**1**	**2**	**3**	**4**	**1**	**2**	**3**
**Activity**																								
Centers subcontracts	X																							
Manual of operations finalized	X																							
Procedures standardization		X																						
Data entry and tracking system		X																						
Field centers training		X																						
Recruitment material		X																						
Field Centers staff recruitment		X																						
Participants recruitment			X	X	X	X	X	X	X															
Intervention			X	X	X	X	X	X	X	X	X	X	X	X	X	X	X							
Participant follow-up			X	X	X	X	X	X	X	X	X	X	X	X	X	X	X			X	X			
Close-out visits																	X							
Data analyses			X	X	X	X	X	X	X	X	X	X	X	X	X	X	X	X	X	X	X	X	X	X
Publications	X	X	X	X	X	X	X	X	X	X	X	X	X	X	X	X	X	X	X	X	X	X	X	X

Participant recruitment begins in the third quarter of the first funding year and continues for 21 additional months. Participants are followed for an average of 2.7 years (range 1.9-3.5 years). Close out visits occur in the first quarter of year 5 of funding. Data analyses and publications are performed throughout the study after recruitment starts.

15. Participating Sites

15.1 Clinical Sites

Northwestern University
Chicago, Illinois

Pennington Biomedical Research Center
Baton Rouge, Louisiana

Stanford University
Stanford, California

Tufts University
Boston, Massachusetts

University of Florida
Gainesville, Florida

University of Pittsburgh
Pittsburgh, Pennsylvania

Wake Forest University Health Sciences
Winston-Salem, North Carolina

Yale University
New Haven, Connecticut

15.2 Coordinating Center

University of Florida
Gainesville, Florida

15.3 Federal Sponsors

National Institutes of Health, National Institute on Aging

15.4 Central Resource Centers

Data Management, Analysis and Quality Control Center (DMAQC)
Wake Forest University Health Sciences
Winston-Salem, North Carolina

Cost Effectiveness Analysis Center
University of California, San Diego
San Diego, California

Electrocardiogram Reading Center
University of Florida
Gainesville, Florida

Pulmonary Reading Center
Yale University
New Haven, Connecticut

16. Bibliography

Reference List

1 Manton KG, Vaupel JW. Survival after the age of 80 in the United States, Sweden, France, England, and Japan. *N Engl J Med* 1995;333:1232-5.

2 Katz S, Branch LG, Branson MH, Papsidero JA, Beck JC, Greer DS. Active life expectancy. *N Engl J Med* 1983;309:1218-24.

3 Branch LG, Guralnik JM, Foley DJ, Kohout FJ, Wetle TT, Ostfeld A, Katz S. Active life expectancy for 10,000 Caucasian men and women in three communities. *J Gerontol* 1991;46(4):M145-50.

4 Katz S, Ford AB, Moskowitz RW, Jackson BA, Jaffe MW. Studies of illness in the aged. The index of ADL: a standardized measure of biological and psychosocial function. *JAMA* 1963;185:94-9.

5 Rosow I, Breslau N. A Guttman health scale for the aged. *J Gerontol* 1966;21:556-9.

6 Branch LG, Jette AM. A prospective study of long-term care institutionalization among the aged. *Am J Public Health* 1982 December;72(12):1373-9.

7 Newman AB, Simonsick EM, Naydeck EM, Kritchevsky SB, Nevitt M, Pahor M, Satterfield S, Brach JS, Studenski SA, Harris TB. Association of long-distance corridor walk performance with mortality, cardiovascular disease, mobility limitation, and disability. *JAMA* 2006;295(17):2018-26.

8 LaCroix AZ, Guralnik JM, Berkman LF, Wallace RB, Satterfield S. Maintaining mobility in late life. II. Smoking, alcohol consumption, physical activity, and body mass index. *Am J Epidemiol* 1993;137:858-69.

9 Guralnik JM, LaCroix AZ, Abbott RD, Berkman LF, Satterfield S, Evans DA, Wallace RB. Maintaining mobility in late life. I. Demographic characteristics and chronic conditions. *Am J Epidemiol* 1993;137:845-57.

10 Mor V, Wilcox V, Rakowski W, Hiris J. Functional transitions among the elderly: patterns, predictors, and related hospital use. *Am J Public Health* 1994 August;84(8):1274-80.

11 Guralnik JM, LaCroix AZ, Branch LG, Kasl SV, Wallace RB. Morbidity and disability in older persons in the years prior to death. *Am J Public Health* 1991;81(4):443-7.

12 Foley DJ, Ostfeld AM, Branch LG, Wallace RB, McGloin J, Cornoni-Huntley JC. The risk of nursing home admission in three communities. *J Aging Health* 1992 May;4(2):155-73.

13 Ferrucci L, Guralnik JM, Pahor M, Corti MC, Havlik RJ. Hospital diagnoses, Medicare charges and nursing home admissions in the year when older persons become severely disabled. *JAMA* 1997;277:728-34.

14 Fried LP, Guralnik JM. Disability in older adults: evidence regarding significance,

etiology, and risk. *J Am Geriatr Soc* 1997;45(1):92-100.

15 Hyatt RH, Whitelaw MN, Bhat A, Scott S, Maxwell JD. Association of muscle strength with functional status of elderly people. *AGE AGEING* 1990;19(5):330-6.

16 Brill PA, Macera CA, Davis DR, Blair SN, Gordon N. Muscular strength and physical function. *Med Sci Sports Exerc* 2000 February;32(2):412-6.

17 Huang Y, Macera CA, Blair SN, Brill PA, Kohl HW, III, Kronenfeld JJ. Physical fitness, physical activity, and functional limitation in adults aged 40 and older. *Med Sci Sports Exerc* 1998 September;30(9):1430-5.

18 Gardner MM, Robertson MC, Campbell AJ. Exercise in preventing falls and fall related injuries in older people: a review of randomised controlled trials. *Br J Sports Med* 2000 February;34(1):7-17.

19 Gillespie LD, Gillespie WJ, Robertson MC, Lamb SE, Cumming RG, Rowe BH. Interventions for preventing falls in elderly people. *Cochrane Database Syst Rev* 2003;(4):CD000340.

20 Gillespie LD, Gillespie WJ, Robertson MC, Lamb SE, Cumming RG, Rowe BH. Interventions for preventing falls in elderly people. *Cochrane Database Syst Rev* 2001;(3):CD000340.

21 Tinetti ME, Baker DI, McAvay G, Claus EB, Garrett P, Gottschalk M, Koch ML, Trainor K, Horwitz RI. A multifactorial intervention to reduce the risk of falling among elderly people living in the community. *N Engl J Med* 1994 September 29;331(13):821-7.

22 Feskanich D, Willett W, Colditz G. Walking and leisure-time activity and risk of hip fracture in postmenopausal women. *JAMA* 2002 November 13;288(18):2300-6.

23 Uusi-Rasi K, Kannus P, Cheng S, Sievanen H, Pasanen M, Heinonen A, Nenonen A, Halleen J, Fuerst T, Genant H, Vuori I. Effect of alendronate and exercise on bone and physical performance of postmenopausal women: a randomized controlled trial. *Bone* 2003 July;33(1):132-43.

24 Rodriguez BL, Curb JD, Burchfiel CM, Abbott RD, Petrovitch H, Masaki K, Chiu D. Physical activity and 23-year incidence of coronary heart disease morbidity and mortality among middle-aged men. The Honolulu Heart Program. *Circulation* 1994 June;89(6):2540-4.

25 Hu FB, Sigal RJ, Rich-Edwards JW, Colditz GA, Solomon CG, Willett WC, Speizer FE, Manson JE. Walking compared with vigorous physical activity and risk of type 2 diabetes in women: a prospective study. *JAMA* 1999 October;%20;282(15):1433-9.

26 Mador MJ, Bozkanat E, Aggarwal A, Shaffer M, Kufel TJ. Endurance and strength training in patients with COPD. *Chest* 2004 June;125(6):2036-45.

27 Holmes MD, Chen WY, Feskanich D, Kroenke CH, Colditz GA. Physical activity and survival after breast cancer diagnosis. *JAMA* 2005 May 25;293(20):2479-86.

28 Manson JE, Nathan DM, Krolewski AS, Stampfer MJ, Willett WC, Hennekens CH. A prospective study of exercise and incidence of diabetes among US male physicians. *JAMA* 1992;268:63-7.

29 Manson JE, Rimm EB, Stampfer MJ, Colditz GA, Willett WC, Krolewski AS, Rosner B, Hennekens CH, Speizer FE. Physical activity and incidence of non-insulin-dependent diabetes mellitus in women. *Lancet* 1991 September 28;338(8770):774-8.

30 Heinonen A, Kannus P, Sievanen H, Oja P, Pasanen M, Rinne M, Uusi-Rasi K, Vuori I. Randomised controlled trial of effect of high-impact exercise on selected risk factors for osteoporotic fractures. *Lancet* 1996 November 16;348(9038):1343-7.

31 Villareal DT, Binder EF, Yarasheski KE, Williams DB, Brown M, Sinacore DR, Kohrt WM. Effects of exercise training added to ongoing hormone replacement therapy on bone mineral density in frail elderly women. *J Am Geriatr Soc* 2003 July;51(7):985-90.

32 Kohrt WM. Aging and the osteogenic response to mechanical loading. *Int J Sport Nutr Exerc Metab* 2001 December;11 Suppl:S137-42.:S137-S142.

33 Littman AJ, Kristal AR, White E. Effects of physical activity intensity, frequency, and activity type on 10-y weight change in middle-aged men and women. *Int J Obes (Lond)* 2005 May;29(5):524-33.

34 Nelson ME, Fiatarone MA, Morganti CM, Trice I, Greenberg RA, Evans WJ. Effects of high-intensity strength training on multiple risk factors for osteoporotic fractures. A randomized controlled trial. *JAMA* 1994 December 28;272(24):1909-14.

35 King AC, Oman RF, Brassington GS, Bliwise DL, Haskell WL. Moderate-intensity exercise and self-rated quality of sleep in older adults. A randomized controlled trial. *JAMA* 1997 January 1;277(1):32-7.

36 Casaburi R, ZuWallack R. Pulmonary rehabilitation for management of chronic obstructive pulmonary disease. *N Engl J Med* 2009 March 26;360(13):1329-35.

37 Tretli S, Hernes E, Berg JP, Hestvik UE, Robsahm TE. Association between serum 25(OH)D and death from prostate cancer. *Br J Cancer* 2009 February 10;100(3):450-4.

38 Ries AL, Bauldoff GS, Carlin BW, Casaburi R, Emery CF, Mahler DA, Make B, Rochester CL, ZuWallack R, Herrerias C. Pulmonary Rehabilitation: Joint ACCP/AACVPR Evidence-Based Clinical Practice Guidelines. *Chest* 2007 May;131(5 Suppl):4S-42S.

39 Carlson MC, Fried LP, Xue QL, Bandeen-Roche K, Zeger SL, Brandt J. Association between executive attention and physical functional performance in community-dwelling older women. *J Gerontol B Psychol Sci Soc Sci* 1999 September;54(5):S262-S270.

40 Buchner DM, Beresford SA, Larson EB, LaCroix AZ, Wagner EH. Effects of physical activity on health status in older adults. II. Intervention studies. *Annu Rev Public*

Health 1992;13:469-88.

41 Stuck AE, Walthert J, Nikolaus T, Büla CJ, Hohmann C, Beck JC. Risk factors for functional status decline in community-dwelling elderly people: a systematic literature review. *Soc Sci Med* 1999;48:445-69.

42 Leveille SG, Guralnik JM, Ferrucci L, Langlois JA. Aging successfully until death in old age: opportunities for increasing active life expectancy. *Am J Epidemiol* 1999 April 1;149(7):654-64.

43 Hubert HB, Bloch DA, Oehlert JW, Fries JF. Lifestyle habits and compression of morbidity. *J Gerontol A Biol Sci Med Sci* 2002 June;57(6):M347-M351.

44 Ferrucci L, Izmirlian G, Leveille S, Phillips CL, Corti MC, Brock DB, Guralnik JM. Smoking, physical activity, and active life expectancy. *Am J Epidemiol* 1999 April 1;149(7):645-53.

45 Strawbridge WJ, Cohen RD, Shema SJ, Kaplan GA. Successful aging: predictors and associated activities. *Am J Epidemiol* 1996 July 15;144(2):135-41.

46 Wu SC, Leu SY, Li CY. Incidence of and predictors for chronic disability in activities of daily living among older people in Taiwan. *J Am Geriatr Soc* 1999 September;47(9):1082-6.

47 Penninx BW, Leveille S, Ferrucci L, van Eijk JT, Guralnik JM. Exploring the effect of depression on physical disability: longitudinal evidence from the Established Populations for Epidemiologic Studies of the Elderly. *Am J Public Health* 1999 September;89(9):1346-52.

48 Rantanen T, Era P, Heikkinen E. Physical activity and the changes in maximal isometric strength in men and women from the age of 75 to 80 years. *J Am Geriatr Soc* 1997 December;45(12):1439-45.

49 Miszko TA, Cress ME, Slade JM, Covey CJ, Agrawal SK, Doerr CE. Effect of strength and power training on physical function in community-dwelling older adults. *J Gerontol A Biol Sci Med Sci* 2003 February;58(2):171-5.

50 Gauchard GC, Gangloff P, Jeandel C, Perrin PP. Influence of regular proprioceptive and bioenergetic physical activities on balance control in elderly women. *J Gerontol A Biol Sci Med Sci* 2003 September;58(9):M846-M850.

51 Nelson ME, Layne JE, Bernstein MJ, Nuernberger A, Castaneda C, Kaliton D, Hausdorff J, Judge JO, Buchner DM, Roubenoff R, Fiatarone Singh MA. The effects of multidimensional home-based exercise on functional performance in elderly people. *J Gerontol A Biol Sci Med Sci* 2004 February;59(2):154-60.

52 Ettinger WH, Burns R, Messier SP, Applegate WB, Rejeski WJ, Morgan T, Shumaker S, Berry MJ, O'Toole M, Monu J, Craven T. The Fitness Arthritis and Seniors Trial (FAST): a randomized trial comparing aerobic exercise and resistance exercise to a health education program on physical disability in older people with knee osteoarthritis. *JAMA* 1997;277:25-31.

53 Brown M, Sinacore DR, Ehsani AA, Binder EF, Holloszy JO, Kohrt WM. Low-intensity exercise as a modifier of physical frailty in older adults. *Arch Phys Med Rehabil* 2000 July;81(7):960-5.

54 Dolansky MA, Moore SM. Effects of cardiac rehabilitation on the recovery outcomes of older adults after coronary artery bypass surgery. *J Cardiopulm Rehabil* 2004 July;24(4):236-44.

55 Penninx BW, Messier SP, Rejeski WJ, Williamson JD, Di Bari M, Cavazzini C, Applegate WB, Pahor M. Physical exercise and prevention of ADL disability in older persons with osteoarthritis. *Arch Intern Med* 2001;161:2309-16.

56 Messier SP, Royer TD, Craven TE, O'Toole ML, Burns R, Ettinger WH, Jr. Long-term exercise and its effect on balance in older, osteoarthritic adults: results from the Fitness, Arthritis, and Seniors Trial (FAST). *J Am Geriatr Soc* 2000 February;48(2):131-8.

57 Berry MJ, Rejeski WJ, Adair NE, Zaccaro D. Exercise rehabilitation and chronic obstructive pulmonary disease stage. *Am J Respir Crit Care Med* 1999 October;160(4):1248-53.

58 Meyer K, Schwaibold M, Westbrook S, Beneke R, Hajric R, Lehmann M, Roskamm H. Effects of exercise training and activity restriction on 6-minute walking test performance in patients with chronic heart failure. *Am Heart J* 1997 April;133(4):447-53.

59 Meyer K, Schwaibold M, Westbrook S, Beneke R, Hajric R, Gornandt L, Lehmann M, Roskamm H. Effects of short-term exercise training and activity restriction on functional capacity in patients with severe chronic congestive heart failure. *Am J Cardiol* 1996 November 1;78(9):1017-22.

60 Fiatarone MA, O'Neill EF, Ryan ND, Clements KM, Solares GR, Nelson ME, Roberts SB, Kehayias JJ, Lipsitz LA, Evans WJ. Exercise training and nutritional supplementation for physical frailty in very elderly people. *N Engl J Med* 1994 June 23;330(25):1769-75.

61 Fiatarone MA, Marks EC, Ryan ND, Meredith CN, Lipsitz LA, Evans WJ. High-intensity strength training in nonagenarians (effects on skeletal muscle). *JAMA* 1990;263:3029-34.

62 Paillard T, Lafont C, Costes-Salon MC, Riviere D, Dupui P. Effects of brisk walking on static and dynamic balance, locomotion, body composition, and aerobic capacity in ageing healthy active men. *Int J Sports Med* 2004 October;25(7):539-46.

63 Asikainen TM, Miilunpalo S, Oja P, Rinne M, Pasanen M, Vuori I. Walking trials in postmenopausal women: effect of one vs two daily bouts on aerobic fitness. *Scand J Med Sci Sports* 2002 April;12(2):99-105.

64 Cress ME, Buchner DM, Questad KA, Esselman PC, deLateur BJ, Schwartz RS. Exercise: effects on physical functional performance in independent older adults. *J Gerontol A Biol Sci Med Sci* 1999 May;54(5):M242-M248.

65 Morganti CM, Nelson ME, Fiatarone MA, Dallal GE, Economos CD, Crawford BM, Evans WJ. Strength improvements with 1 yr of progressive resistance training in older women. *Med Sci Sports Exerc* 1995 June;27(6):906-12.

66 Ades PA, Ballor DL, Ashikaga T, Utton JL, Nair KS. Weight training improves walking endurance in healthy elderly persons. *Ann Intern Med* 1996 March 15;124(6):568-72.

67 Wolfson L, Whipple R, Derby C, Judge J, King M, Amerman P, Schmidt J, Smyers D. Balance and strength training in older adults: intervention and Tai Chi maintenance. *J Am Geriatr Soc* 1996;44:498-506.

68 Lord SR, Ward JA, Williams P, Strudwick M. The effect of a 12-month exercise trial on balance, strength, and falls in older women. *J Am Geriatr Soc* 1995;43:1198-206.

69 Jette AM, Harris BA, Sleeper L, Lachman ME, Heislein D, Giorgetti M, Levenson C. A home-based exercise program for nondisabled older adults. *J Am Geriatr Soc* 1996 June;44(6):644-9.

70 Cotman CW, Berchtold NC. Exercise: a behavioral intervention to enhance brain health and plasticity. *Trends Neurosci* 2002 June;25(6):295-301.

71 Abbott RD, White LR, Ross GW, Masaki KH, Curb JD, Petrovitch H. Walking and dementia in physically capable elderly men. *JAMA* 2004 September 22;292(12):1447-53.

72 Kramer AF, Hahn S, Cohen NJ, Banich MT, McAuley E, Harrison CR, Chason J, Vakil E, Bardell L, Boileau RA, Colcombe A. Ageing, fitness and neurocognitive function. *Nature* 1999 July 29;400(6743):418-9.

73 Province MA, Hadley EC, Hornbrook MC, Lipsitz LA, Miller JP, Mulrow CD, Ory MG, Sattin RW, Tinetti ME, Wolf SL. The effects of exercise on falls in elderly patients. A preplanned meta-analysis of the FICSIT Trials. Frailty and Injuries: Cooperative Studies of Intervention Techniques. *JAMA* 1995 May 3;273(17):1341-7.

74 Chang JT, Morton SC, Rubenstein LZ, Mojica WA, Maglione M, Suttorp MJ, Roth EA, Shekelle PG. Interventions for the prevention of falls in older adults: systematic review and meta-analysis of randomised clinical trials. *BMJ* 2004 March 20;328(7441):680.

75 Kannus P, Sievanen H, Palvanen M, Jarvinen T, Parkkari J. Prevention of falls and consequent injuries in elderly people. *Lancet* 2005 November 26;366(9500):1885-93.

76 King MB, Tinetti ME. Falls in community-dwelling older persons. *J Am Geriatr Soc* 1995 October;43(10):1146-54.

77 Physical Activity and Health: A Report of the Surgeon General. Atlanta, GA: U.S. Department of Health and Human Services. Department of Health and Human Services, Centers for Disease Control and Prevention, National Center for Chronic Disease Prevention and Health Promotion; 1996.

78 Morrow JR, Jr., Jackson AW, Bazzarre TL, Milne D, Blair SN. A one-year follow-up

to physical activity and health. A report of the Surgeon General. *Am J Prev Med* 1999 July;17(1):24-30.

79 SHEP Cooperative Research Group. Prevention of stroke by antihypertensive drug treatment in older persons with isolated systolic hypertension. Final results of the Systolic Hypertension in the Elderly Program (SHEP). *JAMA* 1991;265:3255-64.

80 Di Bari M, Pahor M, Franse LV, Shorr RI, Ferrucci L, Wan JY, Somes GW, Applegate WB. Dementia and disability outcomes in large hypertension trials: lessons learned from the Systolic Hypertension in the Elderly Program (SHEP) Trial. *Am J Epidemiol* 2001;153:72-8.

81 Applegate WB, Pressel S, Wittes J, Luhr J, Shekelle RB, Camel GH, Greenlick MR, Hadley E, Moye L, Perry HM, Jr., et al. Impact of the treatment of isolated systolic hypertension on behavioral variables. Results from the systolic hypertension in the elderly program. *Arch Intern Med* 1994;154(19):2154-60.

82 Rudman D, Feller AG, Nagraj HS, Gergans GA, Lalitha PY, Goldberg AF, Schlenker RA, Cohn L, Rudman IW, Mattson DE. Effects of human growth hormone in men over 60 years old. *N Engl J Med* 1990;323:1-6.

83 Bean JF, Vora A, Frontera WR. Benefits of exercise for community-dwelling older adults. *Arch Phys Med Rehabil* 2004 July;85(7 Suppl 3):S31-S42.

84 The Cardiac Arrhythmia Suppression Trial (CAST). Investigators preliminary report: effect of encainide and flecainide on mortality in a randomized trial of arrhythmia suppression after myocardial infarction. *N Engl J Med* 1989;321:406-12.

85 The ALLHAT Collaborative Research Group. Major outcomes in high-risk hypertensive patients randomized to angiotensin-converting enzyme Inhibitor or calcium channel blocker vs diuretic. The Antihypertensive and Lipid-Lowering Treatment to Prevent Heart Attack Trial (ALLHAT). *JAMA* 2002;288:2981-97.

86 Hulley S, Grady D, Bush T, Furberg C, Herrington D, Riggs B, Vittinghoff E. Randomized trial of estrogen plus progestin for secondary prevention of coronary heart disease in postmenopausal women. Heart and Estrogen/progestin Replacement Study (HERS) Research Group. *JAMA* 1998 August;19;280(7):605-13.

87 Writing Group for the Women's Health Initiative Investigators. Risks and Benefits of Estrogen Plus Progestin in Healthy Postmenopausal Women; Principal Results From the Women's Health Initiative Randomized Controlled Trial. *JAMA* 2002;288:321-33.

88 LIFE investigators, Pahor M, Blair SN, Espeland M, Fielding RA, Gill TM, Guralnik JM, Hadley EC, King AC, Kritchevsky SB, Maraldi C, Miller ME, Newman AB, Rejeski WJ, Romaskan S et al. Effects of a Physical Activity Intervention on Measures of Physical Performance: Results of the Lifestyle Interventions and Independence for Elders Pilot (LIFE-P) Study. *J Gerontol A Biol Sci Med Sci* 2006 November;61(11):1157-65.

89 Rejeski WJ, Fielding RA, Blair SN, Guralnik JM, Gill TM, Hadley EC, King AC, Kritchevsky SB, Miller ME, Newman AB, Pahor M. The Lifestyle Interventions and

Independence for Elders (LIFE) Pilot Study: Design and Methods. *Contemp Clin Trials* 2005;26(2):141-54.

90 Guralnik JM, Ferrucci L, Pieper CF, Leveille SG, Markides KS, Ostir GV, Studenski S, Berkman LF, Wallace RB. Lower extremity function and subsequent disability: consistency across studies, predictive models, and value of gait speed alone compared with the short physical performance battery. *J Gerontol A Biol Sci Med Sci* 2000 April;55(4):M221-M231.

91 Hoxie RR, Rubenstein LZ. Are older pedestrians allowed enough time to cross intersections safely? *J Am Geriatr Soc* 2002;42:241-4.

92 King AC, Rejeski WJ, Buchner DM. Physical activity interventions targeting older adults. A critical review and recommendations. *Am J Prev Med* 1998 November;15(4):316-33.

93 Fielding RA, LeBrasseur NK, Cuoco A, Bean J, Mizer K, Fiatarone Singh MA. High-velocity resistance training increases skeletal muscle peak power in older women. *J Am Geriatr Soc* 2002 April;50(4):655-62.

94 Fletcher GF, Balady GJ, Amsterdam EA, Chaitman B, Eckel R, Fleg J, Froelicher VF, Leon AS, Pina IL, Rodney R, Simons-Morton DA, Williams MA, Bazzarre T. Exercise standards for testing and training: a statement for healthcare professionals from the American Heart Association. *Circulation* 2001 October 2;104(14):1694-740.

95 American College of Sports Medicine Position Stand. Exercise and physical activity for older adults. *Med Sci Sports Exerc* 1998 June;30(6):992-1008.

96 Borg G. *Perceived exertion and pain scales*. Champaign IL: Human Kinetics; 1988.

97 Rejeski WJ, Martin KA, Miller ME, Ettinger WH, Jr., Rapp S. Perceived importance and satisfaction with physical function in patients with knee osteoarthritis. *Ann Behav Med* 1998;20(2):141-8.

98 Shumaker SA, Legault C, Kuller L, Rapp SR, Thal L, Lane DS, Fillit H, Stefanick ML, Hendrix SL, Lewis CE, Masaki K, Coker LH. Conjugated equine estrogens and incidence of probable dementia and mild cognitive impairment in postmenopausal women: Women's Health Initiative Memory Study. *JAMA* 2004 June 23;291(24):2947-58.

99 DeKosky ST, Fitzpatrick A, Ives DG, Saxton J, Williamson J, Lopez OL, Burke G, Fried L, Kuller LH, Robbins J, Tracy R, Woolard N, Dunn L, Kronmal R, Nahin R et al. The Ginkgo Evaluation of Memory (GEM) study: Design and baseline data of a randomized trial of Ginkgo biloba extract in prevention of dementia. *Contemp Clin Trials* 2006 June;27(3):238-53.

100 Kramer AF, Colcombe SJ, McAuley E, Scalf PE, Erickson KI. Fitness, aging and neurocognitive function. *Neurobiol Aging* 2005 December;26 Suppl 1:124-7.

101 Colcombe SJ, Kramer AF, McAuley E, Erickson KI, Scalf P. Neurocognitive aging and cardiovascular fitness: recent findings and future directions. *J Mol Neurosci*

2004;24(1):9-14.

102 Colcombe S, Kramer AF. Fitness effects on the cognitive function of older adults: A meta-analytic study. *Psychol Sci* 2003;14(2):125-30.

103 Kuslansky G, Katz M, Verghese J, Hall CB, Lapuerta P, LaRuffa G, Lipton RB. Detecting dementia with the Hopkins Verbal Learning Test and the Mini-Mental State Examination. *Arch Clin Neuropsychol* 2004 January;19(1):89-104.

104 Espeland MA, Rapp SR, Robertson JI, Granek I, Murphy C, Albert M, Bassford T. Benchmarks for designing two-stage studies using Modified Mini-Mental State Examinations: experience from the Women's Health Initiative Memory Study. *Clin Trials* 2006;3:99-106.

105 Espeland MA, Gill TM, Guralnik JM, Miller ME, Fielding RA, Newman AB, Pahor M. Designing Clinical Trials of Intervention for Mobility Disability: results from the Lifestyle Interventions and Independence for Elders (LIFE) Pilot trial. *J Gerontol A Biol Sci Med Sci* 2007;62(11):1237-43.

106 Geda YE, Knopman DS, Mrazek DA, Jicha GA, Smith GE, Negash S, Boeve BF, Ivnik RJ, Petersen RC, Pankratz VS, Rocca WA. Depression, apolipoprotein E genotype, and the incidence of mild cognitive impairment: a prospective cohort study. *Arch Neurol* 2006 March;63(3):435-40.

107 Launer LJ, Andersen K, Dewey ME, Letenneur L, Ott A, Amaducci LA, Brayne C, Copeland JR, Dartigues JF, Kragh-Sorensen P, Lobo A, Martinez-Lage JM, Stijnen T, Hofman A. Rates and risk factors for dementia and Alzheimer's disease: results from EURODEM pooled analyses. EURODEM Incidence Research Group and Work Groups. European Studies of Dementia. *Neurology* 1999 January 1;52(1):78-84.

108 Palmer K, Wang HX, Backman L, Winblad B, Fratiglioni L. Differential evolution of cognitive impairment in nondemented older persons: results from the Kungsholmen Project. *Am J Psychiatry* 2002 March;159(3):436-42.

109 Hendrie H. Personal communication. 2006.

110 Williamson JD. Personal communication. 2006.

111 Griffith KA, Sherrill DL, Siegel EM, Manolio TA, Bonekat HW, Enright PL. Predictors of loss of lung function in the elderly: the Cardiovascular Health Study. *Am J Respir Crit Care Med* 2001 January;163(1):61-8.

112 Ferrucci L, Penninx BWJH, Leveille SG, Corti MC, Pahor M, Wallace RB, Harris TB, Guralnik JM. Characteristics of non-disabled older persons who perform poorly in objective tests of lower extremity function. *J Am Geriatr Soc* 2000;48:1102-10.

113 Guralnik JM, Ferrucci L, Penninx BW, Kasper JD, Leveille SG, Bandeen-Roche K, Fried LP. New and worsening conditions and change in physical and cognitive performance during weekly evaluations over 6 months: the Women's Health and Aging Study. *J Gerontol A Biol Sci Med Sci* 1999 August;54(8):M410-M422.

114 Guralnik JM, Ferrucci L, Simonsick EM, Salive ME, Wallace RB. Lower-extremity function in persons over the age of 70 years as a predictor of subsequent disability. *N Engl J Med* 1995;332(9):556-61.

115 Guralnik JM, Seeman TE, Tinetti ME, Nevitt MC, Berkman LF. Validation and use of performance measures of functioning in a non-disabled older population: MacArthur studies of successful aging. *Aging Milano* 1994;6(6):410-9.

116 Guralnik JM, Simonsick EM, Ferrucci L, Glynn RJ, Berkman LF, Blazer DG, Scherr PA, Wallace RB. A short physical performance battery assessing lower extremity function: association with self-reported disability and prediction of mortality and nursing home admission. *J Gerontol* 1994;49(2):M85-94.

117 Ostir G, Volpato S, Fried LP, Chaves PH, Guralnik JM. Reliability and sensitivity to change assessed for a summary measure of lower body function: Results from the Women's Health and Aging Study. *J Clin Epidemiol* 2002;55:916-21.

118 Penninx BW, Ferrucci L, Leveille SG, Rantanen T, Pahor M, Guralnik JM. Lower extremity performance in nondisabled older persons as a predictor of subsequent hospitalization. *J Gerontol A Biol Sci Med Sci* 2000;55A:M691-M697.

119 Rantanen T, Harris T, Leveille SG, Visser M, Foley D, Masaki K, Guralnik JM. Muscle strength and body mass index as long-term predictors of mortality in initially healthy men. *J Gerontol A Biol Sci Med Sci* 2000 March;55(3):M168-M173.

120 Rantanen T, Guralnik JM, Foley D, Masaki K, Leveille S, Curb JD, White L. Midlife hand grip strength as a predictor of old age disability. *JAMA* 1999 February 10;281(6):558-60.

121 World Health Organization. *World Health Organization: International classification of impairment, disability and handicaps.* Geneva: 2001.

122 Rejeski WJ, Ip EH, Marsh AP, Miller ME, Farmer DF. Measuring disability in older adults: the International Classification System of Functioning, Disability and Health (ICF) framework. *Geriatr Gerontol Int* 2008 March;8(1):48-54.

123 Rejeski WJ, Ip EH, Marsh AP, Zhang Q, Miller ME. Obesity influences transitional states of disability in older adults with knee pain. *Arch Phys Med Rehabil* 2008 November;89(11):2102-7.

124 Stewart AL, Verboncoeur CJ, McLellan BY, Gillis DE, Rush S, Mills KM, King AC, Ritter P, Brown BW, Jr., Bortz WM. Physical activity outcomes of CHAMPS II: a physical activity promotion program for older adults. *J Gerontol A Biol Sci Med Sci* 2001 August;56(8):M465-M470.

125 Stewart AL, Mills KM, King AC, Haskell WL, Gillis D, Ritter PL. CHAMPS physical activity questionnaire for older adults: outcomes for interventions. *Med Sci Sports Exerc* 2001 July;33(7):1126-41.

126 Psaty BM, Lee M, Savage PJ, Rutan GH, German PS, Lyles M. Assessing the use of medications in the elderly: methods and initial experience in the Cardiovascular

Health Study. The Cardiovascular Health Study Collaborative Research Group. *J Clin Epidemiol* 1992;45:683-92.

127 Wechsler D. *WAIS-R manual*. New York: Psychological Corporation; 1981.

128 Salthouse TA. The role of memory in the age decline in digit-symbol substitution performance. *J Gerontol* 1978 March;33(2):232-8.

129 Brandt J, Benedict RHB. *Hopkins Verbal Learning Test-Revised. Professional manual*. Lutz, FL: Psychological Assessment Resources, Inc; 2001.

130 Teng EL, Chui HC. The Modified Mini-Mental State (3MS) examination. *J Clin Psychiatry* 1987 August;48(8):314-8.

131 Becker JT, Boller F, Saxton J, Gonigle-Gibson KL. Normal rates of forgetting of verbal and non-verbal material in Alzheimer's disease. *Cortex* 1987 March;23(1):59-72.

132 Eriksen BA, Eriksen CW. Effects of noise letters upon the identification of a target letter in a nonsearch task. *Perception & Psychophysics* 1974;16:143-9.

133 Dobbs AR, Rule BG. Adult age differences in working memory. *Psychol Aging* 1989 December;4(4):500-3.

134 Jennings JM, Webster LM, Kleykamp BA, Dagenbach D. Recollection training and transfer effects in older adults: Successful use of a repetition-lag procedure. *Aging, Neuropsy-chology, and Cognition* 2005;12:278-98.

135 Cepeda NJ, Kramer AF, Gonzalez de Sather JC. Changes in executive control across the life span: examination of task-switching performance. *Dev Psychol* 2001 September;37(5):715-30.

136 Kramer AF, Cepeda NJ, Cepeda ML. Methylphenidate effects on task-switching performance in attention-deficit/hyperactivity disorder. *J Am Acad Child Adolesc Psychiatry* 2001 November;40(11):1277-84.

137 Pfeffer RI, Kurosaki TT, Harrah CH, Jr., Chance JM, Filos S. Measurement of functional activities in older adults in the community. *J Gerontol* 1982 May;37(3):323-9.

138 Radloff LS. The CES-D Scale: A self-report depression scale for research in the general population. *Appl Psychol Measurement* 1977;1(3):385-401.

139 Kohout FJ, Berkman LF, Evans DA, Cornoni-Huntley J. Two shorter forms of the CES-D (Center for Epidemiological Studies Depression) depression symptoms index. *J Aging Health* 1993 May;5(2):179-93.

140 Rejeski WJ, Reboussin BA, Dunn AL, King AC. A modified exercise induced feeling inventory for chronic training and baseline profiles of participants in the activity counseling trial (ACT). *J Health Psychol* 1999;4:97-108.

141 Cohen S, Kamarck T, Mermelstein R. A global measure of perceived stress. *J Health Soc Behav* 1983;24(4):385-96.

142 Cohen S, Williamson GM. Perceived stress in a probability sample of the United States. In: S.Spacapan & S.Oakamp, editor. *The Social Psychology of Health*.Newbury Park, CA: Sage; 1988.

143 Ramsey SD, Sullivan SD, Kaplan RM, Wood DE, Chiang YP, Wagner JL. Economic analysis of lung volume reduction surgery as part of the National Emphysema Treatment Trial. NETT Research Group. *Ann Thorac Surg* 2001 March;71(3):995-1002.

144 Kaplan RM, Atkins CJ, Timms R. Validity of a quality of well-being scale as an outcome measure in chronic obstructive pulmonary disease. *J Chronic Dis* 1984;37(2):85-95.

145 Kaplan RM, Anderson JP, Patterson TL, McCutchan JA, Weinrich JD, Heaton RK, Atkinson JH, Thal L, Chandler J, Grant I. Validity of the Quality of Well-Being Scale for persons with human immunodeficiency virus infection. HNRC Group. HIV Neurobehavioral Research Center. *Psychosom Med* 1995 March;57(2):138-47.

146 Orenstein DM, Kaplan RM. Measuring the quality of well-being in cystic fibrosis and lung transplantation. The importance of the area under the curve. *Chest* 1991 October;100(4):1016-8.

147 Orenstein DM, Pattishall EN, Nixon PA, Ross EA, Kaplan RM. Quality of well-being before and after antibiotic treatment of pulmonary exacerbation in patients with cystic fibrosis. *Chest* 1990 November;98(5):1081-4.

148 Kaplan RM, Hartwell SL, Wilson DK, Wallace JP. Effects of diet and exercise interventions on control and quality of life in non-insulin-dependent diabetes mellitus. *J Gen Intern Med* 1987 July;2(4):220-8.

149 Ganiats TG, Palinkas LA, Kaplan RM. Comparison of Quality of Well-Being scale and Functional Status Index in patients with atrial fibrillation. *Med Care* 1992 October;30(10):958-64.

150 Squier HC, Ries AL, Kaplan RM, Prewitt LM, Smith CM, Kriett JM, Jamieson SW. Quality of well-being predicts survival in lung transplantation candidates. *Am J Respir Crit Care Med* 1995 December;152(6 Pt 1):2032-6.

151 Bombardier C, Ware J, Russell IJ, Larson M, Chalmers A, Read JL. Auranofin therapy and quality of life in patients with rheumatoid arthritis. Results of a multicenter trial. *Am J Med* 1986 October;81(4):565-78.

152 Kaplan RM. Decisions about prostate cancer screening in managed care. *Curr Opin Oncol* 1997 September;9(5):480-6.

153 Kaplan RM. Quality of life assessment for cost/utility studies in cancer. *Cancer Treat Rev* 1993;19 Suppl A:85-96.:85-96.

154 Patterson TL, Kaplan RM, Grant I, Semple SJ, Moscona S, Koch WL, Harris MJ, Jeste DV. Quality of well-being in late-life psychosis. *Psychiatry Res* 1996 July 31;63(2-3):169-81.

155 Kaplan RM. *Allocating health resources in California : learning from the Oregon experiment*. Berkeley, CA: 1993.

156 Ries AL, Kaplan RM, Myers R, Prewitt LM. Maintenance after pulmonary rehabilitation in chronic lung disease: a randomized trial. *Am J Respir Crit Care Med* 2003 March 15;167(6):880-8.

157 Johns MW. A new method for measuring daytime sleepiness: the Epworth sleepiness scale. *Sleep* 1991 December;14(6):540-5.

158 Bastien CH, Vallieres A, Morin CM. Validation of the Insomnia Severity Index as an outcome measure for insomnia research. *Sleep Med* 2001 July;2(4):297-307.

159 Buysse DJ, Reynolds CF, III, Monk TH, Hoch CC, Yeager AL, Kupfer DJ. Quantification of subjective sleep quality in healthy elderly men and women using the Pittsburgh Sleep Quality Index (PSQI). *Sleep* 1991 August;14(4):331-8.

160 Netzer NC, Stoohs RA, Netzer CM, Clark K, Strohl KP. Using the Berlin Questionnaire to identify patients at risk for the sleep apnea syndrome. *Ann Intern Med* 1999 October 5;131(7):485-91.

161 Borg GA. Psychophysical bases of perceived exertion. *Med Sci Sports Exerc* 1982;14(5):377-81.

162 Sampson RJ, Raudenbush SW, Earls F. Neighborhoods and violent crime: a multilevel study of collective efficacy. *Science* 1997 August 15;277(5328):918-24.

163 Cerin E, Saelens BE, Sallis JF, Frank LD. Neighborhood Environment Walkability Scale: validity and development of a short form. *Med Sci Sports Exerc* 2006 September;38(9):1682-91.

164 King AC, Baumann K, O'Sullivan P, Wilcox S, Castro C. Effects of moderate-intensity exercise on physiological, behavioral, and emotional responses to family caregiving: a randomized controlled trial. *J Gerontol A Biol Sci Med Sci* 2002 January;57(1):M26-M36.

165 King AC, Friedman R, Marcus B, Castro C, Forsyth L, Napolitano M, Pinto B. Harnessing motivational forces in the promotion of physical activity: The Community Health Advice by Telephone (CHAT) Project. *Health Education Research* 2002;17:627-36.

166 King AC, Haskell WL, Young DR, Oka RK, Stefanick ML. Long-term effects of varying intensities and formats of physical activity on participation rates, fitness, and lipoproteins in men and women aged 50 to 65 years. *Circulation* 1995 May 15;91(10):2596-604.

167 Marcus BH, Dubbert PM, Forsyth LH, McKenzie TL, Stone EJ, Dunn AL, Blair SN.

Physical activity behavior change: issues in adoption and maintenance. *Health Psychol* 2000 January;19(1 Suppl):32-41.

168 King AC, Pruitt LA, Phillips W, Oka R, Rodenburg A, Haskell WL. Comparative effects of two physical activity programs on measured and perceived physical functioning and other health-related quality of life outcomes in older adults. *J Gerontol A Biol Sci Med Sci* 2000 February;55(2):M74-M83.

169 Bassett DR, Jr., Ainsworth BE, Swartz AM, Strath SJ, O'Brien WL, King GA. Validity of four motion sensors in measuring moderate intensity physical activity. *Med Sci Sports Exerc* 2000 September;32(9 Suppl):S471-S480.

170 Welk GJ, Differding JA, Thompson RW, Blair SN, Dziura J, Hart P. The utility of the Digi-walker step counter to assess daily physical activity patterns. *Med Sci Sports Exerc* 2000 September;32(9 Suppl):S481-S488.

171 Marcus BH, Simkin LR. The transtheoretical model: applications to exercise behavior. *Med Sci Sports Exerc* 1994 November;26(11):1400-4.

172 Dishman RK, Sallis JF. Determinants and interventions for physical activity and exercise. In: Bouchard C, Sthephens T, editors. *Physical activity, fitness, and health: International proceedings and consensus statement*.Champaign, IL: Human Kinetics; 1994. p. 214-34.

173 The Writing Group for Activity Counseling Trial Research Group. Effects of physical activity counseling in primary care: the Activity Counseling Trial: a randomized controlled trial. *JAMA* 2001 August 8;286(6):677-87.

174 Skelton DA, Young A, Greig CA, Malbut KE. Effects of resistance training on strength, power, and selected functional abilities of women aged 75 and older. *J Am Geriatr Soc* 1995 October;43(10):1081-7.

175 Fiatarone MA. Elderly patients and frailty. *Resistance training for health and rehabilitation*.Champaign IL: Human Kinetics; 2001. p. 181-213.

176 Gordon NF, Kohl HW, III, Pollock ML, Vaandrager H, Gibbons LW, Blair SN. Cardiovascular safety of maximal strength testing in healthy adults. *Am J Cardiol* 1995 October 15;76(11):851-3.

177 McCartney N. The safety of resistance training: thermodynamic factors and cardiovascular incidents. *Resistance training for health and rehabilitation*.Champaign, IL: Human Kinetics; 2001. p. 83-94.

178 Gill TM, DiPietro L, Krumholz HM. Role of exercise stress testing and safety monitoring for older persons starting an exercise program. *JAMA* 2000 July;284(3):342-9.

179 American College of Sports Medicine. American College of Sports Medicine Position Stand. Exercise and physical activity for older adults. *Med Sci Sports Exerc* 1998 June;30(6):992-1008.

180 Hollenberg M, Ngo LH, Turner D, Tager IB. Treadmill exercise testing in an epidemiologic study of elderly subjects. *J Gerontol A Biol Sci Med Sci* 2002;53A:B259-B267.

181 Furberg CD, Manolio TA, Psaty BM, Bild DE, Borhani NO, Newman A, Tabatznik B, Rautaharju PM. Major electrocardiographic abnormalities in persons aged 65 years and older (the Cardiovascular Health Study). Cardiovascular Health Study Collaborative Research Group. *Am J Cardiol* 1992;69(16):1329-35.

182 *Exercise and the heart*. 4th ed. Philadelphia, PA: WB Saunders; 2000.

183 Hakim AA, Curb JD, Petrovitch H, Rodriguez BL, Yano K, Ross GW, White LR, Abbott RD. Effects of walking on coronary heart disease in elderly men: the Honolulu Heart Program. *Circulation* 1999 July 6;100(1):9-13.

184 Hakim AA, Petrovitch H, Burchfiel CM, Ross GW, Rodriguez BL, White LR, Yano K, Curb JD, Abbott RD. Effects of walking on mortality among nonsmoking retired men. *N Engl J Med* 1998 January 8;338(2):94-9.

185 Feeny D, Furlong W, Boyle M, Torrance GW. Multi-attribute health status classification systems. Health Utilities Index. *Pharmacoeconomics* 1995 June;7(6):490-502.

186 Gold MR. *Cost-effectiveness in health and medicine*. New York, Oxford: University Press; 1996.

187 Kaplan RM, Anderson J. The general health policy model: An integrated approach. In: Spilker B, editor. *Quality of Life and Pharmacoeconomics in Clinical Trials*.New York: Raven Press; 1996. p. 309-22.

188 Anderson JJ, Felson DT, Meenan RF, Williams HJ. Which traditional measures should be used in rheumatoid arthritis clinical trials? *Arthritis Rheum* 1989 September;32(9):1093-9.

189 Anderson JJ, Firschein HE, Meenan RF. Sensitivity of a health status measure to short-term clinical changes in arthritis. *Arthritis Rheum* 1989 July;32(7):844-50.

190 Balaban DJ, Sagi PC, Goldfarb NI, Nettler S. Weights for scoring the quality of well-being instrument among rheumatoid arthritics. A comparison to general population weights. *Med Care* 1986 November;24(11):973-80.

191 Coons SJ, Rao S, Keininger DL, Hays RD. A comparative review of generic quality-of-life instruments. *Pharmacoeconomics* 2000 January;17(1):13-35.

192 Sieber WJ, David KM, Adams JE, Kaplan RM, Ganiats TG. Assessing the impact of migraine on health-related quality of life: An additional use of the quality of well-being scale-self-administered. *Headache* 2000 September;40(8):662-71.

193 Kaplan RM, Alcaraz JE, Anderson JP, Weisman M. Quality-adjusted life years lost to arthritis: effects of gender, race,and social class. *Arthritis Care Res* 1996 December;9(6):473-82.

194 Kaplan RM. Two pathways to prevention. *Am Psychol* 2000 April;55(4):382-96.

195 Bachman DL, Wolf PA, Linn RT, Knoefel JE, Cobb JL, Belanger AJ, White LR, D'Agostino RB. Incidence of dementia and probable Alzheimer's disease in a general population: the Framingham Study. *Neurology* 1993 March;43(3 Pt 1):515-9.

196 Hebert LE, Scherr PA, Beckett LA, Albert MS, Pilgrim DM, Chown MJ, Funkenstein HH, Evans DA. Age-specific incidence of Alzheimer's disease in a community population. *JAMA* 1995 May 3;273(17):1354-9.

197 Cahn DA, Salmon DP, Butters N, Wiederholt WC, Corey-Bloom J, Edelstein SL, Barrett-Connor E. Detection of dementia of the Alzheimer type in a population-based sample: neuropsychological test performance. *J Int Neuropsychol Soc* 1995 May;1(3):252-60.

198 Mukamal KJ, Kuller LH, Fitzpatrick AL, Longstreth WT, Jr., Mittleman MA, Siscovick DS. Prospective study of alcohol consumption and risk of dementia in older adults. *JAMA* 2003 March 19;289(11):1405-13.

199 Shumaker SA, Reboussin BA, Espeland MA, Rapp SR, McBee WL, Dailey M, Bowen D, Terrell T, Jones BN. The Women's Health Initiative Memory Study (WHIMS): a trial of the effect of estrogen therapy in preventing and slowing the progression of dementia. *Control Clin Trials* 1998 December;19(6):604-21.

200 Black DM, Cummings SR, Karpf DB, Cauley JA, Thompson DE, Nevitt MC, Bauer DC, Genant HK, Haskell WL, Marcus R, Ott SM, Torner JC, Quandt SA, Reiss TF, Ensrud KE. Randomised trial of effect of alendronate on risk of fracture in women with existing vertebral fractures. Fracture Intervention Trial Research Group. *Lancet* 1996;348:1535-41.

201 Morris JC, Heyman A, Mohs RC, Hughes JP, van BG, Fillenbaum G, Mellits ED, Clark C. The Consortium to Establish a Registry for Alzheimer's Disease (CERAD). Part I. Clinical and neuropsychological assessment of Alzheimer's disease. *Neurology* 1989 September;39(9):1159-65.

202 Silverman JM, Breitner JC, Mohs RC, Davis KL. Reliability of the family history method in genetic studies of Alzheimer's disease and related dementias. *Am J Psychiatry* 1986 October;143(10):1279-82.

203 Kawas C, Segal J, Stewart WF, Corrada M, Thal LJ. A validation study of the Dementia Questionnaire. *Arch Neurol* 1994 September;51(9):901-6.

204 Ellis RJ, Jan K, Kawas C, Koller WC, Lyons KE, Jeste DV, Hansen LA, Thal LJ. Diagnostic validity of the dementia questionnaire for Alzheimer disease. *Arch Neurol* 1998 March;55(3):360-5.

205 Brown LM, Schinka JA, Mortimer JA, Graves AB. 3MS Normative Data for Elderly African Americans. *Journal of Clinical and Experimental Neuropsychology* 2003; 25(2): 234 — 241.

206 Jones TG, Schinka JA, Vanderploeg RD, Small BJ, Graves AB, Mortimer JA. 3MS normative data for the elderly. *Archives of Clinical Neuropsychology* 2002 Feb; 17(2): 171-7.

207 Bravo G, Hébert R. Age- and education-specific reference values for the Mini-Mental and modified Mini-Mental State Examinations derived from a non-demented elderly population. *Int J Geriatr Psychiatry* 1997 Oct;12(10):1008-18.

208 Callahan CM, Unverzagt FW, Hui SL, Perkins AJ, Hendrie HC**.** Six-item screener to identify cognitive impairment among potential subjects for clinical research. *Medical Care* 2002;40(9):771-81.

Appendix A

Diagnostic protocol for mild cognitive impairment (MCI) and dementia

As a tertiary aim, LIFE will determine the incidence of the combined outcome of Mild Cognitive Impairment (MCI) or Dementia. The design will involve a 2 stage approach at the 24-month and the PIV visits which will include the baseline screening battery (the LIFE Cognition baseline battery administered to all participants) followed by a more detailed neuropsychological assessment (see below) for incident MCI or dementia (See Figure). This approach to assessing for incident MCI/Dementia will be used at **24 months and any subsequent evaluations**.

At baseline, all participants will be classified as having no cognitive impairment or MCI (participants with diagnosed dementia will be excluded from the trial). MCI at baseline will be defined as the presence of abnormal baseline cognition scores (adjusted for age and education) in the presence of preserved daily function assessed with the Functional Assessment Questionnaire (FAQ).[137]

Diagnostic Protocol for Mild Cognitive Impairment and Dementia at Follow-Up

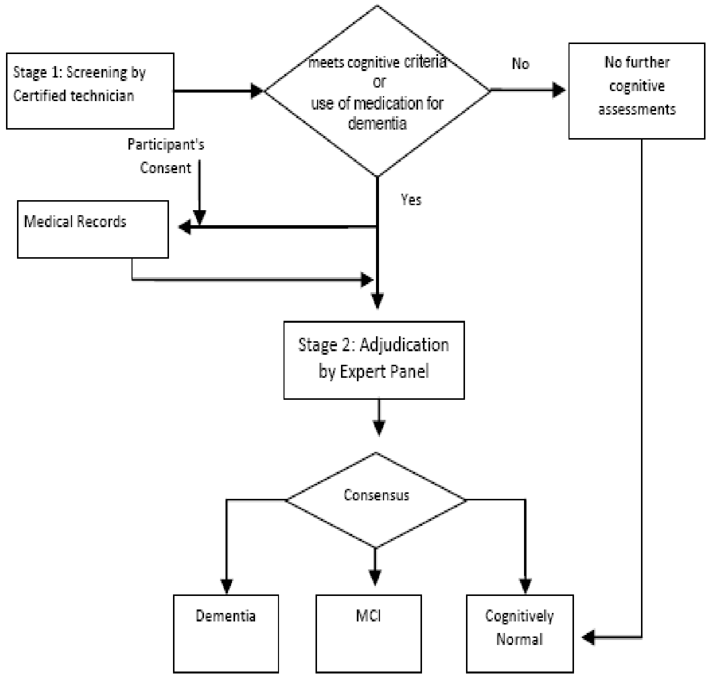

Additional Detailed Neuropsychological Tests

In addition to the cognitive battery components already administered to all LIFE participants at baseline ,24 month, and PIV visits, a certified technician will administer additional LIFE Neuropsychological Testing beginning at 24 months and thereafter as follows:

1. **Portions of the Consortium to Establish a Registry for Alzheimer's Disease (CERAD)**.[201] These include tests of **verbal fluency**, **naming**, and the **Trails A and B**. Normative values in a variety of populations and education groups are available for all these subtests.

2. **Center for Epidemiologic Studies Depression Scale (CES-D)**.[138] Depressive symptoms are assessed in the main trial with the 11-item version of the CES-D,[138] which queries about depressive symptoms experienced in the previous week. Scores are transformed using the procedure recommended by Kohout et al. to make it compatible with the full 20-item instrument.[139] Total scores range from 0 to 60, with higher scores indicating more depressive symptoms.

3. **The Functional Assessment Questionnaire.**[137] A brief, 10 item questionnaire administered to a family member, close friend or caregiver and has been validated for the purposes of ascertaining the impact of cognition on important daily functions. This will be obtained only for participants that score below the specified cutoff (see section 6.2.11).

Participants unable to undergo Face-To Face assessment
If participants cannot come to the clinic for their exams or if they reside in nursing homes, we will first attempt to make a home/nursing home visit for the appropriate evaluation, administer the cognitive battery, and review relevant hospital records and information from the physicians. For participants refusing both clinic and home visits, we will try to complete (by phone) the **Telephone Interview for Cognitive Status (TICS)** and compare these results to their baseline cognitive testing results. For participants who do not complete an in-person cognitive assessment or who score <30 on the TICS, field center technicians will administer an abbreviated version of the **Dementia Questionnaire (DQ)** to the proxy of the participant. Information regarding possible dementia will be referred for diagnostic adjudication by the LIFE-Cognition expert panel.

1. **Telephone Interview for Cognitive Status (TICS).** The TICS is a brief test of cognitive functioning, developed to use in situations where in-person cognitive screening is impractical or inefficient.

2. **Dementia Questionnaire (DQ).**[202] The DQ is a questionnaire administered to a proxy/informant either in person or by phone which has been validated against both clinically and pathologically diagnosed dementia.[203, 204] An abbreviated version of the DQ will only be administered to proxies of those participants who do not complete an in-person cognitive assessment, or who score < 30 on the TICS. Additionally, a DQ will be obtained for all deceased participants (within 6 months of death) in order to ascertain cognitive status prior to death.

Additional Measurements
In addition to the cognitive information collected, data collected at the study's baseline and follow-up visits will also be available for diagnostic purposes. This includes demographic information, information on medical history, medication inventory and the results from a baseline physical examination and medical records obtained from the participant's physician(s).

Diagnostic Process
The results from Stage 1 along with the LIFE Cognition screening battery results from baseline and follow-up testing, and the medical records obtained will be forwarded to the Diagnostic Adjudication Committee for the Stage 2 review and diagnosis.

Stage 2: Diagnostic Adjudication by Expert Panel
Prior to the initiating the adjudication process, a meeting of the expert panel will be held at which time the neuropsychological tests used in the assessment, diagnostic criteria and the diagnostic process will be standardized to protect validity and reliability of classifications. A summary of the criteria and rules of adjudication will be provided to adjudicators with each clinical case sent out for review.

All diagnoses will be adjudicated by an expert panel consisting of neurologists, geriatricians, psychiatrists and neuropsychologists with recognized expertise in dementia. The adjudication process will occur in two steps. In Step 1, files containing all the information obtained in the clinical assessment phase, without personal identifying information, will be sent online to two randomly assigned members of the adjudication panel. After review of the data, each adjudicator will record a classification (No Impairment, MCI, Dementia). The panel members will be blind to each other's rating and the treatment assignment of the participants. In Step 2, a periodic face-to-face meeting or phone conference of the expert panel will be held and all cases where there were diagnostic disagreements between panel members will be resolved by the consensus process. This process may involve review of the audio recording obtained during the technician administered assessments.

After careful review of all materials, adjudicators will classify participants into 1 of 3 cognitive categories: No Impairment, Mild Cognitive Impairment or Dementia. Criteria for dementia will be according to DSM-IV as follows:

- Significant decline in memory and at least one additional cognitive domain; and
- Significant functional impairment due to cognitive problems; and
- Cognitive deficits are not due to reversible causes such as metabolic disturbances, infections, nutritional deficits, mood disorders or substance-induced conditions; and
- Cognitive deficits do not occur exclusively during the course of delirium

No attempt to classify dementia subtype will be made.

Criteria for MCI will be those recommended by Winblad et al.

- Observation by participant or proxy of cognitive decline; and
- Deficit in performance in one or more cognitive domain; and
- Absence of significant functional impairment attributable to cognition; and
- No diagnosed dementia

However, MCI will be further sub-classified into 4 categories using criteria adapted from Winblad, et. al as follows:

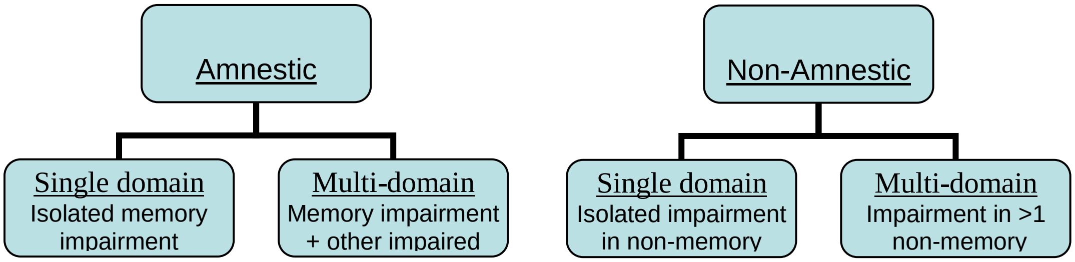

Quality control
Technician training
At each site, two persons will be identified to serve as trained technicians. Technicians administer the LIFE Cognition battery to all participants as well as the FAQ and/or DQ

interview to a proxy if needed. Each technician will be trained during an intensive training session. During this session, technicians will receive instructions about the study protocol and the procedure for administering the tests and interviews by watching didactic presentations on each component followed by engaging in role-play rehearsal with feedback from trainers. Once back at her or his respective site, the technician will practice several administrations and send in an audio-taped administration for central review and evaluation. If the administration is adequate, the technician will receive full certification; if not, she or he will receive feedback and submit a tape again, repeating the process until full certification results. Experienced trainers will review these tapes centrally. Full certification depends on demonstration of appropriate testing skills for all components of the cognitive protocol. During the course of the study, centrally trained and certified technicians will train new technicians. New technicians will be certified by central review in the same manner described above. To prevent significant decay in testing skills, the Life Cognition Coordinating Center will recertify all technicians annually. Audio-taped protocol administrations are once again sent for rating. Technicians failing recertification must re-tape practice sessions until they qualify. Field technicians are encouraged to communicate questions or problems to responsive study staff at the Life Cognition Coordinating Center. Periodic communications will be sent to all certified technicians reminding them of the specific testing challenges and solutions to common problems will arise.

QC of the cognitive assessments will also be ensured by reviewing a 5% sample of audio-taped assessments. Overall consent for the trial will include participant consent for this aspect of QC. Additional QC will include data-based central assessment of the quality and consistency of compliance with the cognitive testing protocol. Deviations from the protocol will trigger re-certification procedures.

Appendix B

LIFE Stopping Guidelines

Summary: We propose to use symmetric O'Brien-Fleming monitoring boundaries for LIFE to monitor the primary outcome and all-cause mortality, both at the 0.05 level. Formal monitoring would be done at each DSMB meeting from April 2011 through April 2013 and at the end of the trial.

Discussion: During the course of the LIFE study we plan to sequentially monitor the primary outcome and all-cause mortality. In many studies, particularly those involving drug treatments, consideration is given to stopping early for harm and for benefit. Stopping early for harm promotes the safety of participants. On the other hand, stopping for benefit would allow those not receiving the beneficial treatment to be switched. Stopping early would also have the advantages of lowering study cost and allowing the results to be promulgated earlier. Quantifying when sufficient information exists to stop a trial early is typically done using statistical monitoring boundaries which will be described shortly.

In a study with an intervention that is believed to have few side effects, such as LIFE's exercise intervention, the decision to stop early may not be so clear. Imagine, for example, that the boundary for benefit has been crossed in LIFE. A convincing argument could be made that the study should be stopped and the main results published. Several arguments could also be made that the trial should be continued. First, stopping the trial early might reduce power for subgroup comparisons, other outcomes (including important secondary outcomes like cognitive decline that make this trial unique), and ancillary studies. Second, sometimes it is useful to continue follow-up to understand the longer-term consequence of interventions and whether shorter-term intervention effects are maintained. This is particularly important in LIFE, as the long-term compliance to the physical activity intervention and the continued benefits of the intervention over several years are unknown and are not being studied in other trials. Third, if it is not feasible to immediately implement the better intervention in those that are receiving the inferior intervention and the inferior intervention is better than returning participants to the community standards, then continuation can provide benefits for everyone in the trial.

WHI Experience: Two publications from *Clinical Trials* on the Women's Health Initiative hormone trials discuss some issues pertinent to LIFE. The first (1) was written by the coordinating center and the NIH project office, the second (2) was written by the DSMB. WHI had a complex monitoring plan in large part because there were many outcomes being assessed. It was expected that the hypothesized benefits of hormone therapy on coronary heart disease (the primary outcome) would take up to three years to manifest while the risk of breast cancer (the primary safety outcome) might take 10 years to be fully realized. Additional outcomes included hip fracture, colorectal cancer, endometrial cancer, stroke, pulmonary embolism, total mortality, and a composite consisting of the first occurrence of any of the conditions (including CHD and breast cancer). (1)

The WHI monitoring plan can be briefly summarized: *"…a discussion of stopping for benefit would be triggered only if both the upper 0.05-level boundary for CHD and the upper 0.10-level boundary for the global index were crossed. Stopping for an overall adverse effect would be considered if any of the disease-specific lower boundaries were*

crossed (0.10-level for breast cancer or the Bonferroni corrected 0.10-level for other listed outcomes) and the global index logrank statistic was less than -1.0." (1)

The monitoring plan for WHI was based on *"…weighted logrank statistics … but an unweighted Cox regression analysis was used in publishing … motivated by the importance of providing a transparent and easily interpretable (hazard ratio) estimate of treatment effects."* (1) Even though the monitoring plan was asymmetric, *"… to incorporate this asymmetry into confidence intervals would have been both awkward and inconsistent with reporting standards, so the wider 95% CIs were used uniformly for all adverse effects."* (1)

Like LIFE, WHI was studying prevention rather than treatment and this led to the asymmetric boundaries: *"The lack of immediate medical benefit required us to have a lower threshold for harm. We thought that termination of the trials before the availability of clearly persuasive answers could have an undesirable effect on the practice of medicine that could affect the health of millions of women."* (2)

Discussion: We have considered the use of asymmetric monitoring boundaries such as using 0.01 for benefit and 0.04 for harm. While this would provide the benefit of making it more difficult to stop early for benefit, an argument could certainly be made that logical consistency would require asymmetric confidence intervals at the end of LIFE. To make a claim that the physical activity intervention was better than healthy aging control, this would require that the evidence in that direction be significant at 0.02 (twice 0.01) instead of 0.05. For the same reasons this was not done in WHI, we do not recommend this approach for LIFE

All monitoring boundaries are provided primarily as a guide to the DSMB. A DSMB must integrate all information, including information external to the study, when making a recommendation. The DSMB can recommend continuation when a boundary has been crossed or discontinuation when a boundary has not been crossed. Even if a boundary has been crossed, the study can still continue and appropriate adjusted p-values can be calculated at the end of the study. We will report both the nominal and adjusted p-values in the final manuscript

Conclusion: We propose that we use a two-sided symmetric monitoring approach in LIFE for the primary outcome and for total mortality, both at the 0.05 level. Specifically, we propose to use a two-sided O'Brien-Fleming boundary for the hazard ratio from the Cox model that will be used for the primary analysis. We anticipate that the LIFE DSMB will begin monitoring the trial at the second DSMB meeting in April 2011 and will monitor every six months thereafter. The October 2013 monitoring would be skipped as the trial would be ending in two months anyway. The table below presents O'Brien-Fleming boundaries as calculated by East 5.3 (www.cytel.com).

In addition, the LIFE Steering Committee proposes that the DSMB strongly consider not stopping the trial for early benefit for the primary outcome so that secondary outcomes can be assessed, long-term compliance with the intervention can be evaluated and the benefits of the intervention over an extended period of time can be estimated. This large, complex trial is very unlikely to be repeated in the future so it is likely that this will be the only opportunity to investigate these important research questions.

Table. Proposed dates of monitoring, percent information available, the Z criticial levels, and the nominal p-values.

DSMB Meeting Date	% Information Available	Z critical level	Nominal P-value
April 2011	6.7	±8.580	9.5×10^{-18}
October 2011	19.3	±4.969	6.7×10^{-7}
April 2012	37.4	±3.484	4.9×10^{-4}
October 2012	56.9	±2.761	5.8×10^{-3}
April 2013	74.5	±2.388	0.017
Final (December 2013)	100	±2.014	0.044

Figure. Proposed monitoring boundaries.

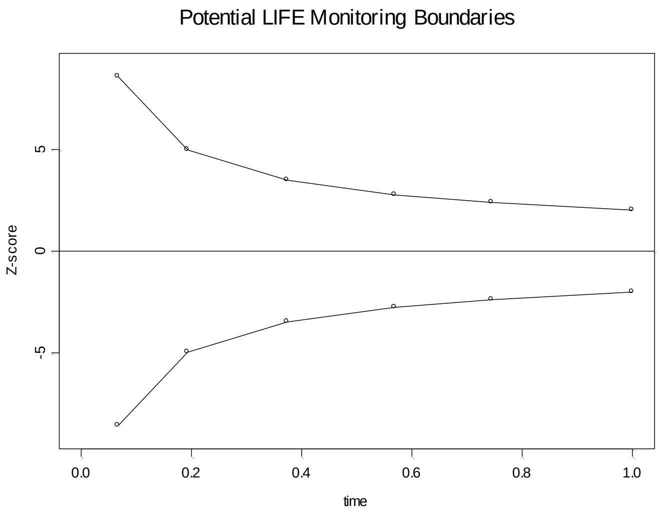

References:

1. Anderson GL, Kooperberg C, Geller N, Rossouw JE, Pettinger M, Prentice RL. Monitoring and reporting of the Women's Health Initiative randomized hormone therapy trials. Clinical Trials, 2007, 4:207-217.
2. Wittes J, Barrett-Connor E, Braunwald E, Chesney M, Cohen HJ, DeMets D, Dunn L, Dwyer J, Heaney RP, Vogel V, Walters L, Yusuf S. Monitoring the randomized trials of the Women's Health Initiative: the experience of the Data and Safety Monitoring Board. Clinical Trials, 2007, 4:218-234.
